# Targeting Cytotoxic Agents through EGFR-Mediated Covalent Binding and Release

**DOI:** 10.1021/acs.jmedchem.3c00845

**Published:** 2023-08-30

**Authors:** Pasquale A. Morese, Nahoum Anthony, Michael Bodnarchuk, Claire Jennings, Mathew P. Martin, Richard A. Noble, Nicole Phillips, Huw D. Thomas, Lan Z. Wang, Andrew Lister, Martin E. M. Noble, Richard A. Ward, Stephen R. Wedge, Hannah L. Stewart, Michael J. Waring

**Affiliations:** † Cancer Research Horizons Therapeutic Innovation, Newcastle University Centre for Cancer, Chemistry, School of Natural and Environmental Sciences, Newcastle University, Bedson Building, Newcastle upon Tyne NE1 7RU, U.K.; ‡ Cancer Research Horizons Therapeutic Innovation, Newcastle University Centre for Cancer, Translational and Clinical Research Institute, Faculty of Medical Sciences, Paul O’Gorman Building, Newcastle University, Newcastle upon Tyne NE2 4HH, U.K.; § Oncology iMed, R&D, AstraZeneca, Cambridge CB4 0WG, U.K.

## Abstract

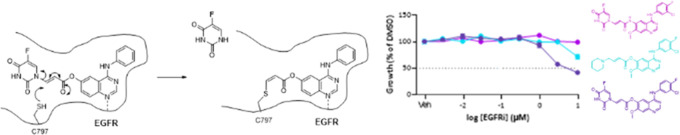

A major drawback of cytotoxic chemotherapy is the lack of selectivity toward noncancerous cells. The targeted delivery of cytotoxic drugs to tumor cells is a longstanding goal in cancer research. We proposed that covalent inhibitors could be adapted to deliver cytotoxic agents, conjugated to the β-position of the Michael acceptor, via an addition–elimination mechanism promoted by covalent binding. Studies on model systems showed that conjugated 5-fluorouracil (5FU) could be released upon thiol addition in relevant time scales. A series of covalent epidermal growth factor receptor (EGFR) inhibitors were synthesized as their 5FU derivatives. Achieving the desired release of 5FU was demonstrated to depend on the electronics and geometry of the compounds. Mass spectrometry and NMR studies demonstrated an anilinoquinazoline acrylate ester conjugate bound to EGFR with the release of 5FU. This work establishes that acrylates can be used to release conjugated molecules upon covalent binding to proteins and could be used to develop targeted therapeutics.

## Introduction

The selective delivery of cytotoxic agents to tumor cells to maximize efficacy and minimize effects on normal cells is a longstanding goal of cancer chemotherapy.^1−4^ While conceptually simple, molecular targeting of cancers requires the identification of a specific mechanism to selectively localize a therapeutic agent within a tumor and for the agent to deliver a cytotoxic effect once it has reached its site of action. Identification of an appropriate mechanism is challenging due to the similarities between tumor and normal cells.

**Figure 1:**
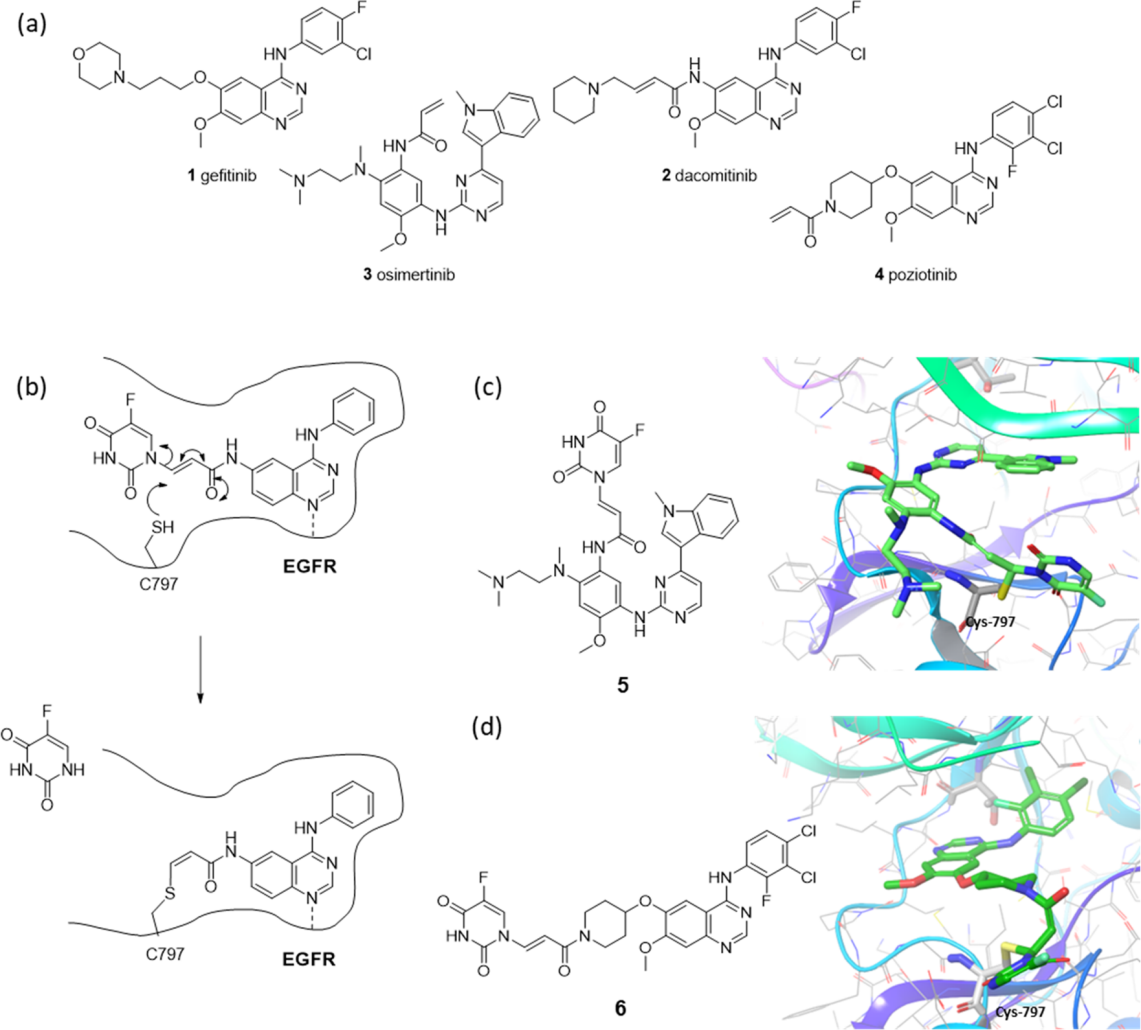
(a) Examples of established EGFR inhibitors; (b) depictions of proposed 5FU conjugates with the covalent binding/release process in the EGFR active site; models of bound conformations of (c) pyrimidine- and (d) quinazoline-based conjugates in the EGFR active site based on PDB structures 4ZAU and 4G5J, respectively.

The epidermal growth factor receptor (EGFR) is a receptor tyrosine kinase that is commonly mutated in non-small cell lung cancer (NSCLC).^5,6^ Activating mutations in EGFR such as the exon-19 deletion and the L858R point mutation are key oncogenic drivers, and tumors harboring these mutations have been shown to be amenable to treatment with small molecule EGFR inhibitors.^7^ The first compounds to be used clinically were the reversible inhibitors gefitinib **1**(8) and erlotinib,^9^ but subsequently irreversible inhibitors such as dacomitinib **2**,^10^ osimertinib **3**,^11^ and poziotinib **4**(12) have become prominent and are effective treatments for EGFR-driven NSCLC (Figure 1a).

The irreversible EGFR inhibitors function by targeting cysteine residue C797, which reacts via a conjugate addition to the pendant acrylamides upon binding of the inhibitor to the adenosine triphosphate (ATP) pocket.^13^ We reasoned that this binding event could be used as a targeting mechanism, and that if a cytotoxic agent was appended to the β-position of the acrylamide, an addition–elimination process could trigger its specific release (Figure 1b). We selected 5-fluorouracil (5FU) as the cytotoxic agent for initial studies since its small size would minimize the perturbation of the physical properties of the targeting inhibitor and would be most likely to be tolerated in the active site. Additionally, 5FU could be linked via the 1-nitrogen in a manner that would retain the reactivity of the Michael acceptor and act as a suitable leaving group due to its electron-deficient nature. Modeling of the conjugates of osimertinib **5** and poziotinib **6** in the active site suggested that they should be tolerated (Figure 1c,d).

Accordingly, we set out to investigate the synthesis, binding, and release of 5FU-EGFR inhibitor conjugates to assess their potential to provide a targeted delivery paradigm. During the course of this work, a similar approach, termed covalent ligand-directed release (CoLDR) involving the attachment of releasable ligands on the methyl position of α-methacrylamide inhibitors, was disclosed by London^14,15^ and Fang.^16^ Our concept of conjugating inhibitors at the β-position would be complementary to this approach and would offer versatility with regard to the tolerability of the conjugates’ target binding and their release kinetics.

## Results

### Synthesis of Model Release Systems

To determine the feasibility of selectively releasing a cytotoxic drug as a leaving group via conjugate addition–elimination, model release systems were prepared. The purposes of this were twofold; first, they would provide evidence that the release could be achieved controllably in the presence of a cysteine residue, and second, to investigate the tuning of the respective systems with modifications to the electronic properties of the Michael acceptors.

There were no reported examples in the literature in which a thiol has been able to displace a leaving group from the β-position of an enamide. There were, however, numerous reports of the analogous reaction on a vinyl ketone. The most relevant reaction reported is of *N*-acetyl cysteine with phenyloxybut-2-enones, in which a detailed kinetic study was described under conditions that closely resemble those encountered *in vivo.*(17) Ester, amide, and ketone activating groups were hypothesized to be the best suited to allow the release of the cytotoxic payload, and so model systems were proposed with each of these functionalities, along with varied electron-donating and -withdrawing groups on the aryl substituent.

**Scheme 1:**
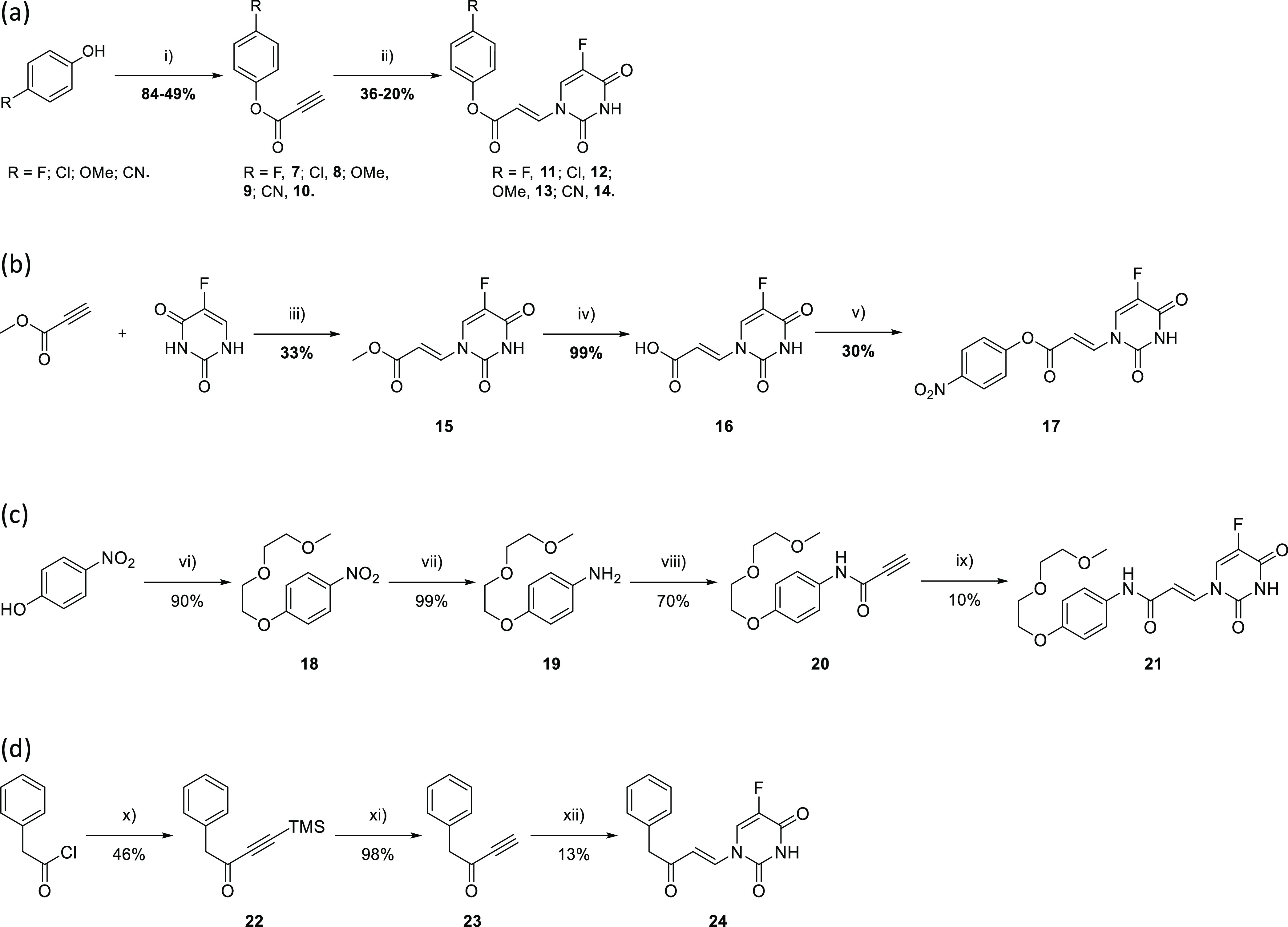
Synthesis of Enamide and Acrylate Model Systems Reagents and conditions: (i) HATU or dicyclohexylcarbodiimide (DCC), 4-dimethylaminopyridine (DMAP), *N*,*N*-dimethylformamide (DMF), 0 °C—rt, 18 h; (ii) 5FU, DABCO (cat.), DMF, rt—40 °C, 1–24 h; (iii) DABCO, DMF, 0 °C, 1.5 h; (iv) 2 M NaOH (aq), tetrahydrofuran (THF), rt, 30 min; (v) 4-nitrophenol, DCC, DMAP, DMF, 5 h, rt; (vi) 1-(2-(2-methoxyethoxy)ethoxy)-4-tosylate, K_2_CO_3_, DMF, 80 °C, 3 h; (vii) H_2_, Pd/C (10% wt), MeOH/dichloromethane (DCM), 40 °C 21 h; (viii) propiolic acid, HATU, DMAP, DMF, 0 °C—rt, 18 h; (ix) 5FU, DABCO, DMF, 40 °C, 40 h; (x) bis(trimethylsilyl)ethylene, AlCl_3_, DCM, 0 °C—rt, 18 h; (xi) tetra-*n*-butylammonium fluoride (TBAF), MeOH, −78 °C, 1 h; (xii) 5FU, DABCO, DMF, 40 °C, 30 min.

Synthesis of the desired esters began with the corresponding phenol coupled with propiolic acid to give propargyl esters **7**–**10**, followed by the DABCO-promoted addition of 5FU to afford conjugates **11**–**14** (Scheme 1a). Synthesis of the analogous nitro-containing acrylate was not feasible through this route but was prepared by the initial addition of 5FU to methyl propiolate to give ester **15**, followed by hydrolysis to the corresponding acid **16** and coupling with 4-nitrophenol to give conjugate **17** (Scheme 1b).

Aqueous solubility of the corresponding amides was too low to allow the NMR experiments (see below); therefore, an analogue containing a solubilizing bis-ethylene glycol chain was prepared (Scheme 1c). Etherification of 4-nitrophenol gave bisethyleneglycol **18** followed by reduction to aniline **19** to enable coupling with propiolic acid to give propargyl amide **20**. DABCO-promoted addition with 5FU gave conjugate **21**.

Synthesis of the enone began with installing TMS acetylene to the starting phenylacetyl chloride to give ketone **22**, followed by removal of the TMS protecting group using TBAF in methanol to give then ynone **23**. 5FU was added in the presence of DABCO resulting in target enone **24** (Scheme 1d).

### ^19^F NMR Cytotoxic Release Studies

**Figure 2:**
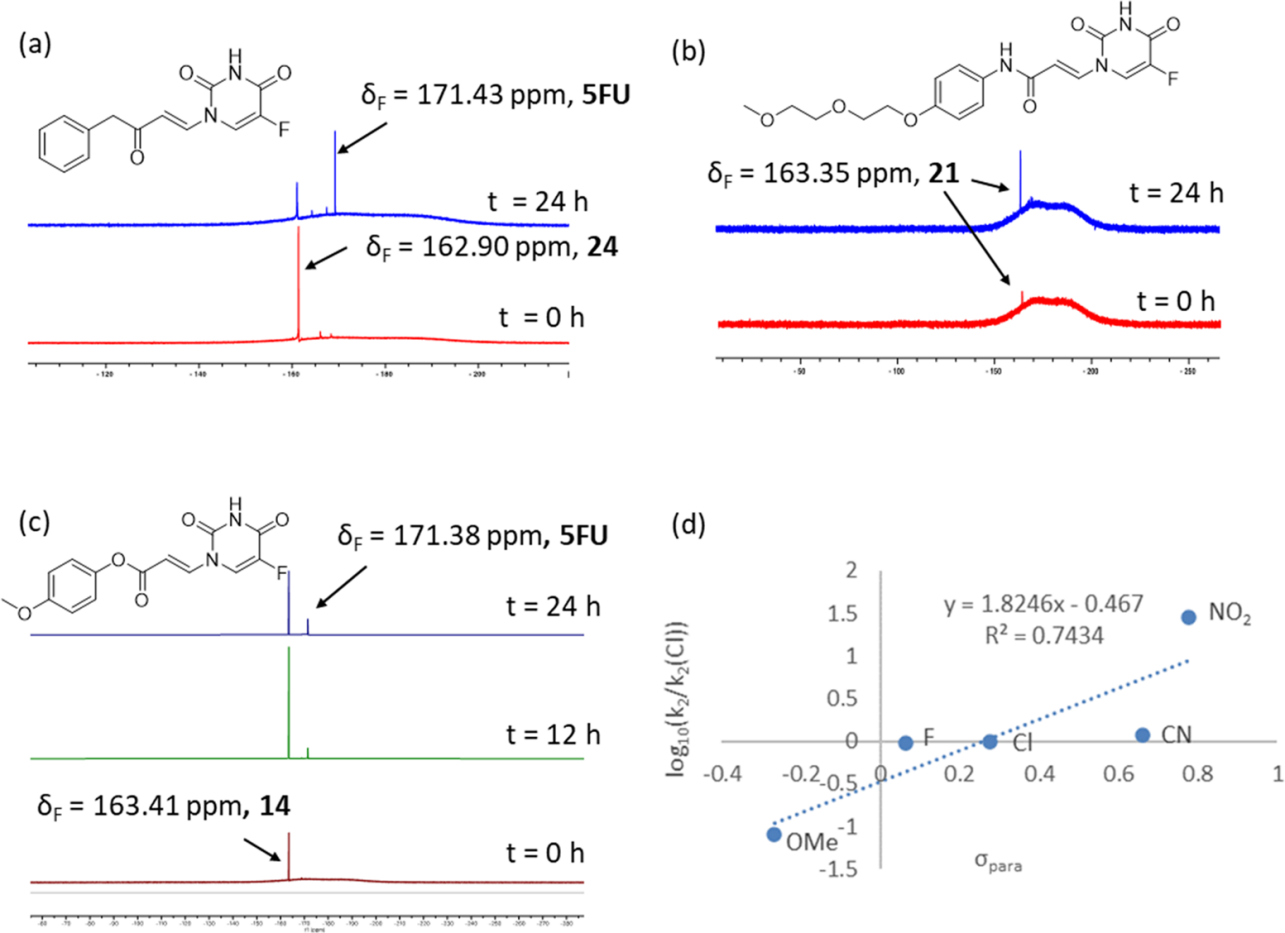
^19^F NMR binding/release studies of model conjugates by incubation with methyl acetyl cysteine. ^19^F NMR spectra of (a) ketone **24** and (b) amide **21**, (c) methoxy-substituted acrylate **13**, and (d) Hammett relationship for the para-substituted acrylates **12**–**14** and **17**.

Reaction of the model systems with *N*-acetyl-l-cysteine methyl ester was assessed by ^19^F NMR. Observation of a ^19^F NMR signal corresponding to 5FU indicated that the release of the cytotoxic had occurred. Ketone **24** showed the formation of 5FU with a half-life of approximately 10 h (Figure 2a). PEGylated amide **21** showed no detectable release within the time scale of the experiment (Figure 2b).

While it is likely that the rate of elimination would be increased within the context of binding to the target protein, these studies indicated that the rate of release was dependent on the electronic properties of the Michael acceptor. Consistent with this, the corresponding methoxy-substituted acrylate (with electronics intermediate between **21** and **24**) showed a small amount of release after 24 h (half-life ∼36 h; Figure 2c and Table 1). For acrylates, the rate showed an approximately linear Hammett dependency on the electronics of the para substituent (ρ = 1.82; Figure 2d). Importantly, the range of half-lives could be modulated between 36 h (−OMe) and 0.1 h (−NO_2_), indicating that it should be possible to achieve the desired release kinetics in compounds of this type.

**Table 1:** Half-Life of Elimination of 5FU for Phenylacrylate Derivatives

cpd.	R	*t*_1/2_ (h)
**11**	F	3.0
**12**	Cl	2.9
**13**	OMe	36
**14**	CN	2.5
**17**	NO_2_	0.1

### Formation of EGFR Inhibitor-Cytotoxic Hybrids

**Scheme 2:**
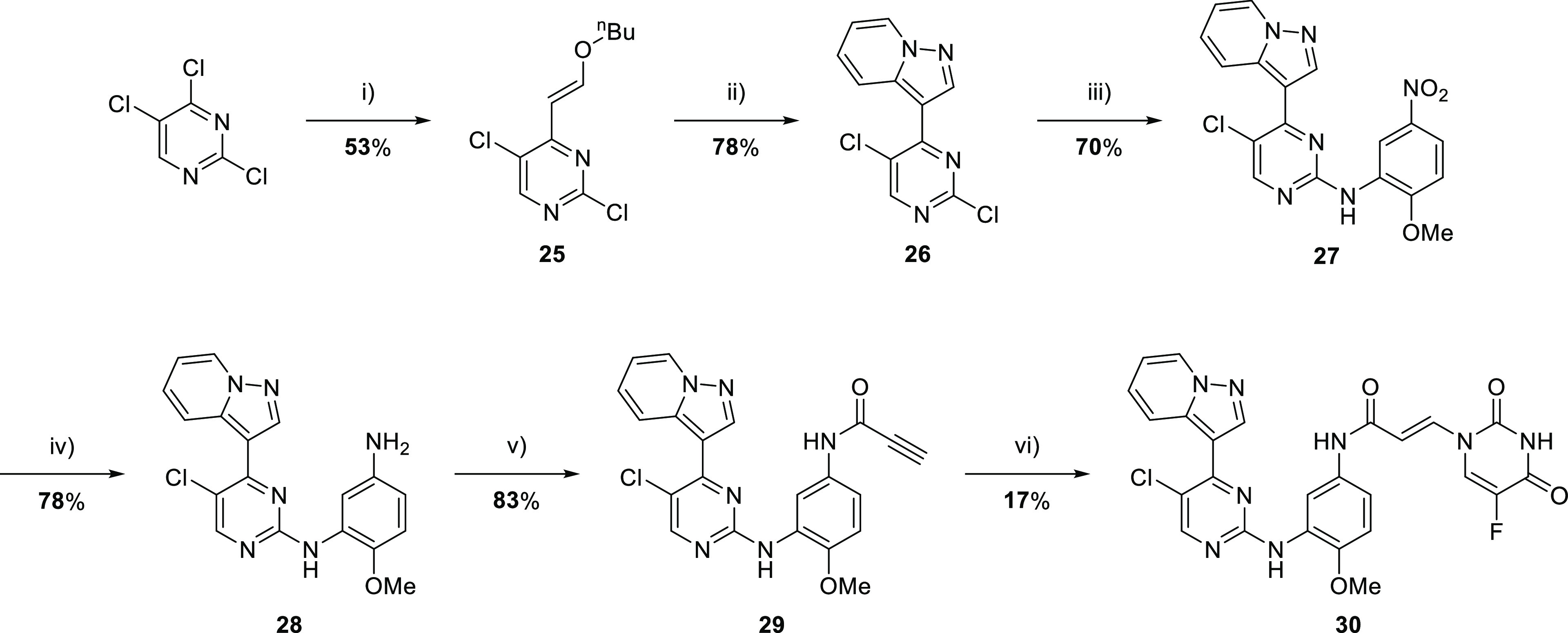
Synthesis of the Enamide Inhibitor-Cytotoxic Hybrid Compound **30** Reagents and conditions: (i) Et_3_N, butyl vinyl ether, Pd(OAc)_2_, PEG-200, 80 °C, 1 h; (ii) 1-aminopyridinium iodide, K_2_CO_3_, *N*,*N*-dimethylacetamide (DMA), 110 °C, 18 h; (iii) 2-methoxy-5-nitroaniline, p-TSA, 2-pentanol, 48 h, 110 °C, (iv) Zn, NH_4_Cl, MeOH/H_2_O (9:1, v/v), 70 °C, 4 h; (v) propiolic acid, HATU, *N*,*N*-diisopropylethylamine (DIPEA), DMF, rt, 4 h; and (vi) 5FU, DABCO, DMF, 1 h, 40 °C.

Having established that the desired release was achievable in a relevant time scale, we synthesized the corresponding elaborated EGFR inhibitor-5FU hybrids. Initially, we focused on pyridinylpyrazolopyrimidine-based inhibitors, such as **30** (Scheme 2). This template had been shown previously to give potent EGFR inhibitors.^18^

**Scheme 3:**
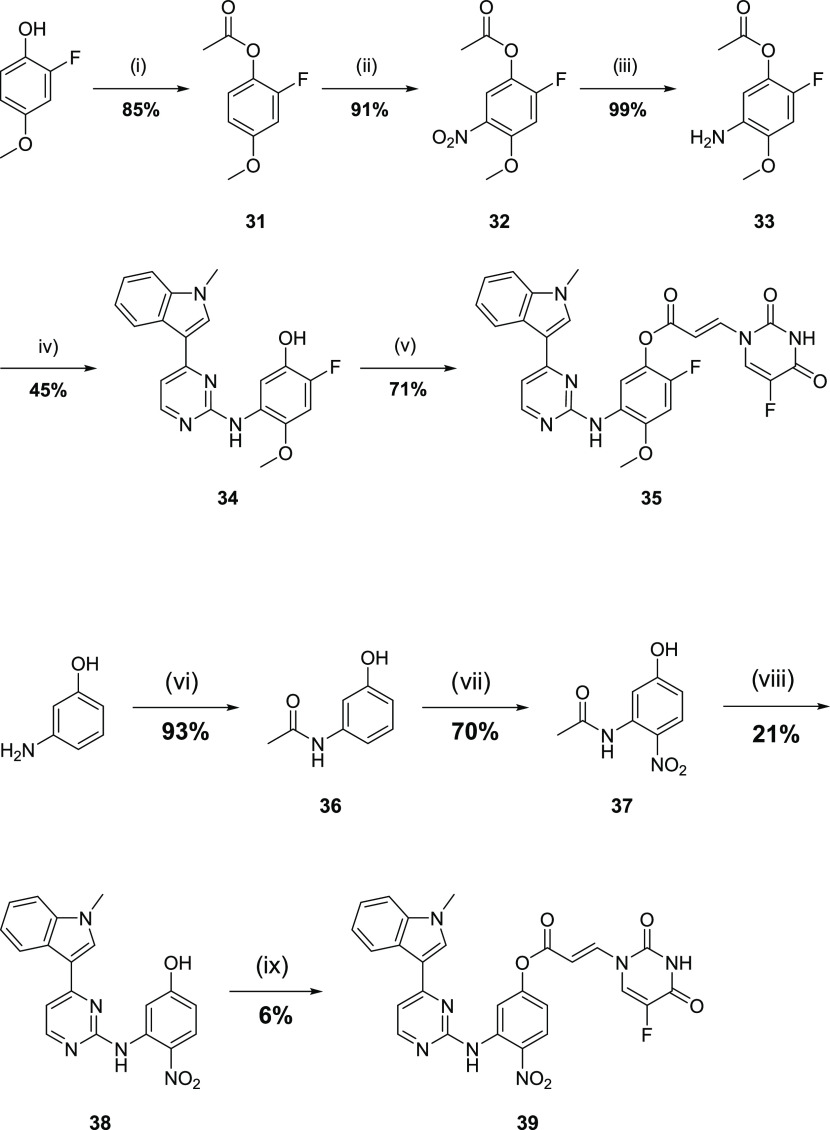
Synthetic Route for the Formation of the Anilinopyrimidine Acrylates Reagents and conditions: (i) acetyl chloride, Et_3_N, DCM, rt, 18 h; (ii) HNO_3_, H_2_SO_4_, AcOH, 0 °C; 1 h, (iii) H_2_, Pd/C, MeOH, rt, 24 h; (iv) 3-(2-chloropyrimidin-4-yl)-1-methyl-1*H*-indole,^18^*p*-TSA, 2-pentanol, 110 °C, 18 h; (v) **16**, DCC, DMAP, DMF, 0 °C—rt, 5 h; (vi) Ac_2_O, THF, rt, 1 h; (vii) KNO_3_, trifluoroacetic acid (TFA), 0 °C, 1 h; (viii) (a) 12 M HCl (aq), reflux, 2 h, (b) 3-(2-chloropyrimidin-4-yl)-1-methyl-1*H*-indole, p-TSA, 2-pentanol, 110 °C, 18 h; and (ix) **16**, DCC, DMAP, DMF, rt, 18 h.

Ester analogues were prepared with a pyrimidinyl indole EGFR inhibitor motif, also precedented to give potent EGFR inhibitors, including osimertinib (Scheme 3).^11,19^

Target molecule **30** was prepared in 6 overall steps from 2,4,5-trichloropyrimidine, commencing with the Heck-type coupling with butyl vinyl ether to give enol-ether **25** (Scheme 2). This compound was then reacted with 1-aminopyridinium iodide to give pyrazolopyrimidine **26**, which underwent S_N_Ar reaction with 2-methoxy-5-nitroaniline-5-nitroaniline to give anilinopyrimidine **27**. Nitro reduction gave aniline **28**, which was coupled with propiolic acid to give propiolamide **29**. The desired conjugate was prepared by the DABCO-mediated addition of 5FU to give **30**.

Synthesis of 2-fluoro-4-methoxyacrylate derivative **35** started from 2-fluoro-4-methoxyphenol, which was esterified to give acetate **31** (Scheme 3). The acetate underwent nitration with the desired regiochemistry to give **32**, which underwent reduction to aniline **33**. S_N_Ar reaction of **33** with 3-(2-chloropyrimidin-4-yl)-1-methyl-1H-indole proceeded with concomitant acetate hydrolysis to give the phenol precursor **34**. The synthesis of the desired conjugate achieved the DCC-mediated coupling of **34** with **16**.

The corresponding 4-nitroacrylamide was prepared from 3-aminophenol by acetamide protection to give **36**, which underwent nitration to give **37** (Scheme 3). Acetamide hydrolysis revealed the aniline, which underwent S_N_Ar reaction to give phenol **38**. Compound **38** was esterified with **16** to afford conjugate **39**. The enone analogue of **39** proved too reactive leading to difficulties in the final purification and so was not profiled further.

Incubation of compound **30** with EGFR protein and subsequent analysis by mass spectrometry showed that an adduct formed and was confirmed by protein digestion and peptide mapping to be the result of reaction with C797, confirming that the 5FU conjugate was sufficiently reactive and tolerated sterically within the active site (Figure S1). However, only the adduct was observed and there was no evidence of the release of 5FU. Both acrylates **35** and **39** also showed evidence of adduct formation by mass spectrometry, again without evidence of the release of 5FU in both cases (Figures S2 and S3). Interestingly, methoxy derivative **35** showed more extensive labeling than the more electron-deficient (and therefore more reactive) nitro derivative **39**. Hence, despite the addition being feasible, this was not sufficient to achieve the desired subsequent elimination in the environment of the bound state.

### Anilinoquinazoline-Based EGFR Inhibitor-Cytotoxic Hybrids

**Scheme 4:**
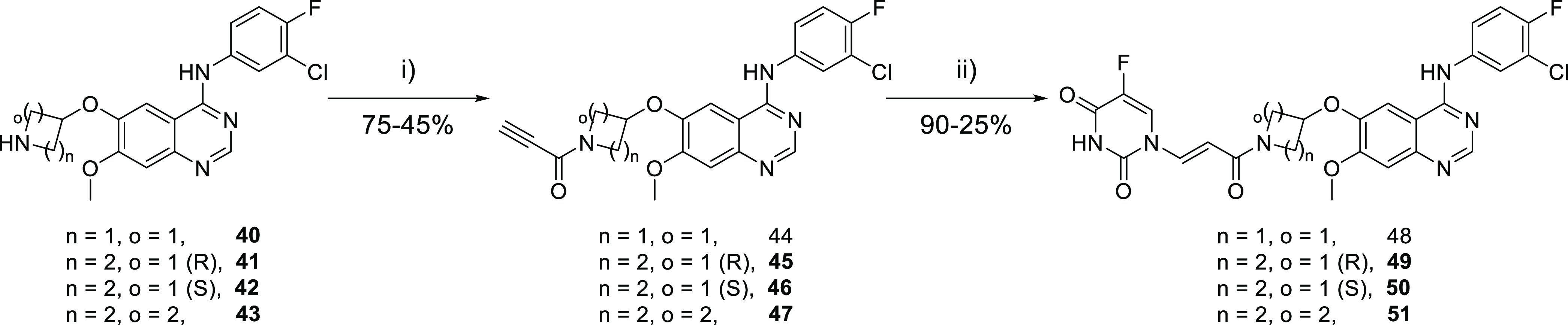
Synthesis of the Anilinoquinazoline Amides Reagents and conditions: (i) propiolic acid, HATU, DIPEA, DMA, 0 °C, 2 h; (ii) 5FU, DABCO, DMF, 50 °C, 18 h.

The anilinoquinazoline-derived covalent inhibitors, exemplified by afatinib **2** and poziotinib **4**, provided an alternative scaffold for a cytotoxic conjugate with different geometries, which we considered worth exploring to assess if this facilitated the required elimination. Accordingly, we prepared a series of covalent acrylamide anilinoquinazoline-5FU conjugates with varying geometries in the ring linking the warhead to the quinazoline core via 4-, 5-, and 6-membered rings **48**–**51** (Scheme 4). Synthesis was performed by amide coupling from known intermediates **40**–**43**^20,21^ by amide coupling with propiolic acid to give propiolates **44**–**47** followed by the addition of 5FU to the ynamide Michael acceptor.

**Scheme 5:**
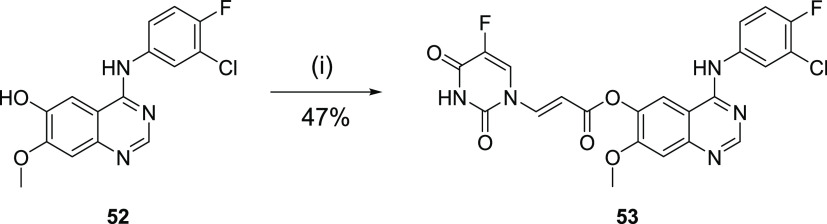
Synthesis of Anilinoquinazoline Acrylate Reagents and conditions: (i) **16**, DCC, DMAP, DMF, 0 °C—rt, 18 h.

**Scheme 6:**
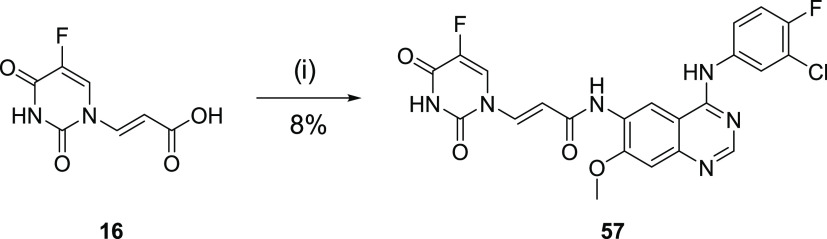
Synthesis of Anilinoquinazoline Acrylamide Reagents and conditions: (i) (a) (COCl)_2_, DMF, THF, rt, 1 h, (b) (*E*)-3-(5-fluoro-2,4-dioxo-3,4-dihydropyrimidin-1(2*H*)-yl)acrylic acid, **58**, pyridine, THF, rt, 12 h.

We also prepared the acrylate ester analogue **53**. The acrylate ester, **53**, was conveniently available via a single-step esterification of intermediate phenol **52** with carboxylic acid **16** (Scheme 5). Acrylamide, **57**, was also synthesized due to the potential metabolic instability of acrylate ester **53**, accessed in one-pot via the acyl chloride derivative of carboxylic acid **16** and aniline **58** (Scheme 6).

**Figure 3:**
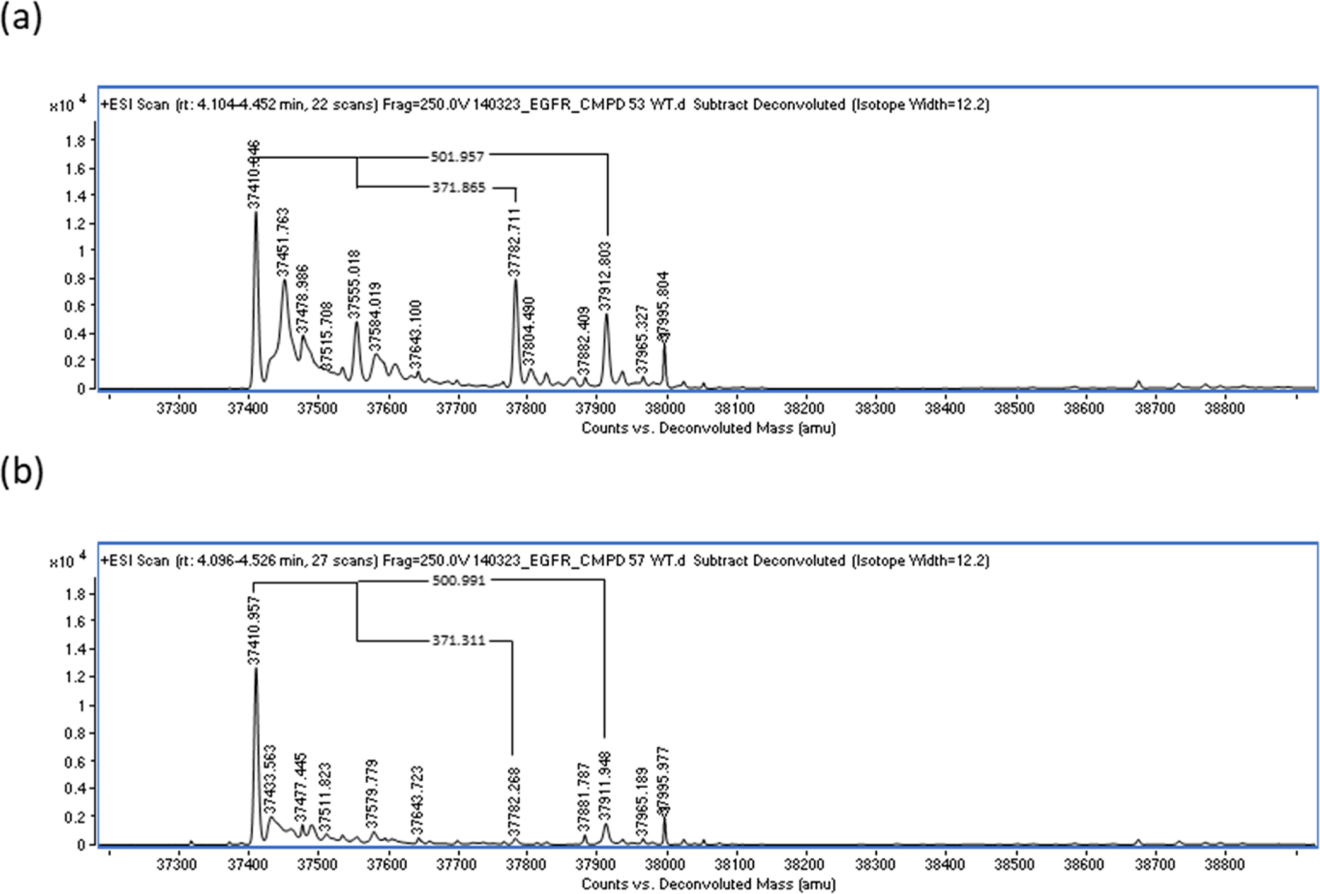
Protein mass spectrometry spectra after incubation of WT protein with (a) **53** and (b) **57**.

Incubation of acrylamide **48** with EGFR and subsequent mass spectrometry analysis again indicated the covalent adduct formation without evidence of the release of 5FU (Figure S4). The other acrylamide conjugates **49**–**51** did not show any evidence of adduct formation (Figure S5) despite showing potent EGFR inhibition (Table S1). In contrast to all previous compounds, protein mass spectrometry of acrylate **53** after incubation with WT EGFR revealed two new major adducts corresponding to the initial adduct (mass increase of 502 Da) and the lower mass peak equating to the loss of 5FU from the complex (increase of 372 Da relative to apo protein) (Figure 3a). This provided clear evidence that the desired release of 5FU is feasible for this compound.

Acrylamide analogue **57** demonstrated a clear adduct with WT EGFR (mass increase of 501 Da), with a negligible release of 5FU (mass increase of 371 Da relative to apo protein), suggesting a much slower retro-Michael addition (Figure 3b).

**Figure 4:**
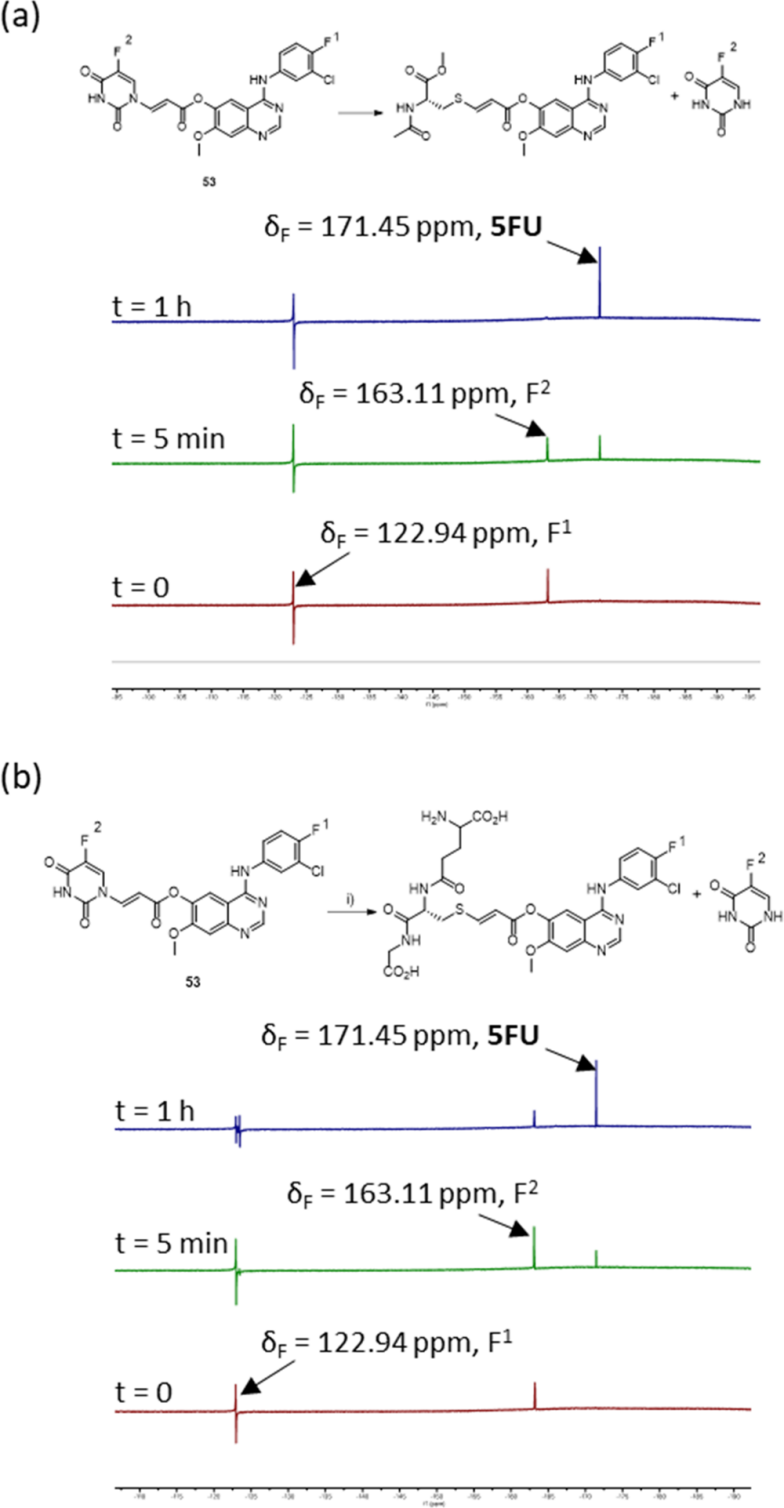
Addition–elimination reaction. ^19^F NMR studies of **53** with (a) *N*-acetyl-l-cysteine methyl ester and (b) glutathione.

To confirm the release of 5FU, the reactions of **53** and **57** with thiol nucleophiles *N*-acetyl cysteine and glutathione were studied by ^19^F NMR. As expected, on reaction with *N*-acetyl cysteine, **57** showed no evidence of 5FU release even after 24 h. However, **53** was shown to rapidly release 5FU, with a half-life of ca. 5 min and a complete release of 5FU after 1 hour (Figure 4a). This level of reactivity is comparable to that observed with model nitro ester **17**. Reaction with glutathione proceeded in a similar manner, although at a slightly slower rate, with some levels of **53** remaining even after 1 h (Figure 4b).

### Biological Evaluation of the Inhibitor-Cytotoxic Hybrids

**Figure 5:**
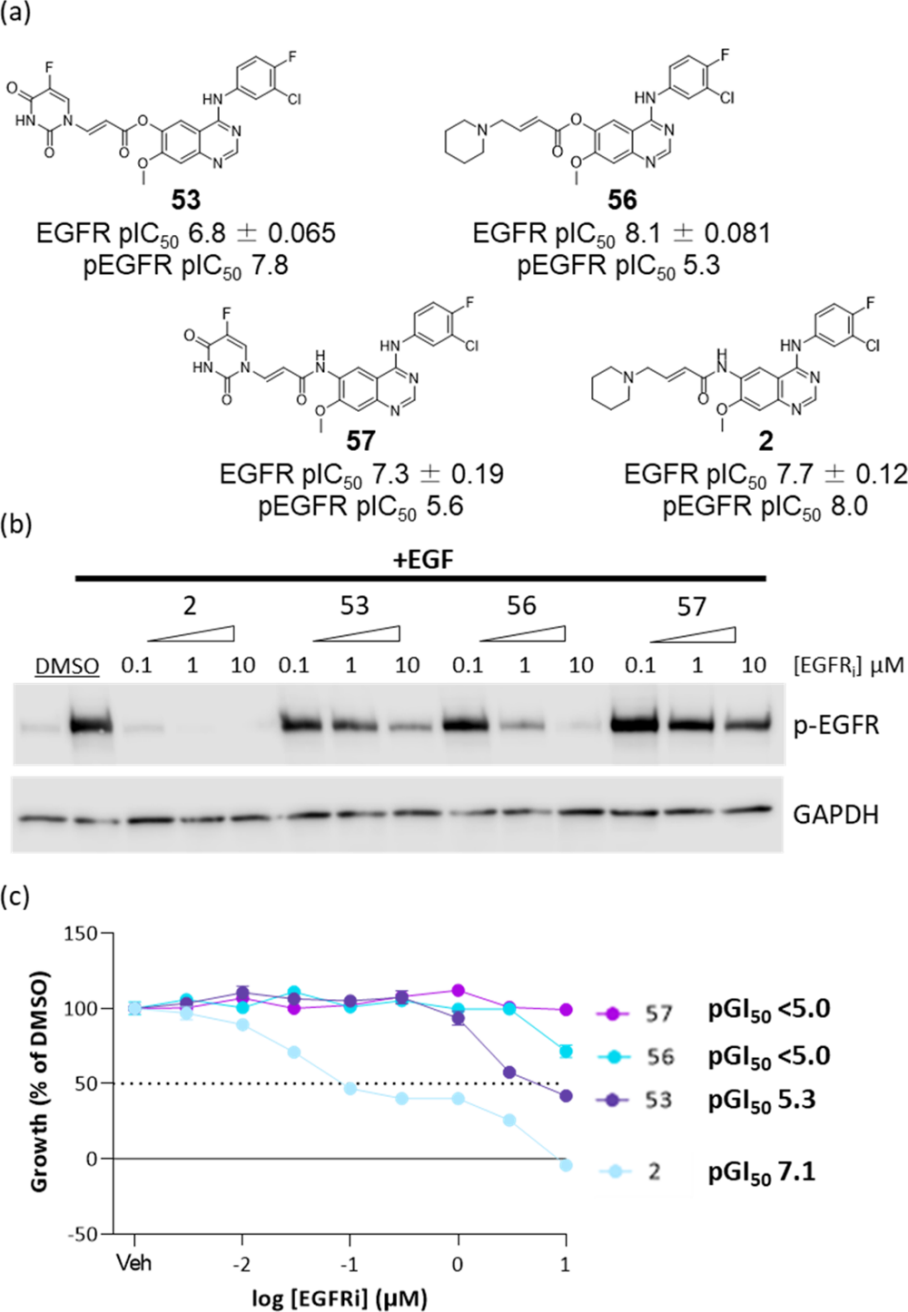
(a) Structures and WT EGFR potencies of compounds **2**, **53**, **56**, and **57** determined by time-resolved fluorescence resonance energy transfer (TR-FRET) (*n* > 2) and cellular pEGFR inhibition determined by HTRF; (b) pEGFR inhibition determined by Western blot in A431 (4 h); and (c) concentration response curves of the A431 cell viability in response to inhibitors (72 h).

The acrylate-5FU conjugates **53** and **57** were profiled in biological assays for EGFR activity alongside dacomitinib **2** and its corresponding acrylate analogue **56** (Scheme S1). Both **2** and **56** were potent inhibitors of EGFR *in vitro* (Figure 5a). Conjugate **53** was slightly less potent but retained activity (pIC_50_ 6.8), while conjugate **57** was slightly more potent (pIC_50_ 7.3).

In A431 cells, conjugate **53** showed a marked inhibition of EGFR phosphorylation at 10 μM but not at 1 μM (Figure 5b), with a pIC_50_ 7.8. This level of inhibition was better than ester **56** (pIC_50_ 5.3), despite demonstrating some inhibition of EGFR phosphorylation at 1 μM. Whereas **2** was more potent as expected (pIC_50_ 8.0), **57** showed little inhibition of EGFR phosphorylation even at 10 μM and a pIC_50_ of 5.6. In 72 h of growth inhibition assays, conjugate **53** showed a potent antiproliferative effect (pGI_50_ 5.3; Figure 5c). This was a marked increase in potency compared to ester **56** (pGI_50_ < 5.0). Disappointingly, **57** showed no antiproliferative effects, despite the strong antiproliferative effects demonstrated by its corresponding analogue **2** (pGI_50_**7.1**).

These data provide clear evidence that **53** binds to EGFR in a cellular setting. The reduced potency of ester **56** for the inhibition of EGFR phosphorylation and growth inhibition relative to its *in vitro* potency is likely a consequence of its increased reactivity and competitive reaction with glutathione in a cellular environment.^15^

The increased growth inhibition potency of conjugate **53** relative to **56** is likely in part a consequence of the reduced reactivity of the warhead from introducing the 5FU substituent to the acrylate. However, the improved growth effects of **53** may also imply an additional effect from the release of 5FU.

**Figure 6:**
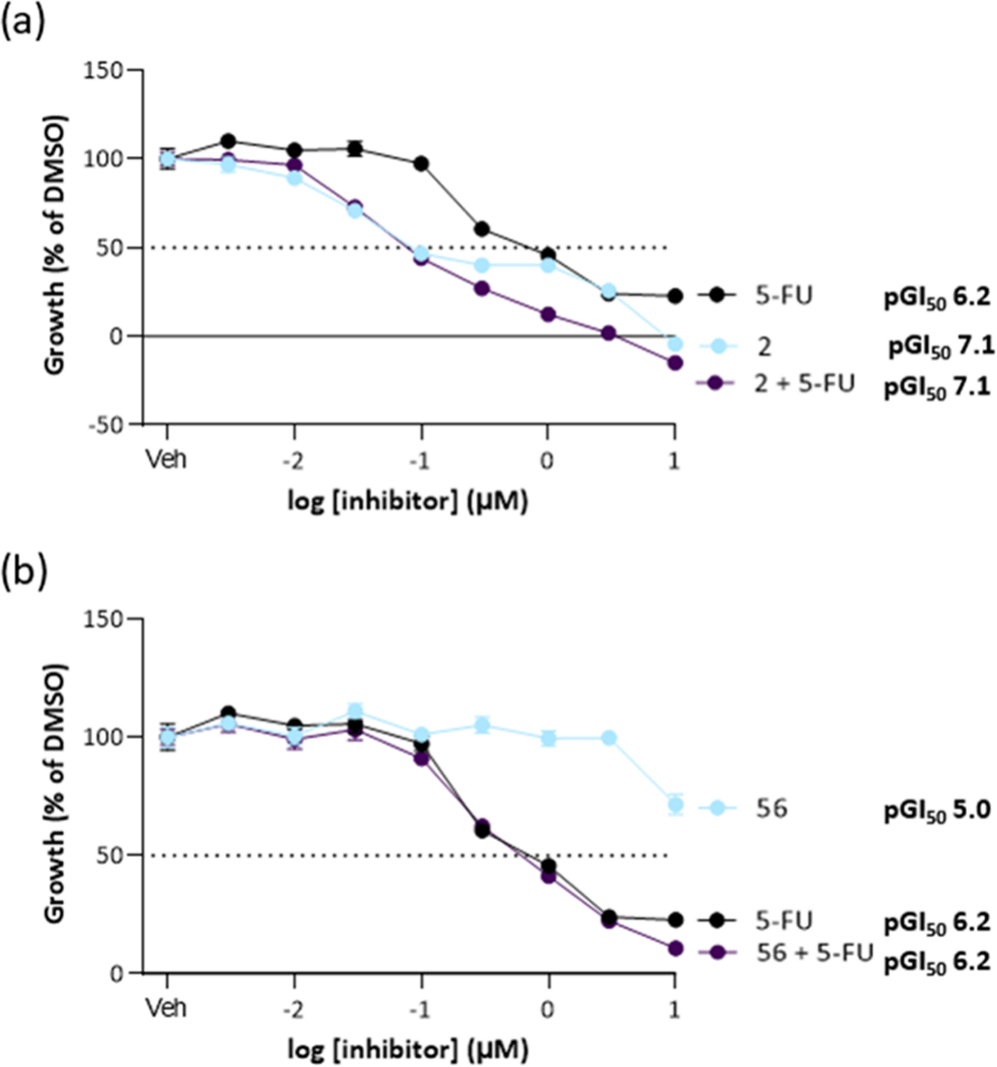
(a) Concentration response curves of the A431 cell viability in response to inhibitor **2**, 5FU and equimolar **2** and 5FU (72 h); (b) concentration response curves of the A431 cell viability in response to inhibitor **56**, 5FU and equimolar **56** and 5FU (72 h).

In growth inhibition assays, an increase in potency is seen for **2** combined with 5FU compared to either **2** or 5FU alone (Figure 6a) and for **56** in combination with 5FU compared to **56** alone but comparable to 5FU alone (Figure 6b). It was expected that this would mimic the effect of the proposed release of 5FU from **53** or **57**, with the observed increase in potency providing additional evidence that the growth effects of **53** compared to those of **56** come from the release of 5FU.

Acrylate conjugate **53** was incubated in cell media to demonstrate that the release of 5FU must be catalyzed by EGFR and does not simply occur in the media. For 25 h, some hydrolysis did occur; however, it was the ester bond that was hydrolyzed: there was no observation of the release of 5FU (Figure S6). Given the significantly slower release of 5FU for acrylamide **57**, a suitable bioisosteric replacement of an ester with comparable electronic properties would be necessary for compounds with *in vivo* stability.

## Discussion

Protein mass spectrometry of EGFR after incubation with **53** shows a conclusive proof of covalent binding to EGFR with the release of 5FU *in vitro*. Study of the reaction of **53** with acetyl cysteine by NMR shows that 5FU is released in relevant time scales. Inhibition of EGFR phosphorylation in A431 cells shows that **53** engages EGFR in a cellular setting at appropriate concentrations. Together, these data provide evidence that the compound can both engage EGFR and release 5FU in tumor cells.

Our results suggest that obtaining the desired release profile requires appropriate electronics of the Michael acceptor system to enable both the initial addition and the release of the cytotoxic in a suitable time scale, without being so reactive as to be unstable. For 5FU, the acrylate ester system strikes the appropriate balance of reactivity. We would expect this to be transferrable to other conjugates with similar electronic properties to uracil but, in general, it is likely to depend on the codependent effects of the electron-donating ability of the conjugate that controls the initial addition and its leaving group ability that influences the rate of release. The observation of stable adducts without the release of 5FU for a number of analogues suggests that the elimination process is less facile and that protonation of the intermediate enolate species can be more favorable than the elimination.

**Figure 7:**
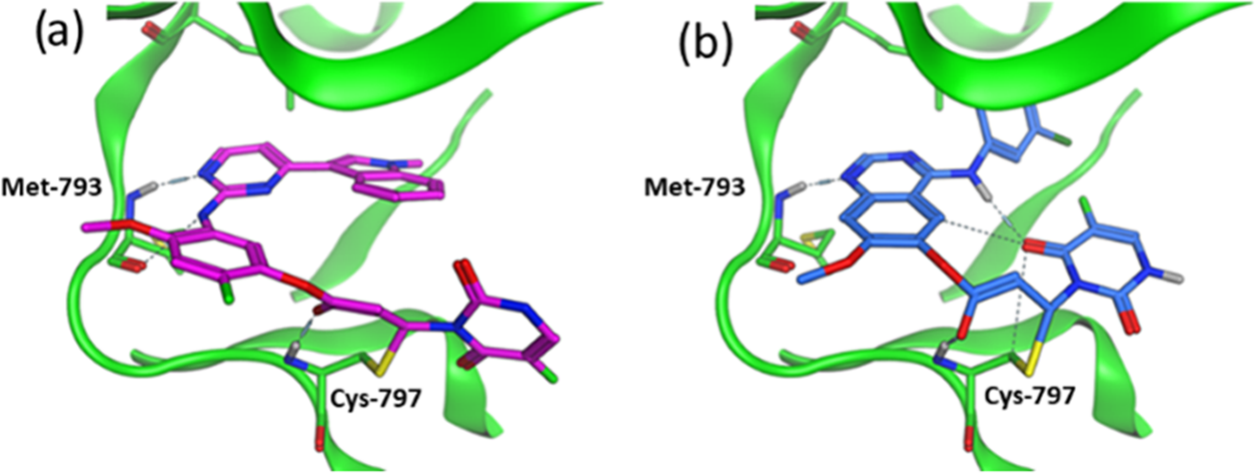
Molecular modeling of intermediate enolates arising from the conjugate addition for (a) pyrimidine **39** based on the pdb structure 6JX4; (b) quinazoline **53** based on the pdb structure 4G5J.

It is perhaps surprising that the desired elimination of 5FU occurred with quinazoline **53** but not with the analogous pyrimidine-derived acrylates **35** and **39**. We would postulate that the electronic properties of these compounds are similar and that the difference likely arises due to their bound geometries. To investigate this, we performed molecular modeling of the intermediate enolate adducts of **39** and **53** using QM-optimized ligand poses that were covalently docked into the appropriate crystal structure (pdb 6JX4 for **39** and 4G5J for **53**) (osimertinib template) before a final refinement of the structures. In order to preserve the optimized poses from the QM simulation, each ligand was restricted to move only 0.3 Å during the covalent docking process (Figure 7).

This showed that in both cases, the intermediate enolate has the potential to be stabilized by a hydrogen bond between the ester oxygen and the C797 backbone NH. In the case of anilinoquinazoline **53**, modeling showed the potential additional intramolecular hydrogen bond between the aniline NH and the uracil carbonyl. Hence, it is possible that the release profiles depend on the presence of secondary interactions that stabilize the transition state leading to elimination.

## Conclusions

Our results provide strong evidence that acrylate ester systems can be used as a delivery vector to release 5FU in cells via an addition–elimination reaction with EGFR. While the increases in growth inhibition observed with this combination are modest, 5FU is relatively weakly active as a cytotoxic agent. This approach may provide a general method for specific targeting agents, such as cytotoxic chemotherapy, to cancer cells via covalent binding to tumor-specific proteins. The use of more potent cytotoxic payloads would be expected to deliver greater efficacy.

## Experimental Section

### ^19^F NMR Release Studies

Acrylate-5FU conjugate (0.125 M) in *d*^6^-DMSO (0.25 mL) was prepared in an NMR tube. *N*-Acetyl cysteine methyl ester (1.9 mg, 0.011 mmol) or glutathione (3.4 mg, 0.011 mmol) in *d*^6^-DMSO was prepared. The cysteine/glutathione solution was added to the NMR tube, and the tube was inverted several times. The ^19^F NMR was taken immediately for *t* = 0 measurement. Further measurements were taken at *t* = 30 min and *t* = 60 min.

### Compound Media Stability

Compound **53** (0.760 mg, 1.51 μmol) was dissolved in DMSO (1.51 μL) and then added to DMEM (Sigma-Aldrich 5796) + 10% FCS media (1.50 mL) such that the final concentration was 1.0 μM. Liquid chromatography-mass spectrometry (LC-MS) analysis was carried out at *t* = 0, 1, 3, and 25 h.

### Protein Mass Spectrometry

Compounds were suspended in 10 mM in 100% DMSO and were added to 4 μM of EGFR to achieve the final compound concentration of 15 μM in 1% DMSO, in 25 mM Tris pH 8, 150 mM NaCl, 5% Glycerol before incubating at an ambient temperature for 4 hr time prior to analysis.

Following incubation, intact protein masses were determined using an Agilent 6530 Accurate Mass dual AJS/ESI Q-TOF instrument coupled to an Agilent 1260 Infinity II LC system. 1 μL of purified protein (∼1 mg/mL) was injected onto an MS Pac DS-10 Desalter cartridge ((Thermo Fisher Scientific), PN: 089170, 2.1 × 10 mm) for desalting and reversed phase separation at 70 °C. The mobile phase was 0.1% (v/v) formic acid in LC-MS grade water (A) and LC-MS grade acetonitrile (B) with separation performed over 7.5 min. Sample desalting was achieved at 30% B for 2 min at 1 mL/min before reducing the flow rate to 0.2 mL/min for 2 min. Protein elution was achieved at 100% B for 0.5 min and 1 mL/min before re-equilibration at 30% for 1 min. Proteins were detected in positive ion mode using electrospray ionization with a nebulizer pressure of 45 psig, a drying gas flow of 5 L/min, and a source gas temperature of 325 °C. A sheath gas temperature of 400 °C, a gas flow of 11 L/min, a capillary voltage of 3500 V, and a nozzle voltage of 2000 V were also used. Mass spectra were acquired using MassHunter Acquisition software (version B.08.00) over a mass range of 100–3000 *m*/*z* at a rate of 1 spectra/s and 1000 ms/spectrum in the standard mass range (3200 *m*/*z*) at 2 GHz. The instrument had been calibrated over the selected mass range prior to analysis.

### In Vitro TR-FRET Assay

Compounds (dissolved in 10 mM in DMSO) were dispensed into black low-volume 384-well assay plates (Corning) over a final concentration range of 100 000, 30 000, 10 000, 3000, 1000, 300, 100, 30, 10, and 3 nM using an Echo 550 (Labcyte). Positive control compound and DMSO as a negative control were dispensed into the first and last wells, respectively. Each well was backfilled with DMSO to a final volume of 200 nL, resulting in final assay DMSO concentrations of 1%. 19.8 μL of premixed solution containing the final assay concentration of CtermHisTag-mEGFR (wild type) (1.25 nM), probe (*N*-(4-(4-(3-((4-((3-chloro-4-fluorophenyl)amino)-7-methoxyquinazolin-6-yl)oxy)propyl)piperazin-1-yl)-4-oxobutyl)-3-(5,5-difluoro-7,9-dimethyl-5*H*-5l4,6l4-dipyrrolo[1,2-c:2′,1′-*f*][1,3,2]diazaborinin-3-yl)propenamide) (100 nM), and Tb-anti-His antibody 61HI2TLF (Cisbio Assay) (1:100 dilution) (https://uk.cisbio.eu/media/asset/c/i/cisbio_dd_pi_61hi2tlf-61hi2tla-61hi2tlb.pdf) in a buffer containing 20 mM Tris pH 7.5, 100 mM NaCl, 100 μg/mL bovine serum albumin, was added to each well and incubated with shaking at room temperature for 30 min. Plates were read using a PheraStar FS (BMG Labtech) at Ex 337 nm Ex 490/520 nm. The data were analyzed using Graphpad Prism/Dotmatics Studies Software. Assays were conducted in technical replicate and repeated as a biological duplicate.

### Cell Assays

#### Western Blotting

##### Lysate Preparation

Appropriately treated cells were lysed in Phosphosafe extraction buffer combined with cOmplete Protease Inhibitor Cocktail (Merck) prepared as per the manufacturer’s instructions.

##### Preparation of Samples

Samples were prepared in Eppendorf vials by diluting them to the required concentrations with phosphate-buffered saline (PBS), and the appropriate amount of 4X XT sample buffer was added. This solution was vortexed, centrifuged, then placed at 98 °C for 5 min, and then centrifuged to remove the residue. Migration was performed using a 4–20% Criterion TGX Precast Midi Protein Gel, 18 well, 30 μL (Cat. No. 5671094) placed in a Criterion running tank (Cat. No. 1656001) and also using PowerPac HC high-current power supply (Bio-Rad) electrodes. Nitrocellulose membrane used was an Amersham Protran Premium 0.45 Nitrocellulose membrane (Cat. No. 15269794) and Ponceau S Stain (Sigma-Aldrich Cat. No. p7170).

##### Preparation of 10 × Tris Glycine Sodium Dodecyl Sulfate (SDS) Running Buffer

Glycine (288 g, 3.84 mol), Trizma base (60.6 g, Sigma-Aldrich Cat. No. T1503), and SDS (20 g) (Sigma-Aldrich Cat. No. L3771) were added to deuterated water (1.60 L, 88.9 mol) and stirred until dissolved. The mixture was stored at room temperature.

##### Preparation of 1 × Tris Glycine SDS Running Buffer

To deuterated water (900 mL, 50 mol) was added 100 mL of the 10 × tris glycine SDS running buffer. It was stored at room temperature.

##### Preparation of the 1 × Transfer Buffer

To deuterated water (920 mL, 51 mol) were added 40 mL 25× transfer buffer (Invitrogen Cat. No. LC3675) and methanol (40 mL, 1.0 mol). It was stored at room temperature.

##### Preparation of 10 × TBS Solution

Tris-HCl (48.5 g) and NaCl (160 g, 2.70 mol) were added to deuterated water (1.6 L, 88.9 mol) and stirred until dissolved. The solution was then adjusted to pH 7.6 with NaOH, and then the volume was made up to 2 L with additional deuterated water. It was stored at room temperature.

##### Preparation of 1 × TBS/T (0.1 v/v) Solution

To 10 × TBS solution (100 mL) was diluted with deuterated water (900 mL, 50 mol) and Tween20 (1 mL, Sigma-Aldrich, Cat. No. P5927) was added and stirred until dissolved. It was stored at room temperature.

##### Preparation of Skimmed Milk (5% w/v)

To a falcon tube charged with dried skimmed milk (2.5 g, Marvel) was added 1 × TBS/T (0.1 v/v) solution (50 mL).

##### Preparation of Bovine Serum Albumin (BSA) Solution

To a falcon tube charged with bovine serum albumin (BSA) (2.5 g, Sigma-Aldrich) was added 1 × TBS/T (0.1 v/v) solution (50 mL).

##### Preparation of Primary Antibody Solution

Primary antibodies raised to EGFR (CST 4267) or pEGFR (CST 3777) were diluted (1:1000) in 5% BSA w/v. Loading control antibodies were prepared in 5% milk w/v with 1 × TBS/T (0.1 v/v) solution at a determined optimum concentration.

##### Preparation of Secondary Antibody Solution

Secondary HRP-conjugated antibodies were diluted in 5% milk with w/v in 1 × TBS/T (0.1 v/v) solution at a determined optimum concentration.

#### Detection

Proteins were detected using the Clarity ECL Western blotting substrate (Bio-Rad).

##### HTRF Method (A431)

Cells were plated at 20 000 cells per well in 96-well plates and placed at 37 °C with 5% CO_2_. Once adhered (after 24 h), cells were treated with compounds dissolved in DMSO at a final concentration of 0.1% DMSO in media. Compounds were diluted in media and then added to cells for 2.5 h in duplicate; 100 ng/mL of EGF (Thermo Fisher, PHG0311) was added to all compound-treated cells as well as control for 30 min. Compound and media were removed from the cells, and then 50 μL of HTRF lysis buffer was added (lysis buffer was diluted from 4× stock to 1× in deionized (DI) water, with 1% blocking agent also added). Cells were lysed on a plate shaker (2000 rpm) for 30 min at room temperature. pEGFR expression was monitored using the Cisbio Phospho-EGFR (Tyr1068) cellular kit (64EG1PEH). Total EGFR expression was monitored using the Cisbio Total EGFR cellular kit (64NG1PEH) as per the manufacturer’s instructions. Fluorescence emission was read at two different wavelengths (665 and 620 nm) on a PheraStar. Results were calculated as the ratio of pEGFR/total EGFR and then the percentage of 0 μM control.

#### Growth Inhibition in Adherent Cell Lines

A431 cells purchased from ECACC, Cat. 85090402, were plated on day 0 in a 96-well plate at a density known to allow for exponential growth over 72 h in DMEM (Sigma-Aldrich, Cat No. D5796) supplemented with 10% FBS (Gibco, Cat No. 10270-106). On day 1, the compounds were diluted to the required concentration in DMEM +10% FBS media, ensuring that the final DMSO concentration was 0.1% once added to cells. The cells were then incubated for 72 h at 37 °C with 5% CO_2_. Cells were then fixed by adding 50% (wt/vol) TCA to each well of the plate and left at 4 °C for 1 h. The plates were then washed thoroughly with water, 100 μL of 0.4% SRB solution was added to the wells, and left at room temperature for 30 min. The plates were rinsed with 1% AcOH and then left to air-dry in a drying cabinet for 1 h. Once dry, 100 μL of 10 mM tris pH 10.5 was added to each well and the plates were placed on a plate shaker for 10 min. The absorbance was read at 570 nm using a FluoStar Omega plate reader.

### Computational Methods

#### Pyrimidine System

An X-ray structure of 3 bound to EGFR wild-type protein was used as a start point for the evaluation of the potential positions on the molecule for the addition of a cytotoxic payload (PDB Code 4ZAU). For reasons that are not completely clear, the covalent bond between the inhibitor and protein was not formed in this X-ray structure. The X-ray structure was prepared for molecular modeling using the protein preparation wizard in Maestro (Schrodinger) to add hydrogens and confirm protonation and tautomer states of amino acids. Initially, the covalent bond was formed between the inhibitor and protein using the builder functionality and the local region was then optimized using the default forcefield (OPLS3). Visual inspection identified the position of the molecule to be most suitable for the addition of the payload and was manually built onto the molecule, followed by an additional round of optimization of the local atoms. The final model gives encouragement that the payload could be accommodated in this position during the formation of the covalent bond.

#### Quinazoline System

An X-ray structure of afatinib bound to EGFR wild-type protein was used as a start point for the evaluation of the potential positions on the molecule for the addition of a cytotoxic payload (PDB Code 4G5J). The X-ray structure was prepared for molecular modeling using the protein preparation wizard in Maestro (Schrodinger) to add hydrogens and confirm protonation and tautomer states of amino acids. The bound inhibitor was converted into the target molecule using the builder functionality and then allowed to optimize in the pocket. Visual inspection identified the position of the molecule to be most suitable for the addition of the payload and was manually built onto the molecule, followed by an additional round of optimization of the local atoms. The final model gives encouragement that the payload could be accommodated in this position during the formation of the covalent bond.

#### Intermediate Enolates

Modeling was performed using Maestro v2021-2. Ligand structures were initially prepared as the enolate form, wherein they were optimized in Jaguar using a B3-LYP functional and a 6-31G** basis set. Proteins were prepared using the Protein preparation Workflow in Maestro. The QM-optimized ligand poses were then covalently docked into 4G5J (AQZ template) and 6JX4 (osimertinib template) before a final refinement of the structures was performed using Prime. In order to preserve the optimized poses from the QM simulation, each ligand was restricted to move only 0.3 Å during the covalent docking process.

### General Chemical Methods

#### Chemicals and Solvents

All commercial reagents were purchased from reputable chemical companies. The chemicals were of the highest available purity. Unless otherwise stated, chemicals were used as supplied without further purification. Anhydrous solvents were obtained from either Sigma-Aldrich or Acros and were stored under nitrogen. Petrol refers to the fraction with a boiling point between 40 and 60 °C.

#### Chromatography

Thin-layer chromatography (TLC) utilized to monitor the reaction progress was conducted on plates precoated with silica gel (Merck 60F254). The eluent was as stated (where this consisted of more than one solvent; the ratio is stated as volume/volume), and visualization was either by short-wave (254 nm) ultraviolet light. “Flash” MPLC was carried out on a prepaced silica columns. Semipreparative HPLC was carried out on an Agilent instrument passing through a Waters XSelect column, employing a C_18_ 19 × 150 nm, 3.5 Å column (eluent: (acidic) 0.1% formic acid (aq)/MeCN, (basic) 0.1% NH_3_(aq)/MeCN), using a UV detector at 254 nm and a flow rate of 20 mL/min.

#### Analytical Techniques

Melting points were determined using a VWR Stuart SMP40 apparatus and are uncorrected. Optical rotations were recorded on a PolAAr 3001 Automatic Polarimeter (Optical Activity Ltd., Cambridgeshire, U.K.); units of [α]_D_ are given in 10^–1^ deg cm^2^ g^–1^. LC-MS was carried out on a Waters Acquity UPLC system with PDA and ELSD operating in positive and negative ion electrospray modes, employing an Acquity UPLC BEH C18, 1.7 mm, 2.1 mm × 50 mm column with 0.1% formic acid and water–acetonitrile (5–95%) for gradient elution. FTIR spectra were recorded on an Agilent Cary 630 FTIR spectrometer as a neat sample. Bond stretch frequencies are reported as br (broad), s (sharp), m (medium), or w (weak) based on their relative intensities. UV spectra were obtained using a U-2001 Hitachi spectrophotometer with the sample dissolved in ethanol. ^1^H, ^13^C, ^15^N, and ^19^F nuclear magnetic resonance (NMR) spectra were obtained as either CDCl_3_, CD_3_OD, or DMSO-*d*_6_ solutions and recorded at 500, 126, 700, and 470 MHz, respectively, on either a Bruker Avance III 500 or 700 spectrometer. Chemical shifts are quoted in parts per million (δ) referenced to the appropriate deuterated solvent employed. Multiplicities are indicated by s (singlet), d (doublet), t (triplet), q (quartet), p (pentet), m (multiplet), br (broad), or combinations thereof. Coupling constant values are given in Hz. Homonuclear and heteronuclear two-dimensional NMR experiments were used where appropriate to facilitate the assignment of chemical shifts. The numbering system used in the assignment of aromatic carbons and hydrogens is done according to the IUPAC nomenclature. All final compounds are >95% pure by HPLC analysis.

#### General Procedure A

To a solution of amine (1.0 equiv) in DMF (0.5 mmol/mL) were added acid (1.0 equiv), DMAP (0.4 equiv), and HATU (1.0 equiv) and allowed to stir at room temperature overnight. Once consumption of starting materials was observed, the reaction mixture was extracted into EtOAc, washed with water and brine, dried over MgSO_4_, and then concentrated *in vacuo*.

#### General Procedure B

To a solution of acid (1.0 equiv) in DCM (1.0 mmol/mL) at 0 °C was added a 1 M solution of DCC in DCM and stirred for 5 min before the addition of phenol (0.2 equiv) and DMAP (0.2 equiv). The reaction mixture was stirred for 4 h, with the resulting suspension filtered, and the filtrate was concentrated *in vacuo*.

#### General Procedure C

To a solution of 5FU (0.9 equiv) in DMF (1.0 mmol/mL) was added DABCO (0.5 equiv) and allowed to stir for 5 min before the addition of alkyne. The reaction mixture was then left to stir at 40 °C for 2 h. The solvent was then removed *in vacuo*.

##### 4-Fluorophenyl Propiolate, **7**

Synthesized following general procedure B. Purification was performed *via* flash column chromatography (eluent: 0–10% EtOAc in petroleum ether) to give the title compound (72 mg, 0.44 mmol, 49%) as a clear oil. *R_f_* = 0.40 (eluent: 10% EtOAc in petroleum ether); UV λ_max_ (nm) 263; IR: ν_max_/cm^–1^ 2926 (s, C–H alkyne), 2852 (s, C–H aromatic), 2111 (s, C≡C alkyne), 1736 (m, C=O ester), 1501 (m, C=C aromatic); ^1^H NMR (500 MHz, CDCl_3_): δ_H_ 7.11–6.95 (m, 4H), 3.02 (s, 1H); ^13^C NMR (126 MHz, CDCl_3_): δ_C_ 150.9, 144.9, 139.8, 122.7 (d, *J*_C-F_ = 8.60 Hz), 116.3 (d, *J*_C-F_ = 23.60 Hz), 77.6, 74.1; ^19^F NMR (470 MHz, CDCl_3_): δ_F_ −115.66; LRMS (ES^+^) *m*/*z* 165.1 [M + H]^+^.

##### 4-Chlorophenyl Propiolate, **8**

Synthesized following general procedure B. Purification was performed *via* flash column chromatography (eluent: 0–10% EtOAc in petroleum ether) to give the desired compound (95 mg, 0.53 mmol, 68%) as an orange oil. *R_f_* = 0.13 (eluent: 5% EtOAc in petroleum ether); UV λ_max_ (nm) 333, 277; IR: ν_max_/cm^–1^ 2926 (s, C–H alkyne), 2852 (m, C–H aromatic), 2111, (w, C≡C alkyne), 1736 (s, C=O ester), 1589 (s, C=C aromatic), ^1^H NMR (500 MHz, CDCl_3_): δ 7.29 (d, *J* = 8.83 Hz, 2H), 7.03 (d, *J* = 8.87 Hz, 2H), 3.05 (s, 1H); ^13^C NMR (126 MHz, CDCl_3_): δ_C_ 150.5, 148.2, 139.8, 129.7, 122.6, 77.2, 74.0; LRMS (ES^+^) **m*/*z** 181.1 [M(^35^Cl) + H]^+^ and 183.1 [M(^37^Cl) + H]^+^.

##### 4-Methoxyphenyl Propiolate, **9**

Synthesized following general procedure B. Purification was performed *via* flash column chromatography (eluent: 0–10% EtOAc in petroleum ether) to give the desired compound (120 mg, 0.68 mmol, 84%) as a clear oil. *R_f_* = 0.11 (eluent: 5% EtOAc in petroleum ether); UV λ_max_ (nm) 343, 264, 217; IR: ν_max_/cm^–1^ 3188 (w, C–H alkyne), 3049 (m, C–H aromatic), 2119, (w, C≡C alkyne), 1702 (s, C=O ester), 1504 (s, C=C aromatic); ^1^H NMR (500 MHz, CDCl_3_): δ_H_ 7.09 (d, *J* = 8.94 Hz, 2H, *H*^4^, *H*^6^), 6.92 (d, *J* = 8.82 Hz, 2H, *H*^1^, *H*^3^), 3.82 (s, 3H, *H*^13^), 3.09 (s, 1H, *H*^11^); ^13^C NMR (126 MHz, CDCl_3_): δ_C_ 157.8 (*C*^2^), 151.4 (*C*^5^), 143.3 (*C*^8^), 122.0 (*C*^4^, *C*^6^), 114.6 (*C*^1^, *C*^3^), 76.7 (*C*^11^), 74.3 (*C*^10^), 55.6 (*C*^13^); LRMS (ES^+^) **m*/*z** 177.2 [M + H]^+^.

##### 4-Cyanophenyl Propiolate, **10**

Synthesized following general procedure B. Purification *via* flash column chromatography (eluent: 0–10% EtOAc in petroleum ether) to give the title compound (72 mg, 0.44 mmol, 49%) as a white solid. *R_f_* = 0.10 (eluent: 10% EtOAc in petroleum ether); mp 150–152 °C; UV λ_max_ (nm) 230; IR: ν_max_/cm^–1^ 3235 (m, C–H alkyne), 3101 (w, C–H aromatic), 2236 (m, C≡N nitrile), 2086 (m, C≡C alkyne), 1724 (s, C=O ester), 1497 (m, C=C aromatic); ^1^H NMR (300 MHz, CDCl_3_): δ_H_ 7.75 (d, *J* = 8.83 Hz, 2H), 7.33 (d, *J* = 8.83 Hz, 2H), 3.17 (s, 1H); ^13^C NMR (126 MHz, CDCl_3_): δ_C_ 152.8, 149.8, 133.9, 122.5, 117.9, 110.7, 78.0, 73.6; LRMS (ES^+^) **m*/*z** 172.1 [M + H]^+^.

##### 4-Fluorophenyl (*E*)-3-(5-Fluoro-2,4-dioxo-3,4-dihydropyrimidin-1(2*H*)-yl)acrylate, **11**

Synthesized following general procedure C. Purification was performed *via* flash column chromatography (eluent: 0–10% MeOH in DCM) to give the title compound (22 mg, 0.08 mmol, 20%) as a white solid. *R_f_* = 0.41 (10% MeOH in DCM); mp 171–173 °C; UV λ_max_ (nm) 299, 243; IR: ν_max_/cm^–1^ 3192 (m, N–H urea), 3053 (m, C–H alkene), 2864 (m, C–H aromatic), 1705 (s, C=O ester), 1617 (s, C=O urea), 1505 (s, C=C aromatic); ^1^H NMR (300 MHz, DMSO-*d*_6_): δ_H_ 12.35 (s, 1H), 8.63 (d, *J* = 7.08 Hz, 1H), 8.17 (dd, *J* = 14.45 Hz, 1H), 7.27 (d, *J*_H-H_ = 9.00 Hz, *J*_H-F_ = 4.45 Hz, 2H), 7.25 (d, *J*_H-H_ = 9.00 Hz, *J*_H-F_ = 1.74 Hz, 2H), 6.52 (d, *J* = 14.45 Hz, 1H); ^13^C NMR (126 MHz, DMSO-*d*_6_): δ_C_ 165.2, 161.1, 159.1, 148.5, 140.9, 139.1, 124.1 (d, *J*_C-F_ = 8.70 Hz), 123.9, 116.6 (d, *J*_C-F_ = 23.50 Hz) 103.6; ^19^F NMR (282 MHz, DMSO-*d*_6_): δ_F_ −117.06 (*F*^11^), −163.32 (*F*^22^); LRMS (ES^+^) **m*/*z** 295.2 [M + H]^+^.

##### 4-Chlorophenyl (*E*)-3-(5-Fluoro-2,4-dioxo-3,4-dihydropyrimidin-1(2*H*)-yl)acrylate, **12**

Synthesized following general procedure C. Purification was performed *via* flash column chromatography (eluent: 0–10% MeOH in DCM) to give the desired compound (49 mg, 0.16 mmol, 36%) as a white solid. *R_f_* = 0.22 (5% MeOH in DCM); mp 173–175 °C; UV λ_max_ (nm) 292; IR: ν_max_/cm^–1^ 3322 (s, N–H), 2927 (s, C–H alkene), 2850 (s, C–H aromatic), 1703 (m, C=O ester), 1621 (C=O amide), 1568 (s, C=C aromatic); ^1^H NMR (300 MHz, DMSO-*d*_6_): δ_H_ 12.30 (s, 1H), 8.63 (d, *J*_H-F_ = 7.09 Hz, 1H), 8.18 (dd, *J* = 14.45 Hz, 1H), 7.50 (d, *J* = 8.90 Hz, 2H), 7.25 (d, *J* = 8.91 Hz, 2H), 6.52 (d, *J* = 14.45 Hz, 1H); ^13^C NMR (126 MHz, DMSO-*d*_6_): δ_C_ 165.0, 161.1, 157.1, 149.5, 141.1, 139.3, 130.5, 129.9, 124.3 (d, *J*_C-F_ = 28.98 Hz), 103.4; ^19^F NMR (470 MHz, DMSO-*d*_6_): δ_F_ −163.27; LRMS (ES^+^) **m*/*z** 308.0 [M + H]^+^.

##### 4-Methoxyphenyl (*E*)-3-(5-Fluoro-2,4-dioxo-3,4-dihydropyrimidin-1(2*H*)-yl)acrylate, **13**

Synthesized following general procedure C. Purification was performed *via* flash column chromatography (eluent: 0–10% MeOH in DCM) to give the desired compound (40 mg, 0.13 mmol, 29%) as a white solid. *R_f_* = 0.24 (5% MeOH in DCM); UV λ_max_ (nm) 298; mp 178–180 °C; IR: ν_max_/cm^–1^ 3188 (s, N–H), 3049 (s, C–H alkene), 2838 (s, C–H aromatic), 1702 (m, C=O ester), 1616 (C=O amide), 1504 (s, C=C aromatic); ^1^H NMR (500 MHz, DMSO-*d*_6_): δ_H_ 12.32 (s, 1H), 8.63 (d, *J*_H-F_ = 7.09 Hz, 1H), 8.16 (dd, *J* = 14.51 Hz, 1H), 7.11 (d, *J* = 9.06 Hz, 2H), 6.98 (d, *J* = 9.08 Hz, 2H), 6.51 (d, *J* = 14.50 Hz, 1H), 3.77 (s, 3H); ^13^C NMR (126 MHz, DMSO-*d*_6_): δ_C_ 165.5, 157.4, 148.5, 144.1, 140.9, 138.8, 124.2 (d, *J*_C-F_ = 36.40 Hz), 123.9, 123.1, 115.0, 104.0, 55.9; ^19^F NMR (470 MHz, DMSO-*d*_6_): δ_F_ −163.47; LRMS (ES^+^) **m*/*z** 306.2 [M + H]^+^.

##### 4-Cyanophenyl (*E*)-3-(5-Fluoro-2,4-dioxo-3,4-dihydropyrimidin-1(2*H*)-yl)acrylate, **14**

Synthesized following general procedure C. Purification was performed *via* flash column chromatography (eluent: 0–10% MeOH in DCM) to give the title compound (38 mg, 0.13 mmol, 22%) as a white solid. *R_f_* = 0.43 (10% MeOH in DCM); mp 180–182 °C; UV λ_max_ (nm) 300, 225; IR: ν_max_/cm^–1^ 3192 (m, N–H urea), 2925 (m, C–H alkene), 2845 (m, C–H aromatic), 2225 (s, C=N nitrile), 1720 (s, C=O ester), 1673 (s, C=O urea), 1501 (s, C=C aromatic); ^1^H NMR (300 MHz, DMSO-*d*_6_): δ_H_ 12.35 (s, 1H), 8.64 (d, *J*_H-F_ = 7.09 Hz, 1H), 8.21 (dd, *J* = 14.45, 1.8 Hz, 1H), 7.96 (d, *J* = 8.80 Hz, 2H), 7.46 (d, *J* = 8.80 Hz, 2H), 6.55 (dd, *J* = 14.45 Hz, 1H); ^13^C NMR (126 MHz, DMSO-*d*_6_): δ_C_ 164.6, 157.3, 154.2, 148.4, 141.0, 139.6, 134.5, 124.1, 123.8, 118.8, 109.3, 103.2; ^19^F NMR (470 MHz, DMSO-*d*_6_): δ_F_ −163.12; LRMS (ES^+^) **m*/*z** 300.1 [M + H]^+^.

##### Methyl (*E*)-3-(5-Fluoro-2,4-dioxo-3,4-dihydropyrimidin-1(2*H*)-yl)acrylate, **15**

To a solution of methyl propiolate (200 mg, 2.38 mmol) in DMF (5 mL) were added 5FU (464 mg, 3.57 mmol) and DABCO (ca. 5 mg, 0.04 mmol) at 0 °C and allowed to stir for 90 min. After this time, the reaction mixture was concentrated *in vacuo* to give a white solid. Purification was performed *via* flash column chromatography (eluent: 0–5% MeOH in DCM) to give the desired compound (166 mg, 0.78 mmol, 33%) as a white solid. *R_f_* = 0.32 (5% MeOH in DCM); mp 160–162 °C; UV λ_max_ (nm) 293, 239; IR: ν_max_/cm^–1^ 3041 (m, C–H alkene), 2837 (m, C–H alkane), 1689 (s, C=O); ^1^H NMR (500 MHz, DMSO-*d*_6_): δ_H_ 12.28 (s, 1H), 8.56 (d, *J*_H-F_ = 7.09 Hz, 1H), 8.02 (dd, *J* = 14.55 Hz, 1H), 6.31 (d, *J* = 14.55 Hz, 1H), 3.71 (s, 3H); ^13^C NMR (126 MHz, DMSO-*d*_6_): δ_C_ 166.8, 157.3, 148.4, 142.7, 140.8, 124.3 (d, *J*_C-F_ = 36.42 Hz), 104.7, 52.1; ^19^F NMR (470 MHz, DMSO-*d*_6_): δ_F_ −163.88; LRMS (ES^+^) **m*/*z** 215.1 [M + H]^+^.

##### (*E*)-3-(5-Fluoro-2,4-dioxo-3,4-dihydropyrimidin-1(2*H*)-yl)acrylic Acid, **16**

To a stirred solution of methyl (*E*)-3-(5-fluoro-2,4-dioxo-3,4-dihydropyrimidin-1(2*H*)-yl)acrylate (200 mg, 0.94 mmol) in THF (2.0 mL) was added 2 M aq. NaOH (1.0 mL, 2.0 mmol) and allowed to stir for 30 min at room temperature before the addition of 1 M aq HCl to adjust the pH of the reaction mixture to pH 3, then extracted with EtOAc (2 × 20 mL) and brine (20 mL), dried over MgSO_4_, and concentrated *in* vacuo to give the desired compound (186 mg, 0.93 mmol, 99%) as a white solid. *R_f_* = 0.15 (10% MeOH in DCM); mp 184–186 °C; UV λ_max_ (nm) 293, 239; IR: ν_max_/cm^–1^ 3306 (br, O–H), 3075 (m, C–H alkene), 1700 (s, C=O carboxylic acid), 1665 (s, C=O carbonate); ^1^H NMR (500 MHz, DMSO-*d*_6_): δ_H_ 12.48 (s, 1H), 12.25 (s, 1H), 8.55 (d, *J*_H-F_ = 6.98 Hz, 1H), 7.97 (dd, *J* = 14.51 Hz, 1H), 6.19 (d, *J* = 14.51 Hz, 1H); ^13^C NMR (126 MHz, DMSO-*d*_6_): δ_C_ 170.0, 159.6, 150.2, 137.0, 124.2, 124.1 (d, *J*_C-F_ = 36.40 Hz), 106.1; ^19^F NMR (470 MHz, DMSO-*d*_6_): δ_F_ −164.16; LRMS (ES^+^) **m*/*z** 201.1 [M + H]^+^.

##### 4-Nitrophenyl (*E*)-3-(5-Fluoro-2,4-dioxo-3,4-dihydropyrimidin-1(2*H*)-yl)acrylate, **17**

Synthesized following general procedure C. Purification was performed *via* flash column chromatography (eluent: 0–5% MeOH in DCM) to give the title compound (14 mg, 0.04 mmol, 30%) as a white solid. *R_f_* = 0.31 (10% MeOH in DCM); mp 210–212 °C; UV λ_max_ (nm) 293; IR: ν_max_/cm^–1^ 3322 (s, N–H), 2927 (s, C–H aromatic), 2849 (s, C–H alkyl), 1748 (w, C=O ester), 1623 (s, C=O carbamate), 1570 (s, C=C aromatic); ^1^H NMR (500 MHz, CDCl_3_): δ_H_ 8.42 (d, *J* = 14.50 Hz, 1H), 8.33 (d, *J* = 8.96 Hz, 2H), 7.63 (d, *J* = 5.29 Hz, 1H), 7.38 (d, *J* = 8.96 Hz, 2H), 6.06 (d, *J* = 14.56 Hz, 1H); ^13^C NMR (126 MHz, DMSO-*d*_6_): δ_C_ 164.7, 157.2, 155.7, 148.5, 145.7, 143.1, 139.7, 125.8, 124.1 (d, *J*_C-F_ = 17.64 Hz), 123.8, 103.2; ^19^F NMR (470 MHz, CDCl_3_): δ_F_ −158.37; LRMS (ES^+^) **m*/*z** 323.2 [M + H]^+^.

##### 1-(2-(2-Methoxyethoxy)ethoxy)-4-nitrobenzene, **18**

To a round-bottom flask charged with 4-nitrophenol (2.2 g, 15 mmol) in MeCN (80 mL) was added 2-(2-methoxyethoxy)ethyl-4-methylbenzenesulfonate (1.3 g, 4.2 mmol) and K_2_CO_3_ (8.7 g, 63 mmol) and stirred at 80 °C for 48 h. After this time, the reaction mixture was cooled to room temperature, concentrated *in vacuo*, extracted into EtOAc (100 mL), and washed with water (3 × 100 mL). The organics were combined, washed with brine (150 mL), dried over NaSO_4_, and concentrated *in vacuo* to give a brown oil. Purification was performed *via* column chromatography (0–100% EtOAc in petroleum ether) to give the desired compound (3.2 g, 13 mmol, 86%) as a pale brown oil. *R_f_* = 0.25 (100% DCM); UV λ_max_ 298, 226 nm; IR: ν_max_/cm^–1^ 2985 (m, C–H aromatic), 2835 (m, C–H alkyl), 1592 (s, N–O nitro), 1510 (s, C=C aromatic), 1342 (m, N–O nitro); ^1^H NMR (500 MHz, CDCl_3_): δ_H_ 8.22–8.17 (m, 2H), 7.00–6.95 (m, 2H), 4.25–4.21 (m, 2H), 3.92–3.87 (m, 2H), 3.75–3.70 (m, 2H), 3.60–3.56 (m, 2H), 3.39 (s, 3H); ^13^C NMR (126 MHz, CDCl_3_): δ_C_ 163.8, 141.6, 125.9, 114.6, 71.9, 70.6,, 69.3, 68.2, 59.1; LRMS (ES^+^) **m*/*z** 241.1.

##### 4-(2-(2-Methoxyethoxy)ethoxy)aniline, **19**

To a round-bottom flask, 1-(2-(2-methoxyethoxy)ethoxy)-4-nitrobenzene (3.20 g, 13.0 mmol) was charged in MeOH (80 mL) and DCM (20 mL) and reacted using a H-cube apparatus using 10% Pd/C CatCart at 40 °C and 1 mL/min in full H_2_ mode for 21 h. The reaction mixture was concentrated *in vacuo* to give the desired compound (2.81 g, 12.9 mmol, 99%) as a brown oil. The compound was carried through without any further purification. *R_f_* = 0.14 (10% MeOH in DCM); UV λ_max_ 300 nm; IR: ν_max_/cm^–1^ 3432 (br N–H), 2900 (m, C–H aromatic), 2875 (C–H alkyl), 1509 (s, C=C aromatic); ^1^H NMR (500 MHz, CD_3_Cl): δ_H_ 6.78 (d, *J* = 8.78 Hz, 2H), 6.66 (d, *J* = 8.79 Hz, 2H), 4.09 (t, *J* = 5.77, 4.18 Hz, 2H*)*, 3.85–3.83 (m, 2H), 3.74–3.73 (m, 2H), 3.61–3.59 (m, 2H), 3.41 (s, 3H); ^13^C NMR (126 MHz, CDCl_3_): δ_C_ 152.0, 139.9, 116.5, 115.9, 72.0, 70.7, 70.0, 68.1, 59.1; LRMS (ES^+^) *m*/*z* 212.0 [M + H]^+^.

##### *N-*(4-(2-(2-Methoxyethoxy)ethoxy)phenyl)propiolamide, **20**

Synthesized following general procedure A. Purification was performed *via* column chromatography (eluent: 0–10% MeOH in DCM) to give the title compound (80 mg, 0.33 mmol, 70%) as a brown oil. *R_f_* = 0.40 (10% MeOH in DCM); UV λ_max_ (nm) 310, 259; IR: ν_max_/cm^–1^ 3441 (br N–H), 2905 (m, C–H aromatic), 2878 (C–H alkyl), 2108 (w, C≡C), 1509 (s, C=C aromatic); ^1^H NMR (500 MHz, CDCl_3_): δ_H_ 7.49 (d, *J* = 9.30 Hz, 2H), 6.90 (d, *J* = 9.37 Hz, 2H), 4.08 (m, 2H*)*, 3.81–3.79 (m, 2H), 3.70 (s, 1H) 3.69–3.67 (m, 2H), 3.57–3.55 (m, 2H), 3.36 (s, 3H); ^13^C NMR (126 MHz, CDCl_3_): δ_C_ 165.8, 155.9, 149.8, 121.8, 118.2, 77.9, 73.8, 71.9, 70.7, 69.7, 67.6, 59.0; LRMS (ES^+^) *m*/*z* 264.2 [M + H]^+^.

##### (*E*)-3-(5-Fluoro-2,4-dioxo-3,4-dihydropyrimidin-1(2*H*)-yl)-N-(4-(2-(2-methoxyethoxy)ethoxy)phenyl)acrylamide, **21**

Synthesized following general procedure C. Purification was performed *via* reverse phase column chromatography (C_18_ silica; 5–100%, MeOH/H_2_O + 0.1% HCOOH) and normal phase column chromatography (5–15% MeOH in DCM) to give the title compound (12 mg, 0.03 mmol, 10%) as a white solid. *R_f_* = 0.62 (MeOH/H_2_O (1:2) (C_18_ reverse phase plate)); mp 240–242 °C; UV λ_max_ (nm) 310, 264, 205; IR: ν_max_/cm^–1^ 3445 (br, N–H), 3074 (m, C–H aromatic), 2919 (C–H alkene), 2850 (C–H alkyl), 1716 (s, C=O), 1660 (s, C=O), 1621 (s, C=O), 1511 (s, C=C aromatic); ^1^H NMR (500 MHz, DMSO-*d*_6_): δ_H_ 8.22 (dd, *J*_H-F_ = 6.44 Hz, *J*_H-H_ = 1.20 Hz, 1H), 7.88 (d, *J* = 14.13 Hz, 1H), 7.50 (d, *J* = 9.11 Hz, 2H), 6.88 (d, *J* = 8.95 Hz, 2H), 6.24 (d, *J* = 14.13 Hz, 1H), 4.57 (t, *J* = 4.61 Hz, 2H*)*, 4.29–4.27 (m, 2H), 4.16–4.14 (m, 2H), 4.03–4.01 (m, 2H), 3.81 (s, 3H); ^13^C NMR (126 MHz, DMSO-*d*_6_): δ_C_ 191.9, 167.7, 159.7, 152.3, 146.2, 138.4 (d, *J*_C-F_ = 21.35 Hz), 135.6, 127.7 (*C*^4^), 118.3 (*C*^19^), 112.7 (*C*^18^), 75.5 (*C*^8^), 74.1 (*C*^9^), 73.4 (*C*^11^), 71.4, 61.6; ^19^F NMR (470 MHz, DMSO-*d*_6_): δ_F_ −161.75; LRMS (ES^+^) *m*/*z* 394.1 [M + H]^+^.

##### 1-Phenyl-4-(trimethylsilyl)but-3-yn-2-one, **22**

To a round-bottom flask under an atmosphere of N_2_ containing a solution of phenylacetyl chloride (0.68 mL, 5.0 mmol) in DCM (5 mL) were added bis(trimethylsilyl)acetylene (1.1 mL, 5.0 mmol) and AlCl_3_ (0.65 g) and allowed to stir at room temperature for 18 h. 1 M HCl (1.5 mL) was then added, extracted with DCM (20 mL), and washed with H_2_O (3 × 30 mL), sat. aq. NaHCO_3_ (30 mL), dried over MgSO_4_, and concentrated *in vacuo* to give a viscous black oil. Purification was performed *via* column chromatography (eluent: 0–20% DCM in petroleum ether) to give the title compound (0.50 g, 2.3 mmol, 46%) as a yellow oil. *R_f_* = 0.70 (10% EtOAc in petroleum ether); UV λ_max_ = 252, 206 nm; IR: ν_max_/cm^–1^ 2961 (m, C–H aromatic), 2830 (m, C–H alkyl), 2150 (w, C≡C), 1672 (s, C=O), 1495 (s, C=C aromatic); ^1^H NMR (500 MHz, CDCl_3_): δ_H_ 7.32–7.23 (m, 5H), 3.83 (s, 2H), 0.21 (s, 9*H*); ^13^C NMR (126 MHz, CD_3_OD): δ_C_ 196.6, 163.1, 139.9, 134.7, 130.6, 130.0, 128.4, 51.5, 0.0; LRMS (ES^+^) *m*/*z* 217.2 [M + H]^+^.

##### 1-Phenylbut-3-yn-2-one, **23**

To a round-bottom flask under an atmosphere of N_2_ containing a solution of 1-phenyl-4-(trimethylsilyl)but-3-yn-2-one (0.27 g, 1.30 mmol) in THF (20 mL), MeOH (0.41 mL, 10 mmol) and TBAF (0.41 mL, 0.41 mmol, 1 M in THF) were added dropwise at −78 °C and allowed to stir at that temperature for 1 h. 1 M HCl (1.5 mL) was then added, and the mixture was poured into ice (ca. 60 g) and warmed to room temperature, extracted in EtOAc (2 × 80 mL), and washed with H_2_O (3 × 100 mL) and brine (100 mL), dried over MgSO_4_, and concentrated *in vacuo* to give a yellow oil. Purification was performed *via* column chromatography (eluent: 0–10% DCM in petroleum ether) to give the title compound as a yellow oil (0.17 g, 1.2 mmol, 96%). *R_f_* = 0.71 (10% DCM in petroleum ether); UV λ_max_ (nm) 264, 205; IR: ν_max_/cm^–1^ 2956 (m, C–H aromatic), 2112 (w, C≡C), 1685 (s, C=O), 1510 (s, C=C aromatic); ^1^H NMR (500 MHz, CDCl_3_): δ_H_ 7.18–7.06 (m, 5H), 3.68 (s, 2H), 3.04 (s, 1H); ^13^C NMR (126 MHz, CD_3_OD): δ_C_ 193.8, 145.6, 131.9, 127.1, 125.6, 103.1, 101.4, 51.5; LRMS (ES^+^) *m*/*z* 145.1 [M + H]^+^.

##### (*E*)-5-Fluoro-1-(3-oxo-4-phenylbut-1-en-1-yl)pyrimidine-2,4(1*H*,3*H*)-dione, **24**

Synthesized following general procedure C. Purification was performed *via* column chromatography (eluent: 2–10% MeOH in DCM) to give the title compound (5 mg, 1.8 × 10^–2^ mmol, 13%) as a yellow solid. *R_f_* = 0.73 (10% MeOH in DCM); mp 241–243 °C; UV λ_max_ (nm) 301, 224; IR: ν_max_/cm^–1^ 3445 (br, N–H), 3074 (m, C–H aromatic), 2912 (m, C–H alkene), 2850 (m, C–H alkyl), 1716 (s, amide C=O), 1690 (s, ketone C=O), 1511 (C=C aromatic); ^1^H NMR (500 MHz, DMSO-*d*_6_): δ_H_ 12.29 (br s, 1H), 8.56 (d, *J*_H-F_ = 7.36 Hz, 1H), 8.00 (dd, *J* = 14.70, 2.09 Hz, 1H), 7.34–7.23 (m, 5H), 6.71 (d, *J* = 14.70 Hz, 1H), 3.91 (s, 2H); ^13^C NMR (126 MHz, DMSO-*d*_6_): δ_C_ 196.5, 157.3 (d, *J*_C-F_ = 26.87 Hz), 148.6, 142.7, 140.8, 136.3, 130.0, 128.9, 127.1, 124.0 (d, *J*_C-F_ = 36.41 Hz), 112.7, 48.5; ^19^F NMR (470 MHz, DMSO-*d*_6_): δ_F_ −163.75; LRMS (ES^+^) *m*/*z* 275.1 [M + H]^+^.

##### (*E*)-4-(2-Butoxyvinyl)-2,5-dichloropyrimidine, **25**

To a round-bottom flask under an atmosphere of N_2_ charged with 2,4,5-trichloropyrimidine (2.00 g, 11.0 mmol) was added PEG-200 (10 mL), followed by Et_3_N (1.60 mL, 11.0 mmol) and butyl vinyl ether (1.5 mL, 11 mmol), and stirred for 10 min before being added to 80 °C. Pd(OAc)_2_ (80 mg, 0.32 mmol) was then added, and the reaction mixture was left to stir for 1 h before being allowed to cool to room temperature. The reaction mixture was then extracted into Et_2_O (3 × 30 mL), the combined organics were washed with H_2_O (3 × 50 mL), dried over MgSO_4_, and concentrated *in vacuo* to give a dark red oil. Purification was performed *via* column chromatography (eluent: 0–30% DCM in petroleum ether) to give the desired compound (0.83 g, 3.4 mmol, 31%) as a clear oil. *R_f_* = 0.52 (100% DCM); UV λ_max_ (nm) 358, 288, 214; IR: ν_max_/cm^–1^ 2959 (m, C–H, aromatic), 2873 (m, C–H alkyl), 1638 (s, C=O), 1610 (s, C=C alkene), 1545 (s, C=C aromatic); ^1^H NMR (500 MHz, CDCl_3_): δ_H_ 8.32 (s, 1H), 8.07 (d, *J* = 12.04 Hz, 1H), 6.09 (d, *J* = 11.98 Hz, 1H), 4.03 (t, *J* = 6.49 Hz, 2H), 1.73 (m, 2H), 1.50–1.39 (m, 2H), 0.97 (m, 3H); ^13^C NMR (126 MHz, CDCl_3_): δ_C_ 163.1, 161.3, 158.0, 157.5, 124.8, 98.7, 72.3, 31.2, 18, 13.7; LRMS (ES^+^) **m*/*z** 247.0 [M(^35^Cl_2_) + H]^+^, 249.0 [M(^35^Cl + ^37^Cl) + H]^+^ and 251.0 [M(^37^Cl_2_) + H]^+^.

##### 3-(2,5-Dichloropyrimidin-4-yl)pyrazolo[1,5-*a*]pyridine, **26**

To a round-bottom flask under an atmosphere of N_2_ charged with (*E*)-4-(2-butoxyvinyl)-2,5-dichloropyrimidine (500 mg, 2.02 mmol) in DMA (20 mL) were added 1-aminopyridinium iodide (466 mg, 2.10 mmol) and K_2_CO_3_ (892 mg, 6.46 mmol), heated to 110 °C, and allowed to stir for 18 h. After being allowed to cool to room temperature, the reaction mixture was solidified. This was then diluted with EtOAc (50 mL) and a small quantity of MeOH (2 mL), washed with water (40 mL) and brine (100 mL), dried over MgSO_4_, and concentrated *in vacuo* to give an orange oil. Purification was performed *via* column chromatography (eluent: 100% DCM) to give the desired compound as an off-white solid (420 mg, 1.58 mmol, 78%). *R_f_* = 0.41 (50% EtOAc in petroleum ether); mp 175–177 °C; UV λ_max_ (nm) 353, 293, 220; IR: ν_max_/cm^–1^ 2945 (m, C–H aromatic), 2089 (m br, N–N), 1590 (s, C=C aromatic); ^1^H NMR (500 MHz, CDCl_3_): δ_H_ 9.06 (s, 1H), 8.75 (dd, *J* = 9.00, 1.22 Hz, 1H), 8.61 (dd, *J* = 6.92, 1.12 Hz, 1H), 8.51 (s, 1H), 7.54 (ddd, *J* = 9.00, 6.88, 1.22 Hz, 1H), 7.08 (dd, *J* = 6.88, 1.41 Hz, 1H); ^13^C NMR (126 MHz, CDCl_3_): δ_C_ 165.3, 157.6, 156.6, 144.0, 143.3, 139.9, 135.0, 128.9, 126.3, 125.7, 121.2; LRMS (ES^+^) *m*/*z* 264.1 [M(^35^Cl_2_) + H]^+^, 266.1 [M(^35^Cl + ^37^Cl) + H]^+^ and 268.1 [M(^37^Cl_2_) + H]^+^.

##### 5-Chloro-*N*-(2-methoxy-5-nitrophenyl)-4-(pyrazolo[1,5-*a*]pyridin-3-yl)pyrimidin-2-amine, **27**

To a stirred solution of 2-methoxy-5-nitroaniline (200 mg, 0.75 mmol) and 3-(2,5-dichloropyrimidin-4-yl)pyrazolo[1,5-*a*]pyridine (126 mg, 0.75 mmol) in 2-pentanol (10 mL) was added *p*-TSA (150 mg, 0.85 mmol) and heated to 110 °C under an atmosphere of N_2_. This was left to stir for 5 days, after which time the resulting precipitate was filtered, washed with 2-pentanol (ca. 10 mL) and MeCN (ca. 10 mL), and left to dry *in vacuo* to give a brown solid (210 mg, 0.53 mmol, 70%) as the desired compound. *R_f_* = 0.70 (100% DCM); mp 288–290 °C; UV λ_max_ (nm) 298; IR: ν_max_/cm^–1^ 3413 (m, N–H), 2846 (w, C–H aromatic), 2758 (w, C–H alkyl), 1554 (s, C=C aromatic), 1528 (s, N=O), 1343 (s, N–O); ^1^H NMR (500 MHz, DMSO-*d*_6_): δ_H_ 8.99 (s, 1H), 8.92 (s, 1H), 8.89 (d, *J* = 8.89 Hz, 1H), 8.87 (d, *J* = 2.84 Hz, 1H), 8.59 (s, 1H), 8.55 (d, *J* = 8.84 Hz, 1H), 8.08 (dd, *J* = 9.00, 2.89 Hz, 1H), 7.43 (d, *J* = 8.89 Hz, 1H), 7.33 (d, *J* = 9.09 Hz, 1H), 7.17 (d, *J* = 8.87 Hz, 1H), 4.01 (s, 3H); LRMS (ES^+^) **m*/*z** 396.1 [M(^35^Cl) + H]^+^ and 398.1 [M(^37^Cl) + H]^+^.

##### *N*^1^-(5-Chloro-4-(pyrazolo[1,5-*α*]pyridin-3-yl)pyrimidin-2-yl)-6-methoxybenzene-1,3-diamine, **28**

To a stirred solution of 5-chloro-*N*-(2-methoxy-5-nitrophenyl)-4-(pyrazolo[1,5-*a*]pyridin-3-yl)pyrimidin-2-amine (117 mg, 0.30 mmol) in MeOH/H_2_O (9:1, 10 mL) were added NH_4_Cl (147 mg, 2.7 mmol) and Zn powder (190 mg, 2.9 mmol), then heated to 70 °C, and allowed to stir at this temperature for 3.5 h. After this time, the reaction mixture was filtered, with the solids washed with DCM (ca. 20 mL) and MeOH (ca. 10 mL), with the resulting filtrate concentrated *in vacuo* to give a dark green solid (84 mg, 0.23 mmol, 78%) as the title compound. *R_f_* = 0.14 (100% DCM); mp 183–185 °C; UV λ_max_ (nm) 334, 293; IR: ν_max_/cm^–1^ 3447 (br, Ar(N–H)Ar’), 3356 (m, N–H), 3104 (w, C–H aromatic), 2995 (w, C–H alkyl), 1560 (s, C=C aromatic); ^1^H NMR (500 MHz, DMSO-*d*_6_): δ_H_ 8.94 (s, 1H), 8.64 (dd, *J* = 6.94, 1.09 Hz, 1H), 8.55 (dd, *J* = 9.01, 1.27 Hz, 1H), 8.36 (s, 1H), 7.64 (d, *J* = 2.72 Hz, 1H), 7.46 (ddd, *J* = 9.05, 6.81, 1.30 Hz, 1H), 7.09 (dd, *J* = 6.85, 1.30 Hz, 1H), 6.85 (d, *J* = 8.57 Hz, 1H), 6.51 (dd, *J* = 8.54, 2.71 Hz, 1H), 3.35 (s, 3H); ^13^C NMR (126 MHz, DMSO-*d*_6_): δ_C_ 159.0, 157.8, 155.5, 143.3, 142.4, 139.4, 129.4, 128.5, 127.1, 125.4, 120.8, 114.4, 114.3, 112.8, 111.2, 110.0, 107.0, 56.1; LRMS (ES^+^) **m*/*z** 367.0 [M(^35^Cl) + H]^+^ and 369.1 [M(^37^Cl) + H]^+^.

##### *N*-(3-((5-Chloro-4-(pyrazolo[1,5-*α*]pyridin-3-yl)pyrimidin-2-yl)amino)-4-methoxyphenyl)propiolamide, **29**

Synthesized following general procedure A. Purification was performed *via* flash column chromatography (100% DCM over 10 min and then 0–5% MeOH in DCM over 15 min) to give a yellow oil (24 mg, 57 μmol, 62%) as the desired compound. *R_f_* = 0.23 (100% DCM); UV λ_max_ (nm) 304, 276; IR: ν_max_/cm^–1^ 3409 (br, N–H), 3228 (w, C–H aromatic), 2923 (w, C–H alkyl), 2103 (m, C≡C), 1750 (w, C=O amide), 1555 (m, C=C aromatic) 1220 (s, C–O); ^1^H NMR (500 MHz, DMSO-*d*_6_): δ_H_ 10.69 (s, 1H), 8.97 (s, 1H), 8.86 (dd, *J* = 6.96, 1.12 Hz, 1H), 8.73 (s, 1H), 8.44 (s, 1H), 8.38 (d, *J* = 8.93 Hz, 1H), 7.91 (d, *J* = 2.65 Hz, 1H), 7.46 (dd, *J* = 8.88, 2.57 Hz, 1H), 7.34 (dd, *J* = 7.89 Hz, 1H), 7.14 (dd, *J* = 6.88, 1.46 Hz, 1H), 7.08 (d, *J* = 8.93 Hz, 1H), 4.35 (s, 1H), 3.78 (s, 3H); ^13^C NMR (126 MHz, DMSO-*d*_6_): δ_C_ 159.3, 158.3, 157.7, 149.7, 149.4, 143.8, 139.8, 131.5, 130.0, 128.4, 127.3, 121.8, 117.7, 117.2, 115.2, 114.9, 112.0, 107.5, 98.7, 98.4, 56.2; LRMS (ES^+^) **m*/*z** 419.3 [M(^35^Cl) + H]^+^ and 421.1 [M(^37^Cl) + H]^+^.

##### (*E*)-*N*-(3-((5-Chloro-4-(pyrazolo[1,5-*a*]pyridin-3-yl)pyrimidin-2-yl)amino)-4-methoxyphenyl)-3-(5-fluoro-2,4-dioxo-3,4-dihydropyrimidin-1(2*H*)-yl)acrylamide, **30**

Synthesized following general procedure C. Purification was performed *via* flash column chromatography (0–3% over 10 min and then 3–10% over 10 min MeOH in DCM) to give the desired product (22 mg, 40 μmol, 17%) as a beige solid. *R_f_* = 0.14 (10% MeOH in DCM); mp 256–258 °C; UV λ_max_ (nm) 276; IR: ν_max_/cm^–1^ 3084 (w, C–H aromatic), 1627 (m, C=O amide), 1516 (m, C=C aromatic); ^1^H NMR (500 MHz, DMSO-*d*_6_): δ_H_ 8.97 (s, 1H), 8.83 (d, *J* = 8.60 Hz, 1H), 8.71 (s, 1H), 8.44 (d, *J* = 2.55, 1H), 8.10 (d, *J* = 15.33 Hz, 1H), 7.93 (d, *J* = 6.84 Hz, 1H), 7.56 (m, 1H), 7.33 (dd, *J* = 8.60, 7.11 Hz, 1H), 7.11 (d, *J* = 7.11 Hz, 1H), 7.06 (d, *J* = 9.30 Hz, 1H), 6.01 (d, *J* = 15.33 Hz, 1H), 3.77 (s, 3H); ^13^C NMR (126 MHz, DMSO-*d*_6_): δ_C_ 172.7, 169.5, 166.9, 164.9, 157.9, 156.3, 152.2, 149.3, 144.1, 143.1, 142.5, 141.9, 131.1, 125.9, 125.1, 124.7, 123.9, 123.3, 122.9 (d, *J*_C-F_ = 21.58 Hz), 120.3, 120.0, 119.7, 117.2, 110.6, 60.7; ^19^F NMR (470 MHz, DMSO-*d*_6_): δ_F_ −171.48 LRMS (ES^+^) **m*/*z** 549.3 [M(^35^Cl) + H]^+^ and 551.4 [M(^37^Cl) + H]^+^.

##### 2-Fluoro-4-methoxyphenyl acetate, **31**

To a round-bottomed flask charged with 2-fluoro-4-methoxyphenol (1.0 g, 7.0 mmol) in DCM (3 mL) was added acetyl chloride (0.8 mL, 11.3 mmol) followed by the dropwise addition of Et_3_N (1.5 mL, 12.6 mmol) and allowed to stir at room temperature for 18 h. After this time, the reaction mixture was extracted into EtOAc (2 × 40 mL), the combined organics were washed with brine (100 mL), dried over MgSO_4_, and concentrated *in vacuo* to give a brown oil. Purification was performed *via* flash column chromatography (eluent: 0–10% EtOAc in petroleum ether) to give the desired product (1.1 g, 5.9 mmol, 85%) as a clear oil. *R_f_* = 0.25 (10% EtOAc in petroleum ether); mp 94–97 °C; UV λ_max_ (nm) 273; IR: ν_max_/cm^–1^ 2940 (w, C–H aromatic), 2840 (w, C–H alkyl), 1764 (s, C=O ester), 1508 (s, C=C aromatic); ^1^H NMR (500 MHz, CDCl_3_): δ_H_ 7.02 (dd, *J* = 8.81 Hz, 1H), 6.72 (dd, *J*_H-F_ = 11.78 Hz, *J*_H-H_ = 2.88 Hz, 1H), 6.66 (ddd, *J*_H-H_ = 8.90, 2.95 Hz, *J*_H-F_ = 1.43 Hz, 1H), 3.78 (s, 3H), 2.31 (s, 3H); ^13^C NMR (126 MHz, CDCl_3_): δ_C_ 168.8, 158.3, 153.3, 131.6, 123.7, 109.6 (d, *J*_C-F_ = 22.20 Hz), 102.9, 55.8*H*HH, 20.5; ^19^F NMR (470 MHz, CDCl_3_): δ_F_ −126.19; LRMS (ES^+^) **m*/*z** 185.1 [M + H]^+^.

##### 2-Fluoro-4-methoxy-5-nitrophenyl acetate, **32**

To a round-bottomed flask charged with 2-fluoro-4-methoxyphenyl acetate (1.1 g, 6.0 mmol) in acetic acid (15 mL) at 0 °C was added 95–97% of H_2_SO_4_ (4.0 mL, 7.1 mmol) followed by the dropwise addition of fuming HNO_3_ (3.0 mL, 7.1 mmol) and stirred for 30 min. After this time, the reaction mixture was poured over ice (ca. 10 g) and stirred for a further 1 h, forming a white precipitate, which was then filtered to reveal the intended product as a white solid (1.2 g, 5.4 mmol, 91%). *R_f_* = 0.32 (10% MeOH in DCM); mp 192–195 °C; UV λ_max_ = 286 nm; IR: ν_max_/cm^–1^ 3072 (w, C–H alkane), 2866 (w, C–H aromatic), 1763 (s, C=O ester) 1588 (m, C=C aromatic); ^1^H NMR (500 MHz, CDCl_3_): δ_H_ 7.83 (d, *J*_H-F_ = 7.82 Hz, 1H), 6.91 (d, *J*_H-F_ = 11.19 Hz, 1H), 3.97 (s, 3H), 2.35 (s, 3H); ^13^C NMR (126 MHz, CDCl_3_): δ_C_ 168.0, 158.5, 156.4, 152.8, 130.5, 122.0, 102.5 (d, *J*_C-F_ = 24.31 Hz), 57.2, 20.3; ^19^F NMR (470 MHz, CDCl_3_): δ_F_ −114.41; LRMS (ES^+^) **m*/*z** 228.1 [M + H]^+^.

##### 5-Amino-2-fluoro-4-methoxyphenyl acetate, **33**

To a stirred solution of 2-fluoro-4-methoxy-5-nitrophenyl acetate (600 mg, 2.6 mmol) in MeOH (20 mL) under an atmosphere of H_2_ was added 10% Pd on carbon (250 mg, 0.23 mmol) and allowed to stir for 24 h at room temperature. After this time, the reaction mixture was passed through a pad of celite and washed with MeOH (ca. 100 mL) and DCM (50 mL), with the resulting filtrate concentrated *in vacuo* to give the desired aniline (516 mg, 2.6 mmol, 99%) as a black oil. *R_f_* = 0.13 (10% MeOH in DCM); UV λ_max_ (nm) 400, 358, 295; IR: *v*_max_/cm^–1^ 3362 (s, N–H), 2940 (w, C–H aromatic), 2841 (w, C–H alkyl), 1749 (s, C=O ester), 1512 (s, C=C aromatic); ^1^H NMR (500 MHz, CDCl_3_): δ_H_ 6.63 (d, *J*_H-F_ = 11.31 Hz, 1H), 6.45 (d, *J*_H-F_ = 7.54 Hz, 1H), 3.82 (s, 3H), 3.68 (s, 2H), 2.29 (s, 3H); ^13^C NMR (126 MHz, CDCl_3_): δ_C_ 168.9, 147.5, 145.6, 145.1, 130.8, 109.0, 99.9 (d, *J*_C-F_ = 23.55), 56.0, 20.5; ^19^F NMR (470 MHz, CDCl_3_): δ_F_ −139.83; LRMS (ES^+^) **m*/*z** 200.0 [M + H]^+^.

##### 2-Fluoro-4-methoxy-5-((4-(1-methyl-1*H*-indol-3-yl)pyrimidin-2-yl)amino)phenol, **34**

To a stirred solution of 5-amino-2-fluoro-4-methoxyphenyl acetate (182 mg, 0.91 mmol) in 2-pentanol (5 mL) was added 3-(2-chloropyrimidin-4-yl)-1-methyl-1*H*-indole (140 mg, 0.91 mmol) followed by *p*-TSA (207 mg, 1.10 mmol) and then allowed to stir at 110 °C for 18 h. After this time, the reaction mixture was concentrated *in vacuo* to give a black-green oil. Purification was performed *via* flash column chromatography (eluent: 0–10% MeOH in DCM) to give the desired compound (148 mg, 0.41 mmol, 45%) as a green solid. *R_f_* = 0.32 (5% MeOH in DCM); mp 198–200 °C; UV λ_max_ = 371, 264, 213 nm; IR: ν_max_/cm^–1^ 3056 (w, C–H alkane), 2921 (w, C–H aromatic), 1576 (m, C=C aromatic); ^1^H NMR (500 MHz, CDCl_3_): δ_H_ 8.46 (d, *J*_H-F_ = 9.74 Hz, 1H), 8.40 (dd, *J* = 6.72, 2.02 Hz, 1H), 8.35 (d, *J* = 5.46 Hz, 1H), 7.85 (s, 1H), 7.56 (s, 1H), 7.39 (d, *J* = 7.98 Hz, 1H), 7.36–7.28 (m, 2H), 7.08 (d, *J* = 5.40 Hz, 1H), 6.71 (d, *J*_H-F_ = 11.77 Hz, 1H), 4.79 (s, 1H) 3.89 (s, 3H), 3.87 (s, 3H); 13C NMR (126 MHz, CDCl_3_): δ_C_ 171.1, 164.2, 153.8, 141.3 (d, *J*_C-F_ = 22.68 Hz), 138.7, 138.4, 136.5, 132.3, 128.6, 128.6, 125.9, 123.5, 123.0, 122.9, 122.5, 111.4, 106.8, 104.0, 56.9, 34.0; ^19^F NMR (470 MHz, CDCl_3_): δ_F_ −147.10; LRMS (ES^+^) **m*/*z** 365.3 [M + H]^+^.

##### 2-Fluoro-4-methoxy-5-((4-(1-methyl-1*H*-indol-3-yl)pyrimidin-2-yl)amino)phenyl (*E*)-3-(5-fluoro-2,4-dioxo-3,4-dihydropyrimidin-1(2*H*)-yl)acrylate, **35**

Synthesized following general procedure C. Purification *via* flash column chromatography (eluent: 0–5% MeOH in DCM) to give the title compound (64 mg, 0.12 mmol, 71%) as a beige solid. *R_f_* = 0.37 (5% MeOH in DCM); mp 278–281 °C; UV λ_max_ = 304, 262 nm; IR: *ν*_max_/cm^–1^ 2928 (m, C–H aromatic), 2853 (w, C–H alkene), 1731 (m, C=O), 1580 (s, C=C aromatic); ^1^H NMR (500 MHz, DMSO-*d*_6_): δ_H_ 12.34 (s, 1H), 8.63 (d, *J*_H-F_ = 6.81 Hz, 1H), 8.40 (d, *J* = 7.94 Hz, 1H), 8.33 (d, *J* = 5.39 Hz, 1H), 8.31 (s, 1H), 8.28–8.18 (m, 2H), 8.04 (s, 1H), 7.52 (d, *J* = 8.18 Hz, 1H), 7.28–7.20 (m, 2H), 7.16 (d, *J* = 7.84 Hz, 1H), 6.56 (d, *J* = 14.50 Hz, 1H), 3.92 (s, 3H), 3.88 (s, 3H); ^13^C NMR (126 MHz, CDCl_3_): δ_C_ 168.9, 162.3, 159.6, 157.2, 149.2, 147.2 (d, *J*_C-F_ = 8.02 Hz), 146.2, 146.1, 138.0, 131.6, 130.5 (d, *J*_C-F_ = 13.19 Hz), 130.3, 126.0, 125.9, 122.6, 121.8, 121.3, 114.0, 113.8, 109.8, 108.4, 99.5, 56.3, 33.3; ^19^F NMR (470 MHz, DMSO-*d*_6_): δ_F_ −134.32, −163.24; LRMS (ES^+^) **m*/*z** 547.3 [M + H]^+^.

##### 3-(2-Chloropyrimidin-4-yl)-1-methyl-1*H*-indole

To a round-bottom flask charged with 2,4-dichloropyrimidine (1.0 g, 6.90 mmol) in 1,2-dimethoxyethane (DME) (13 mL) was added FeCl_3_ (1.1 g, 6.90 mmol) and heated to 60 °C before the addition of 1-methyl-1*H*-indole (1.0 g, 7.70 mmol) and allowed to stir at that temperature for 18 h. After this time, the reaction mixture was cooled to room temperature with a 3:1 mixture of MeOH/H_2_O (100 mL) and then added and stirred for a further 30 min, and the resulting precipitate was then filtered to reveal the desired product (1.20 g, 5.10 mmol, 73%) as a maroon solid. *R_f_* = 0.21 (10% MeOH in DCM); UV λ_max_ (nm) 300, 225; IR: ν_max_/cm^–1^ 3101 (w, C–H), 1565 (s, C=C aromatic); ^1^H NMR (500 MHz, CDCl_3_): δ_H_ 8.46 (d, *J* = 5.35 Hz, 1H), 8.32 (dd, *J* = 5.86, 2.10 Hz, 1H), 7.97 (s, 1H), 7.50 (d, *J* = 5.36 Hz, 1H), 7.41 (dd, *J* = 5.85, 1.98 Hz, 1H), 7.40–7.30 (m, 2H), 3.89 (s, 3H); ^13^C NMR (126 MHz, CDCl_3_): δ_C_ 164.4, 161.5, 158.4, 138.1, 132.7, 125.7, 123.2, 122.1, 121.4, 114.0, 112.5, 110.1, 33.6; LRMS (ES^+^) **m*/*z** 244.1 [M(^35^Cl) + H]^+^ and 246.1 [M(^37^Cl) + H]^+^.

##### *N*-(3-Hydroxyphenyl)acetamide, **36**

To a round-bottom flask charged with 3-aminophenol (1.0 g, 9.2 mmol) in THF (40 mL) was added acetic anhydride (0.9 mL, 9.5 mmol) and left to stir for 2 h. After this time, the reaction mixture was concentrated *in vacuo* to give a gray solid. The desired compound was recrystallized in toluene to give (1.3 g, 8.5 mmol, 93%) as a white solid. *R_f_* = 0.14 (50% petrol in DCM); mp 177–179 °C; UV λ_max_ (nm) 396, 212; IR: ν_max_ /cm^–1^ 3316 (m, O–H phenol), 3204 (w, N–H amide), 3054 (w, C–H aromatic), 2918 (w, C–H alkane), 1603 (s, C=O amide), 1563 (s, C=C aromatic); ^1^H NMR (500 MHz, DMSO-*d*_6_): δ_H_ 9.80 (s, 1H), 9.36 (s, 1H), 7.18 (d, *J* = 2.24 Hz, 1H), 7.03 (dd, *J* = 8.08, 7.91 Hz, 1H), 6.90 (dd, *J* = 8.08, 2.00 Hz, 1H), 6.41 (dd, *J* = 7.91, 2.00 Hz, 1H), 2.01 (s, 3H); ^13^C NMR (126 MHz, DMSO-*d*_6_): δ_C_ 168.6, 158.0, 140.8, 129.7, 110.5, 110.2, 106.6, 24.5; LRMS (ES^+^) **m*/*z** 152.1 [M + H]^+^.

##### *N*-(5-Hydroxy-2-nitrophenyl)acetamide, **37**

To a solution of *N*-(3-hydroxyphenyl)acetamide (500 mg, 3.31 mmol) in TFA (4.5 mL) was added KNO_3_ (500 mg, 4.95 mmol) at 0 °C and allowed to stir for 1 h. After this time, the reaction mixture was poured over ice and stirred for 1 h, producing a red suspension, which was filtered to give the desired compound (456 mg, 2.31 mmol, 70%) as a beige solid. *R_f_* = 0.12 (5% MeOH in DCM); mp 264–266 °C; UV λ_max_ (nm) 373, 339, 319, 284, 252, 206; IR: ν_max_/cm^–1^ 3293 (m, O–H phenol), 3085 (m, N–H amide), 3021 (w, C–H aromatic), 2884 (w, C–H alkane), 1663 (s, C=O amide), 1596 (s, C=C aromatic), 1550 (m, N–O nitro); ^1^H NMR (500 MHz, DMSO-*d*_6_): δ_H_ 11.04 (s, 1H), 10.30 (s, 1H), 7.99 (d, *J* = 9.16 Hz, 1H), 7.53 (d, *J* = 2.61 Hz, 1H), 6.65 (dd, *J* = 9.19, 2.63 Hz, 1H), 2.13 (s, 3H); ^13^C NMR (126 MHz, DMSO-*d*_6_): δ_C_ 169.4, 163.9, 135.9, 132.3, 128.5, 112.1, 109.2, 24.8; LRMS (ES^+^) **m*/*z** 196.1 [M + H]^+^.

##### 3-((4-(1-Methyl-1*H*-indol-3-yl)pyrimidin-2-yl)amino)-4-nitrophenol, **38**

To a round-bottom flask charged with *N*-(5-hydroxy-4-nitrophenyl)acetamide (300 mg, 1.50 mmol) was added 12 M of aq. HCl (12.0 mL, 144 mmol) and stirred at reflux for 2 h, after which time the reaction mixture was concentrated *in vacuo* to give a black oil. To this oil, 3-(2-chloropyrimidin-4-yl)-1-methyl-1*H*-indole (467 mg, 1.93 mmol) and *p*-TSA (360 mg, 1.90 mmol) were added and heated at 110 °C for 18 h. After this time, the reaction mixture was concentrated *in vacuo* to give a black solid. Purification was performed *via* flash column chromatography (eluent: 0–10% MeOH in DCM) to give the title compound (110 mg, 0.30 mmol, 21%) as a green solid. *R_f_* = 0.20 (5% MeOH in DCM); mp 274–276 °C; UV λ_max_ (nm) 380, 300, 229; IR: ν_max_/cm^–1^ 3466 (m, O–H phenol), 3332 (m, N–H aromatic), 3090 (w, C–H aromatic), 2849 (w, C–H alkane), 1576 (s, C=C aromatic), 1498 (m, N–O nitro); ^1^H NMR (700 MHz, DMSO-*d*_6_): δ_H_ 10.86 (s, 1H), 10.17 (s, 1H), 8.64 (dd, *J* = 8.00, 1.87 Hz, 1H), 8.46 (d, *J* = 5.40 Hz, 1H), 8.39 (s, 1H), 8.00 (d, *J* = 9.25 Hz, 1H), 7.92 (d, *J* = 2.28 Hz, 1H), 7.56 (dd, *J* = 8.17, 0.94 Hz, 1H), 7.41 (dd, *J* = 9.33, 2.30 Hz, 1H), 7.39 (d, *J* = 5.40 Hz, 1H), 7.30 (ddd, *J* = 8.20, 6.98, 1.26 Hz, 1H), 7.25 (ddd, *J* = 8.00, 6.99, 1.09 Hz, 1H), 3.90 (s, 3H); ^13^C NMR (126 MHz, DMSO-*d*_6_): δ_C_ 166.9, 162.9, 159.7, 155.9, 149.3, 138.1, 133.9, 128.5, 126.9, 125.8, 122.9, 122.7, 121.6, 112.5, 111.3, 111.0, 109.7, 105.9, 33.6; LRMS (ES^+^) **m*/*z** 362.0 [M + H]^+^.

##### 3-((4-(1-Methyl-1*H*-indol-3-yl)pyrimidin-2-yl)amino)-4-nitrophenyl (*E*)-3-(5-Fluoro-2,4-dioxo-3,4-dihydropyrimidin-1(2*H*)-yl)acrylate, **39**

Synthesized following general procedure C. Purification was performed *via* flash column chromatography (eluent: 0–10% MeOH in DCM) to give the title compound (9 mg, 0.02 mmol, 6%) as a brown solid. *R_f_* = 0.21 (10% MeOH in DCM); mp 289–291 °C; UV λ_max_ (nm) 329, 213; IR: ν_max_/cm^–1^ 3248 (m, N–H aromatic), 3194 (m, N–H carbonate), 3057 (w, C–H alkene), 2923 (m, C–H aromatic), 2850 (w, C–H alkane), 1734 (m, C=O ester), 1694 (s, C=O amide), 1654 (s, C=O urea), 1586 (s, C=C aromatic), 1532 (s, N–O nitro); ^1^H NMR (500 MHz, DMSO-*d*_6_): δ_H_ 12.21 (s, 1H), 10.44 (s, 1H), 8.65 (d, *J* = 6.59 Hz, 1H), 8.57 (m, 2H), 8.49 (d, *J*_H-F_ = 5.36 Hz, 1H), 8.40 (s, 1H), 8.29–8.19 (m, 2H), 7.86–7.80 (m, 2H), 7.57 (d, *J* = 8.01 Hz, 1H), 7.42 (d, *J* = 5.40 Hz, 1H), 7.33–7.15 (m, 2H), 6.54 (d, *J* = 13.91 Hz, 1H), 3.90 (s, 3H); ^13^C NMR (126 MHz, DMSO-*d*_6_): δ_C_ 176.2, 165.0, 164.5, 164.3, 157.4, 156.0, 156.0, 148.4, 147.8, 147.7, 142.9, 139.6, 128.7, 128.7, 127.9, 125.8, 124.1, 123.8 (d, *J*_C-F_ = 36.7 Hz), 110.9, 110.8, 110.8, 110.6, 110.5, 103.2, 55.4; ^19^F NMR (470 MHz, DMSO-*d*_6_): δ_F_ −163.16; LRMS (ES^+^) **m*/*z** 544.3 [M + H]^+^.

##### 1-(3-((4-((3-Chloro-4-fluorophenyl)amino)-7-methoxyquinazolin-6-yl)oxy)azetidin-1-yl)prop-2-yn-1-one, **44**

Synthesized following general procedure A. The resulting precipitate was filtered and dried *in vacuo* to give 1-(3-((4-((3-chloro-4-fluorophenyl)amino)-7-methoxyquinazolin-6-yl)oxy)azetidin-1-yl)prop-2-yn-1-one (55 mg, 0.13 mmol, 60%) as a yellow solid. *R_f_* = 0.18 (20% MeOH in DCM); mp 208–210 °C; UV λ_max_ (nm) 304, 237; IR: ν_max_/cm^–1^ 3267 (w, N–H amide), 2936 (m, C–H aromatic), 1574 (m, C=C alkene), 1527 (m, C=C aromatic), carbonyl stretches not visualized; ^1^H NMR (500 MHz, CDCl_3_): δ_H_ 8.63 (s, 1H), 8.62 (s, 1H), 8.06 (dd, *J*_H-F_ = 6.53 Hz, *J*_H-H_ 2.47 Hz, 1H), 7.89 (dd, *J* = 8.96, 2.59 Hz, 1H), 7.63 (dd, *J*_H-H_ = 8.94 Hz, *J*_H-F_ = 4.14 Hz, 1H), 7.26 (s, 1H), 7.19 (s, 1H), 5.20 (tt, *J* = 10.51, 4.71 Hz, 1H), 4.92 (dd, *J* = 10.42, 6.67 Hz, 2H), 4.44 (dd, *J* = 10.11, 4.71 Hz, 2H), 3.99 (s, 3H), 3.02 (s, 1H); ^13^C NMR (126 MHz, CD_3_OD): δ_C_ 158.9, 157.2, 155.5, 153.8, 147.2, 146.4, 135.9, 124.7 (d, *J*_C-F_ = 8.82 Hz), 122.8, 122.7, 120.1, 116.0 (d, *J*_C-F_ = 22.68 Hz), 108.8, 107.0, 106.5, 106.0, 103.9, 67.5, 60.5, 38.7; ^19^F NMR (470 MHz, CDCl_3_): δ_F_ −121.62; LRMS (ES^+^) **m*/*z** 427.3 [M(^35^Cl) + H]^+^ and 429.3 [M(^37^Cl) + H]^+^.

##### (*R*)-1-(3-((4-((3-Chloro-4-fluorophenyl)amino)-7-methoxyquinazolin-6-yl)oxy)pyrrolidin-1-yl)prop-2-yn-1-one, **45**

Synthesized following general procedure A. The resulting precipitate was filtered and dried *in vacuo* to give (*R*)-1-(3-((4-((3-chloro-4-fluorophenyl)amino)-7-methoxyquinazolin-6-yl)oxy)pyrrolidin-1-yl)prop-2-yn-1-one (97 mg, 0.22 mmol, 76%) as a yellow solid. *R_f_* = 0.12 (20% MeOH in DCM); mp 201–204 °C; [α]_D_^25.6^ = +13.3° (*c* 0.15, EtOH); UV λ_max_ (nm) 330, 247; IR: ν_max_/cm^–1^ 3065 (w, N–H amide), 2958 (w, C–H alkene), 2916 (w, C–H aromatic), 2826 (w, C–H alkane), 2095 (m, C=C alkyne), 1607 (m, C=O amide), 1579 (m, C=C alkene), 1495 (m, C=C aromatic); ^1^H NMR (500 MHz, CDCl_3_): δ_H_ 8.66 (s, 1H), 7.91 (dd, *J*_H-F_ = 6.61 Hz, *J*_H-H_ = 2.70 Hz, 1H), 7.58 (ddd, *J*_H-H_ = 8.80, 2.60 Hz, *J*_H-F_ = 4.28 Hz 1H), 7.46 (s, 1H), 7.34 (s, 1H), 7.29 (s, 1H), 7.15 (d, *J* = 8.80 Hz, 1H). 5.19 (s, 1H), 3.98 (s, 1H), 3.97 (s, 3H), 3.73 (m, 4H), 3.10 (m, 2H); ^13^C NMR (126 MHz, CDCl_3_): δ_C_ 160.1, 156.8, 153.9, 153.8, 146.6, 124.5, 124.1, 122.2, 121.6 (d, *J*_C-F_ = 7.61 Hz), 120.9, 120.8, 117.1 (d, *J*_C-F_ = 17.61 Hz), 116.5, 108.7, 105.4, 85.7, 81.1, 78.2, 56.3, 51.0, 46.5, 43.8; ^19^F NMR (470 MHz, CDCl_3_): δ_F_ −120.73; LRMS (ES^+^) **m*/*z** 441.3 [M(^35^Cl) + H]^+^ and 443.1 [M(^37^Cl) + H]^+^.

##### (*S*)-1-(3-((4-((3-Chloro-4-fluorophenyl)amino)-7-methoxyquinazolin-6-yl)oxy)pyrrolidin-1-yl)prop-2-yn-1-one, **46**

Synthesized following general procedure A. The resulting precipitate was filtered and dried *in vacuo* to give (*S*)-1-(3-((4-((3-chloro-4-fluorophenyl)amino)-7-methoxyquinazolin-6-yl)oxy)pyrrolidin-1-yl)prop-2-yn-1-one (93 mg, 0.21 mmol, 72%) as a yellow solid. *R_f_* = 0.12 (20% MeOH in DCM); mp 201–204 °C; [α]_D_^25.6^ = −5.4° (*c* 0.37, EtOH); UV λ_max_ (nm) 330, 247; IR: ν_max_/cm^–1^ 3065 (w, N–H amide), 2958 (w, C–H alkene), 2916 (w, C–H aromatic), 2826 (w, C–H alkane), 2095 (m, C=C alkyne), 1607 (m, C=O amide), 1579 (m, C=C alkene), 1495 (m, C=C aromatic); ^1^H NMR (500 MHz, CDCl_3_): δ_H_ 8.66 (s, 1H), 7.91 (dd, *J*_H-F_ = 6.61 Hz, *J*_H-H_ 2.70 Hz, 1H), 7.58 (ddd, *J*_H-H_ = 8.80, 2.60 Hz, *J*_H-F_ = 4.28 Hz 1H), 7.46 (s, 1H, *H*^6^), 7.34 (s, 1H, *H*^3^), 7.29 (s, 1H), 7.15 (d, *J* = 8.80 Hz, 1H). 5.19 (s, 1H), 3.98 (s, 1H), 3.97 (s, 3H), 3.73 (m, 4H), 3.10 (m, 2H); ^13^C NMR (126 MHz, CDCl_3_): δ_C_ 160.2, 156.8, 154.0, 153.8, 146.7, 124.5, 124.0, 122.2, 121.6 (d, *J*_C-F_ = 7.58 Hz), 120.9, 120.7, 117.1 (d, *J*_C-F_ = 17.64 Hz), 116.5, 108.7, 105.8, 85.8, 81.2, 78.2, 56.3, 50.9, 46.5, 43.9; ^19^F NMR (470 MHz, CDCl_3_): δ_F_ −120.73 (s, 1F, *F*^18^); LRMS (ES^+^) **m*/*z** 441.3 [M(^35^Cl) + H]^+^ and 443.1 [M(^37^Cl) + H]^+^.

##### 1-(4-((4-((3-Chloro-4-fluorophenyl)amino)-7-methoxyquinazolin-6-yl)oxy)piperidin-1-yl)prop-2-yn-1-one, **47**

Synthesized following general procedure A. The resulting precipitate was filtered and dried *in vacuo* to give 1-(4-((4-((3-chloro-4-fluorophenyl)amino)-7-methoxyquinazolin-6-yl)oxy)piperidin-1-yl)prop-2-yn-1-one (233 mg, 0.51 mmol, 75%) as a yellow solid. *R_f_* = 0.14 (5% MeOH in DCM); mp 206–208 °C; UV λ_max_ (nm) 332, 247; IR: ν_max_/cm^–1^ 2915 (s, C–H aromatic), 2847 (m, C–H alkane), 2105 (w, C=C alkyne), 1613 (s, C=O amide), 1585 (m, C=C aromatic); ^1^H NMR (500 MHz, DMSO-*d*_6_): δ_H_ 9.52 (s, 1H), 8.52 (s, 1H), 8.12 (dd, *J*_H-F_ = 6.85, *J*_H-H_ = 2.64 Hz, 1H), 7.96 (s, 1H), 7.79 (ddd, *J*_H-H_ = 8.95, 2.68 Hz, *J*_H-F_ = 4.28 Hz, 1H), 7.46 (d, *J* = 9.00 Hz, 1H), 7.26 (s, 1H), 4.85–4.78 (m, 1H), 4.56 (s, 1H), 3.96 (s, 3H), 3.76–3.48 (m, 4H), 2.13–1.77 (m, 4H); ^13^C NMR (126 MHz, CDCl_3_): δ_C_ 157.0, 156.5, 153.3, 152.5, 146.9, 136.4, 125.0, 123.70 (d, *J*_C-F_ = 7.56 Hz), 121.2, 119.5, 119.3, 117.0 (d, *J*_C-F_ = 21.42 Hz), 108.7, 106.8, 106.2, 82.7, 76.1, 73.8, 56.6, 38.8, 30.9; ^19^F NMR (470 MHz, DMSO-*d*_6_): δ_F_ −123.05; LRMS (ES^+^) **m*/*z** 455.0 [M(^35^Cl) + H]^+^ and 457.0.

##### (*E*)-1-(3-(3-((4-((3-Chloro-4-fluorophenyl)amino)-7-methoxyquinazolin-6-yl)oxy)azetidin-1-yl)-3-oxoprop-1-en-1-yl)-5-fluoropyrimidine-2,4(1*H*,3*H*)-dione, **48**

Synthesized following general procedure C. Purification was then performed *via* flash column chromatography (eluent: 0–10% MeOH in DCM) to give (*E*)-1-(3-(3-((4-((3-chloro-4-fluorophenyl)amino)-7-methoxyquinazolin-6-yl)oxy)azetidin-1-yl)-3-oxoprop-1-en-1-yl)-5-fluoropyrimidine-2,4(1*H*,3*H*)-dione (30 mg, 0.05 mmol, 58%) as a white solid. *R_f_* = 0.11 (10% MeOH in DCM); UV λ_max_ (nm) 257, 205; IR: ν_max_/cm^–1^ 3123 (m, N–H amide), 2920 (m, C–H aromatic), 2848 (m, C–H alkane), 1719 (s, C=O amide), 1655 (m, C=O amide), 1625 (m, C=O urea), 1576 (m, C=C alkene), 1498 (s, C=C aromatic); ^1^H NMR (500 MHz, DMSO-*d*_6_): δ_H_ 12.21 (s, 1H), 9.60 (s, 1H) 8.58 (d, *J*_H-F_ = 7.25 Hz, 1H), 8.54 (s, 1H), 8.09 (d, *J* = 4.25 Hz, 1H), 7.89 (d, *J* = 14.22 Hz, 1H), 7.60 (s, 1H), 7.48 (m, 2H), 7.29 (s, 1H), 6.43 (d, *J* = 14.12 Hz, 1H), 5.26 (tt, *J* = 10.57, 4.71 Hz 1H), 4.81 (m, 2H), 4.57 (m, 2H), 3.98 (s, 3H); ^13^C NMR (126 MHz, DMSO-*d*_6_): δ_C_ 165.4, 160.6, 158.5, 155.1, 150.5, 146.5, 142.6, 139.3, 135.3, 129.1, 126.7 (d, *J*_C-F_ = 32.20 Hz), 124.5, 123.3 (d, *J*_C-F_ = 8.82 Hz), 122.5, 121.9 (d, *J*_C-F_ = 7.61 Hz), 119.9, 117.2, 117.1 (d, *J*_C-F_ = 22.68 Hz), 106.3, 104.1, 74.4, 56.7, 44.2; ^19^F NMR (470 MHz, DMSO-*d*_6_): δ_F_ −122.76 (*F*^18^), −164.80 (*F*^39^); LRMS (ES^+^) **m*/*z** 557.3 [M(^35^Cl) + H]^+^ and 559.3 [M(^37^Cl) + H]^+^.

##### (*R, E*)-1-(3-(3-((4-((3-Chloro-4-fluorophenyl)amino)-7-methoxyquinazolin-6-yl)oxy)pyrrolidin-1-yl)-3-oxoprop-1-en-1-yl)-5-fluoropyrimidine-2,4(1*H*,3*H*)-dione, **49**

Synthesized following general procedure C. Purification was then performed *via* flash column chromatography (eluent: 0–10% MeOH in DCM) to afford (*R, E*)-1-(3-(3-((4-((3-chloro-4-fluorophenyl)amino)-7-methoxyquinazolin-6-yl)oxy)pyrrolidin-1-yl)-3-oxoprop-1-en-1-yl)-5-fluoropyrimidine-2,4(1*H*,3*H*)-dione (21 mg, 0.04 mmol, 42%) as a white solid. *R_f_* = 0.11 (10% MeOH in DCM); mp 251–254 °C; [α]_D_^25.6^ = +30.0° (*c* 0.20, EtOH); UV λ_max_ (nm) 299, 251, 233; IR: ν_max_/cm^–1^ 3083 (w, N–H amide), 2917 (w, C–H aromatic), 2847 (w, C–H alkane), 1705 (m, C=O amide), 1655 (m, C=O urea), 1577 (s, C=C alkene), 1498 (m, C=C aromatic); ^1^H NMR (500 MHz, DMSO-*d*_6_): δ_H_ 12.11 (s, 1H), 9.57 (s, 1H), 8.52 (d, *J*_H-F_ = 5.80 Hz, 1H), 8.10 (d, *J*_H-F_ = 5.61 Hz, 1H), 7.97 (d, *J* = 11.40 Hz, 1H), 7.87 (s, 1H), 7.78 (s, 1H), 7.47 (d, *J* = 9.20 Hz, 1H), 7.26 (d, *J* = 9.20 Hz, 1H), 7.23 (s, 1H), 6.65 (d, *J* = 11.40 Hz, 1H), 5.32 (m, 1H), 3.95 (s, 3H), 3.94 (m, 4H), 3.79 (m, 2H); ^13^C NMR (126 MHz, DMSO-*d*_6_): δ_C_ 163.4, 160.3, 158.5, 158.5, 156.4, 150.6, 149.6, 146.9, 145.5, 137.4, 127.9, 126.9 (d, *J*_C-F_ = 36.61 Hz), 124.3, 123.2, 119.3 (d, *J*_C-F_ = 21.50 Hz), 117.3, 116.9, 115.1, 113.6, 109.5, 108.3, 76.5, 56.5, 53.4, 52.1, 44.3; ^19^F NMR (470 MHz, DMSO-*d*_6_): δ_F_ −123.01 (*F*^19^), −171.46 (*F*^40^); LRMS (ES^+^) **m*/*z** 571.1 [M(^35^Cl) + H]^+^ and 573.1 [M(^37^Cl) + H]^+^; HRMS calc’d for C_26_H_21_(^35^Cl)F_2_N_6_O_5_ [M + H]^+^ 571.1303, found 571.1252.

##### (*S, E*)-1-(3-(3-((4-((3-Chloro-4-fluorophenyl)amino)-7-methoxyquinazolin-6-yl)oxy)pyrrolidin-1-yl)-3-oxoprop-1-en-1-yl)-5-fluoropyrimidine-2,4(1*H*,3*H*)-dione, **50**

Synthesized following general procedure C. Purification was then performed *via* flash column chromatography (eluent: 0–10% MeOH in DCM) to afford (*S, E*)-1-(3-(3-((4-((3-chloro-4-fluorophenyl)amino)-7-methoxyquinazolin-6-yl)oxy)pyrrolidin-1-yl)-3-oxoprop-1-en-1-yl)-5-fluoropyrimidine-2,4(1*H*,3*H*)-dione (15 mg, 0.03 mmol, 17%) as a white solid. *R_f_* = 0.11 (10% MeOH in DCM); mp 252–255 °C; [α]_D_^25.6^ = −8.0° (*c* 0.25, EtOH); UV λ_max_ (nm) 299, 251, 233; IR: ν_max_/cm^–1^ 3083 (w, N–H amide), 2917 (w, C–H aromatic), 2847 (w, C–H alkane), 1705 (m, C=O amide), 1655 (m, C=O urea), 1577 (s, C=C alkene), 1498 (m, C=C aromatic); ^1^H NMR (500 MHz, DMSO-*d*_6_): δ_H_ 12.11 (s, 1H), 9.57 (s, 1H), 8.52 (d, *J*_H-F_ = 5.80 Hz, 1H), 8.10 (d, *J*_H-F_ = 5.61 Hz, 1H), 7.97 (d, *J* = 11.40 Hz, 1H), 7.87 (s, 1H), 7.78 (s, 1H), 7.47 (d, *J* = 9.20 Hz, 1H), 7.26 (d, *J* = 9.20 Hz, 1H), 7.23 (s, 1H), 6.65 (d, *J* = 11.40 Hz, 1H), 5.32 (m, 1H), 3.95 (s, 3H), 3.94 (m, 4H), 3.79 (m, 2H); ^13^C NMR (126 MHz, DMSO-*d*_6_): δ_C_ 163.8, 160.1, 158.5, 158.4, 156.4, 150.5, 149.6, 146.8, 145.5, 137.2, 127.8, 126.7 (d, *J*_C-F_ = 36.54 Hz), 124.3, 123.2, 119.4 (d, *J*_C-F_ = 22.01 Hz), 117.1, 116.9, 115.4, 113.6, 109.2, 108.3, 76.7, 56.5, 53.4, 52.0, 44.2; ^19^F NMR (470 MHz, DMSO-*d*_6_): δ_F_ −123.01 (*F*^19^), −171.46 (*F*^40^); LRMS (ES^+^) **m*/*z** 571.1 [M(^35^Cl) + H]^+^ and 573.1 [M(^37^Cl) + H]^+^.

##### (*E*)-1-(3-(4-((4-((3-Chloro-4-fluorophenyl)amino)-7-methoxyquinazolin-6-yl)oxy)piperidin-1-yl)-3-oxoprop-1-en-1-yl)-5-fluoropyrimidine-2,4(1*H*,3*H*)-dione, **51**

Synthesized following general procedure C. Purification was performed *via* flash column chromatography (eluent: 0–10% MeOH in DCM) to give (*E*)-1-(3-(4-((4-((3-chloro-4-fluorophenyl)amino)-7-methoxyquinazolin-6-yl)oxy)piperidin-1-yl)-3-oxoprop-1-en-1-yl)-5-fluoropyrimidine-2,4(1*H*,3*H*)-dione (77 mg, 0.13 mmol, 60%) as a white solid. *R_f_* = 0.28 (10% MeOH in DCM); mp 247–249 °C; UV λ_max_ (nm) 294, 249, 226; IR: ν_max_/cm^–1^ 3073 (w, N–H amide), 2922 (s, C–H aromatic), 2852 (m, C–H alkane), 1702 (m, C=O urea), 1658 (m, C=O amide), 1623 (m, C=O amide), 1577 (m, C=C alkene), 1497 (s, C=C aromatic); ^1^H NMR (500 MHz, DMSO-*d*_6_): δ_H_ 12.17 (s, 1H), 9.53 (s, 1H), 8.66 (d, *J*_H-F_ = 7.24 Hz, 1H), 8.52 (s, 1H), 8.12 (dd, *J*_H-F_ = 6.65 Hz, *J*_H-H_ = 2.73 Hz, 1H), 8.00–7.95 (m, 2H), 7.80 (ddd, *J*_H-H_ = 9.02, 2.73 Hz, *J*_H-F_ = 4.30 Hz, 1H), 7.46 (dd, *J* = 9.09, 2.70 Hz, 1H), 7.26 (s, 1H), 6.88 (d, *J* = 13.90 Hz, 1H), 4.86–4.77 (m, 1H), 3.96 (s, 3H), 3.70–3.42 (m, 4H), 2.05–1.73 (m, 4H); ^13^C NMR (126 MHz, DMSO-*d*_6_): δ_C_ 166.3, 164.0, 156.9, 156.8, 156.6, 153.5, 148.2, 147.8, 135.7, 135.2, 127.4, 124.5, 124.2, 123.0, 119.2, 118.1, 117.1, 116.9, 111.7, 109.2, 108.3, 75.7, 56.5, 47.5, 30.6; ^19^F NMR (470 MHz, DMSO-*d*_6_): δ_F_ −123.05 (*F*^18^), −164.94 (*F*^41^); LRMS (ES^+^) **m*/*z** 585.0 [M(^35^Cl) + H]^+^ and 587.0 [M(^37^Cl) + H]^+^.

##### 4-((3-Chloro-4-fluorophenyl)amino)-7-methoxyquinazolin-6-yl (*E*)-3-(5-fluoro-2,4-dioxo-3,4-dihydropyrimidin-1(2*H*)-yl)acrylate **53**

To a stirred solution of (*E*)-3-(5-fluoro-2,4-dioxo-3,4-dihydropyrimidin-1(2*H*)-yl)acrylic acid (36 mg, 0.18 mmol) in DMF (2.0 mL) were added DCC (50 mg, 0.24 mmol) and DMAP (5 mg, 0.04 mmol) and stirred for 5 min at 0 °C after which time 4-((3-chloro-4-fluorophenyl)amino)-7-methoxyquinazolin-6-ol (60 mg, 0.18 mmol) was added and stirred at room temperature overnight. The reaction mixture was then concentrated in vacuo, extracted into EtOAc (2 × 30 mL), washed with brine (50 mL), dried over MgSO_4_, and concentrated in vacuo to give a white solid. Purification was performed via flash column chromatography (eluent: 0–10% MeOH in DCM) to give the desired compound (112 mg, 0.23 mmol, 47%) as a white solid. *R_f_* = 0.37 (10% MeOH in DCM); mp 250–252 °C; UV λ_max_ (nm) 300, 294; IR: ν_max_/cm^–1^ 3414 (m, N–H aromatic), 2922 (w, C–H aromatic), 2849 (w, C–H alkane), 1731 (s, C=O ester), 1631 (s, C=O carbonate), 1569 (s, C=C aromatic); ^1^H NMR (500 MHz, DMSO-*d*_6_): δ_H_ 12.41 (s, 1H), 9.75 (s, 1H), 8.67 (d, *J*_H-F_ = 6.93 Hz, 1H), 8.64 (s, 1H), 8.40 (s, 1H), 8.27 (d, *J* = 14.66 Hz, 1H), 8.23 (dd, *J*_H-F_ = 6.89 Hz, *J*_H-H_ = 2.63 Hz, 1H), 7.83 (ddd, *J*_H-H_ = 9.05, 2.65 Hz, *J*_H-F_ = 4.28 Hz, 1H), 7.45 (dd, *J* = 9.08, 2.65 Hz, 1H), 7.40 (s, 1H), 6.60 (d, *J* = 14.66 Hz, 1H), 3.96 (s, 3H); ^13^C NMR (126 MHz, DMSO) δ 164.6, 157.3, 157.2 (d, *J*_C-F_ = 26.3 Hz), 156.0, 155.1, 151.7 (d, *J* = 254.9 Hz), 148.4, 141.9 (d, *J*_C-F_ = 237.4 Hz), 139.5, 137.0, 124.1, 123.8, 123.5, 122.2 (d, *J* = 6.8 Hz), 119.4 (d, *J*_C-F_ = 18.2 Hz), 116.9, 116.8 (d, *J*_C-F_ = 6.9 Hz), 109.1, 108.8, 103.1, 56.8; ^19^F NMR (470 MHz, DMSO-*d*_6_): δF −122.90, −163.01; LRMS (ES^+^) **m*/*z** 502.3 [M(^35^Cl) + H]^+^ and 504.3 [M(^37^Cl) + H]^+^.

##### Methyl (*E*)-4-(Piperidin-1-yl)but-2-enoate, **54**

To a solution of methyl 4-bromocrotonate (0.13 mL, 1.12 mmol) in DCM (3 mL) was added piperidine (0.16 mL, 1.12 mmol) followed by K_2_CO_3_ (309 mg, 2.24 mmol) and allowed to stir at room temperature for 4.5 h. After this time, the reaction mixture was filtered and the resulting filtrate was concentrated in vacuo to give the desired ester (167 mg, 0.91 mmol, 81%) as an off-white solid. *R_f_* = 0.20 (100% DCM); ^1^H NMR (500 MHz, CDCl_3_): δ_H_ 6.86 (dt, *J* = 15.72, 6.27 Hz, 1H), 5.85 (dt, *J* = 15.68, 1.63 Hz, 1H), 3.61 (s, 3H), 3.12 (dd, *J* = 6.26, 1.66 Hz, 2H), 2.47–2.34 (m, 4H), 1.65–1.67 (m, 4H), 1.54–1.38 (m, 2H); ^13^C NMR (126 MHz, CDCl3): δ_C_ 166.7, 146.1, 122.6, 60.1, 54.7, 51.5, 26.0, 24.1; LRMS (ES^+^) **m*/*z** 184.1 [M + H]^+^.

##### (*E*)-4-(Piperidin-1-yl)but-2-enoic Acid, **55**

To a solution of methyl (*E*)-4-(piperidin-1-yl)but-2-enoate (162 mg, 0.88 mmol) in THF (1.6 mL) was added 2 M NaOH (1.6 mL, 3.2 mmol) and left to stir at room temperature overnight. After this time, the reaction mixture was concentrated in vacuo forming a white solid, which was suspended in 10% MeOH in DCM and filtered. The resulting filtrate was concentrated in vacuo to give the desired acid (45 mg, 0.26 mmol, 30%) as a white solid. *R_f_* = 0.10 (10% MeOH in DCM); ^1^H NMR (500 MHz, DMSO-*d*_6_): δ_H_ 8.73 (s, 1H), 6.88 (dt, *J* = 15.61, 7.17 Hz, 1H), 6.17 (d, *J* = 15.68, 1.41 Hz, 1H), 3.87 (d, *J* = 7.17, 1.41 Hz, 2H), 2.92–2.77 (m, *J* = 12.04 Hz, 2H), 1.88–1.55 (m, 6H); ^13^C NMR (126 MHz, DMSO-*d*_6_): δ_C_ 166.4, 136.1, 129.8, 56.1, 52.4, 22.8, 21.6; LRMS (ES^+^) **m*/*z** 168.1 [M + H]^+^.

##### 4-((3-Chloro-4-fluorophenyl)amino)-7-methoxyquinazolin-6-yl (*E*)-4-(piperidin-1-yl)but-2-enoate, **56**

To a round-bottom flask charged with (*E*)-4-(piperidin-1-yl)but-2-enoic acid (800 mg, 4.8 mmol) was added DMF (13 mL) followed by DCC (978 mg, 4.8 mmol) at 0 °C and stirred for 5 min before the addition of DMAP (50 mg, 0.4 mmol) and stirred for a further 5 min. After this time, 4-((3-chloro-4-fluorophenyl)amino)-7-methoxyquinazolin-6- ol (150 mg, 0.47 mmol) was added and allowed to stir at room temperature for 18 h. The reaction mixture was then concentrated in vacuo, extracted into DCM (2 × 20 mL), washed with sat. aq. NaHCO_3_ (30 mL), brine (30 mL), dried over MgSO_4_, and concentrated in vacuo to give a beige solid. Purification was performed via flash column chromatography (eluent: 0–10% MeOH in DCM) to give the title compound (113 mg, 0.24 mmol, 51%) as a beige solid. *R_f_* = 0.20 (10% MeOH in DCM); mp 231–233 °C; UV λ_max_ (nm) 298, 265; IR: ν_max_/cm^–1^ 3247 (w, N–H aromatic), 2940 (w, C–H aromatic), 2860 (w, C–H alkane), 1664 (m, C=O ester), 1598 (m, C=C alkene), 1497 (m, C=C aromatic); ^1^H NMR (500 MHz, DMSO-*d*_6_): δ_H_ 9.70 (s, 1H), 8.63 (s, 1H), 8.36 (s, 1H), 8.22 (dd, *J*_H-F_ = 6.91 Hz, *J*_H-H_ = 2.59 Hz, 1H), 7.82 (ddd, *J*_H-H_ = 9.21, 2.64 Hz, *J*_H-F_ = 4.35 Hz, 1H), 7.45 (dd, *J* = 9.06, 1.50 Hz, 1H), 7.39 (s, 1H), 7.14 (dt, *J* = 15.67, 5.86 Hz, 1H), 6.34 (d, *J* = 15.80 Hz, 1H), 3.95 (s, 3H), 3.21 (m, 2H), 2.39 (m, 4H), 1.54 (m, H30), 1.41 (m, 2H); ^13^C NMR (126 MHz, DMSO-*d*_6_): δ_C_ 163.3, 162.8, 156.4, 152.3, 147.3, 132.5, 129.3, 124.2, 124.0, 123.3, 122.2 (d, *J*_C-F_ = 6.82 Hz), 119.0 (d, *J*_C-F_ = 18.31 Hz), 117.1, 116.9 (d, *J*_C-F_ = 21.56 Hz), 114.6, 110.6, 105.6, 57.0, 46.6, 42.9, 25.8, 24.5; ^19^F NMR (470 MHz, DMSO-*d*_6_): δ_F_ −122.90; LRMS (ES^+^) **m*/*z** 471.1 [M(^35^Cl) + H]^+^ and 473.1 [M(^37^Cl) + H]^+^.

##### (*E*)-*N*-(4-((3-Chloro-4-fluorophenyl)amino)-7-methoxyquinazolin-6-yl)-3-(5-fluoro-2,4-dioxo-3,4-dihydropyrimidin-1(2*H*)-yl)acrylamide, **57**

To a stirred solution of (*E*)-3-(5-fluoro-2,4-dioxo-3,4-dihydropyrimidin-1(2*H*)-yl)acrylic acid **58** (300 mg, 1.51 mmol) in THF (1.3 mL) were slowly added oxalyl chloride (0.13 mL, 1.51 mmol) and DMF (2 drops). After 30 min at room temperature, the reaction mixture was added dropwise to a stirred solution of *N*^4^-(3-chloro-4-fluorophenyl)-7-methoxyquinazoline-4,6-diamine **58** (240 mg, 0.753) and pyridine (83.0 μL, 0.941 mmol) in THF (7.0 mL). The reaction mixture was stirred at room temperature overnight and then concentrated under reduced pressure. The crude material was purified by flash column chromatography eluting with methanol (0–50%) in dichloromethane. The material was further purified by trituration with ethyl acetate to yield the title compound as a pale yellow solid (30.1 mg, 60.2 μmol, 8%). *R_f_* = 0.17 (10% MeOH in DCM); IR: ν_max_/cm^–1^ 3365 (w, N–H aromatic), 1694 (s, C=O amide), 1632 (s, C=C alkene), 1572 (m, C=C aromatic) 1525 (s, C=C aromatic), 1497 (s, C=C aromatic); ^1^H NMR (500 MHz, DMSO) δ 12.31 (d, *J* = 5.1 Hz, 1H), 11.30 (s, 1H), 9.80 (s, 1H), 9.25 (s, 1H), 8.89 (s, 1H), 8.26 (d, *J* = 6.7 Hz, 1H), 7.99 (dd, *J* = 14.2, 1.6 Hz, 1H), 7.95 (dd, *J* = 6.8, 2.6 Hz, 1H), 7.67 (ddd, *J* = 8.9, 4.3, 2.6 Hz, 1H), 7.49 (t, *J* = 9.0 Hz, 1H), 6.89 (d, *J* = 14.1 Hz, 1H), 4.10 (s, 3H); ^13^C NMR (126 MHz, DMSO) δ 164.08, 159.17, 157.31, 157.02 (d, *J* = 20.1 Hz), 155.60 (d, *J* = 246.6 Hz), 150.37, 148.45, 141.52 (d, *J* = 236.3 Hz), 135.35, 134.66, 129.70, 127.07, 125.63 (d, *J* = 7.4 Hz), 124.52 (d, *J* = 36.1 Hz), 119.74 (d, *J* = 18.7 Hz), 117.22 (d, *J* = 21.9 Hz), 115.77, 109.64, 107.69, 100.37, 57.34; ^19^F NMR (470 MHz, DMSO-*d*_6_): δ_F_ 119.1, 164.3.

## Supporting Information Available

Molecular formula strings table (CSV)
Supplementary figures, NMR spectra, and spectroscopic evidence for regiochemistry of 5FU addition and HPLC traces (PDF)

## Abbreviations Used

5FU: 5-fluorouracil
ATP: adenosine triphosphate
CoLDR: covalent ligand-directed release
EGFR: epidermal growth factor receptor
NSCLC: non-small cell lung cancer
