# Supplementary material for: Targeting Cytotoxic Agents through EGFR-Mediated Covalent Binding and Release
Source: J Med Chem. 2023 Aug 30;66(17):12324–41. doi: 10.1021/acs.jmedchem.3c00845 (PMC10510387; doi:10.1021/acs.jmedchem.3c00845)
Supplement: Supplementary file 2 — jm3c00845_si_002.pdf [file jm3c00845_si_002.pdf]

Supporting Information

Targeting cytotoxic agents through EGFR-mediated covalent binding and release

Pasquale A. Morese,[a] Nahoum Anthony,[b] Michael Bodnarchuk,[c] Claire Jennings,[b] Mathew P. Martin,[b] Richard A. Noble,[b] Nicole Phillips,[b] Huw D. Thomas,[b] Lan Z. Wang, Andrew Lister,[c] Martin E. M. Noble,[b] Richard A. Ward,[c] Stephen R. Wedge,[b] Hannah L. Stewart [a]* and Michael J. Waring.[a]*

[a] Cancer Research Horizons Therapeutic Innovation, Newcastle University Centre for Cancer, Chemistry, School of Natural and Environmental Sciences, Newcastle University, Bedson Building, Newcastle upon Tyne, NE1 7RU, U.K.

[b] Cancer Research Horizons Therapeutic Innovation, Newcastle University Centre for Cancer, Translational and Clinical Research Institute, Faculty of Medical Sciences, Paul O'Gorman Building, Newcastle University, Newcastle upon Tyne, NE2 4HH, U.K.

[c] Oncology iMed, R&D, AstraZeneca, Cambridge, CB4 0WG, UK.

Corresponding author emails: hannah.stewart@ncl.ac.uk; mike.waring@ncl.ac.uk

Contents

Supplementary Figures S2

NMR Spectra S7

Assignment of 5FU addition – Compound **16** S10

Analytical HPLC Traces S13

Supplementary Figures

Table S1. WT and DM EGFR potencies determined by TR FRET (n=1, 2 technical replicates).

Compound	DM EGFR pIC_{50}	WT EGFR pIC_{50}
**30**	6.8	5.7
**35**	5.5	<4.0
**39**	3.7	<4.0
**48**	6.7	6.6
**49**	6.4	5.6
**50**	6.6	5.9
**51**	n.d.	6.6

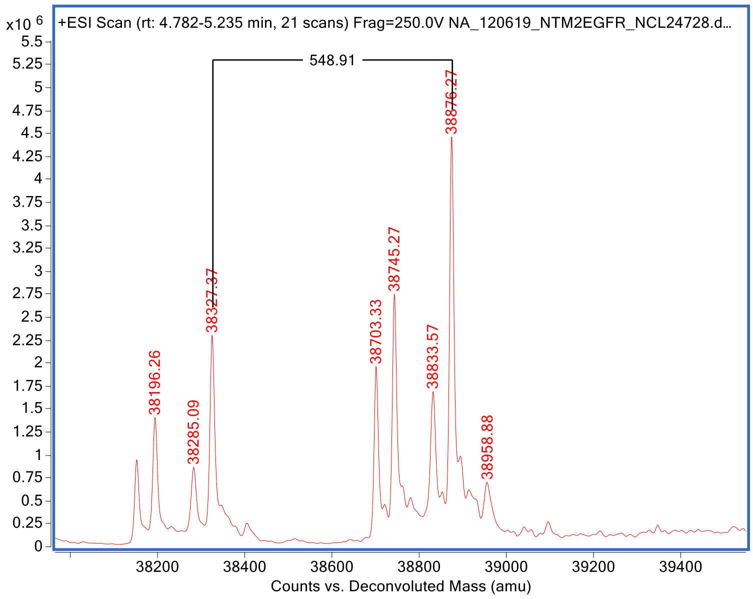

Figure S1. Protein mass spectrometry spectrum showing the mass addition of 548 to the protein, corresponding to the addition of the intact hybrid **30** without 5FU release.

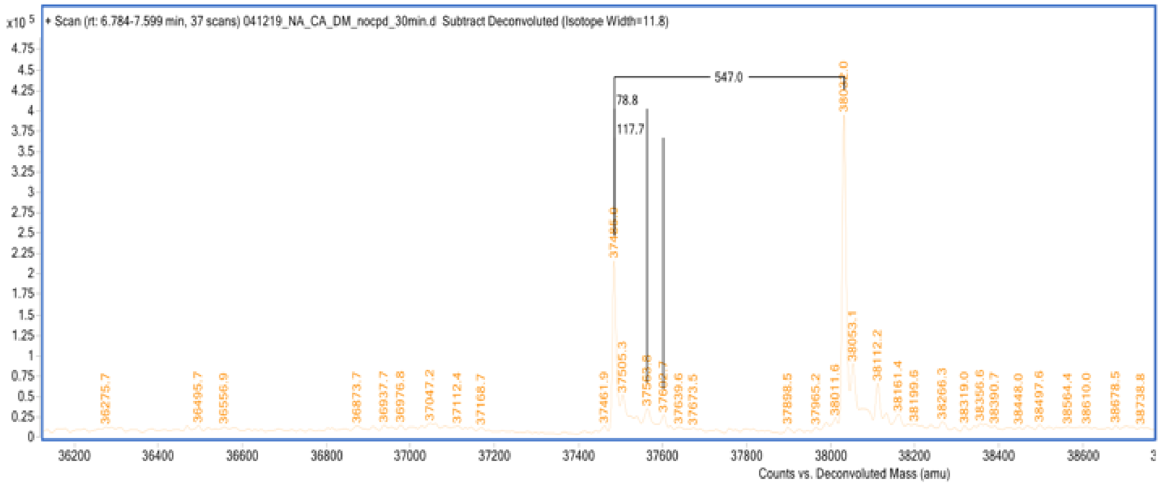

Figure S2. Protein mass spectrometry spectrum showing the mass addition of 547 to the protein, corresponding to the addition of the intact hybrid **35** without 5FU release.

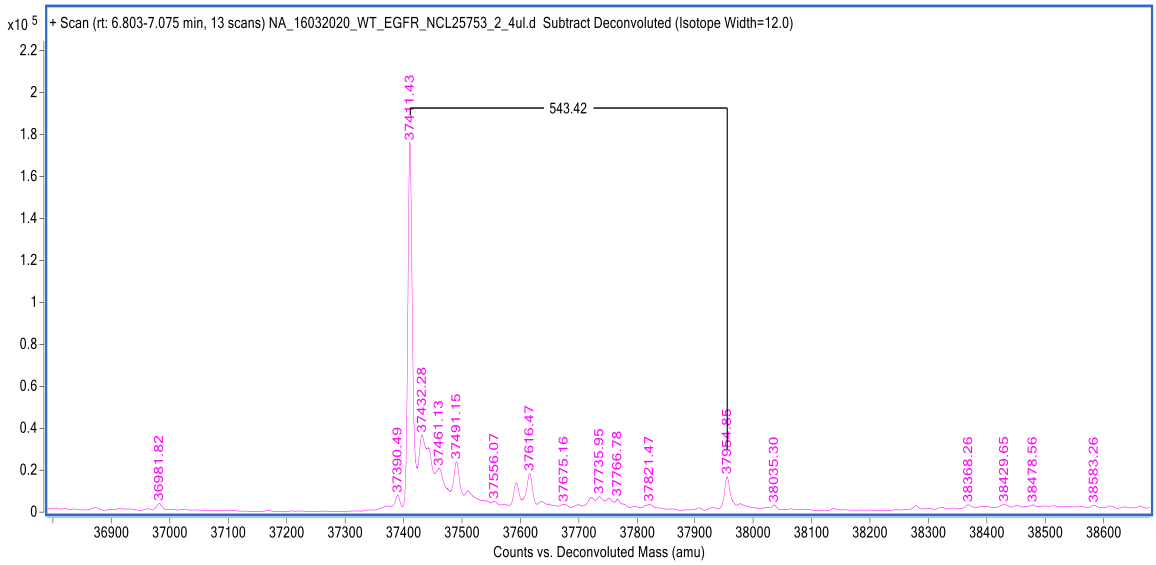

Figure S3. Protein mass spectrometry spectrum showing the addition of 543 Daltons to the protein, corresponding to the addition of the hybrid **39** without release of 5FU.

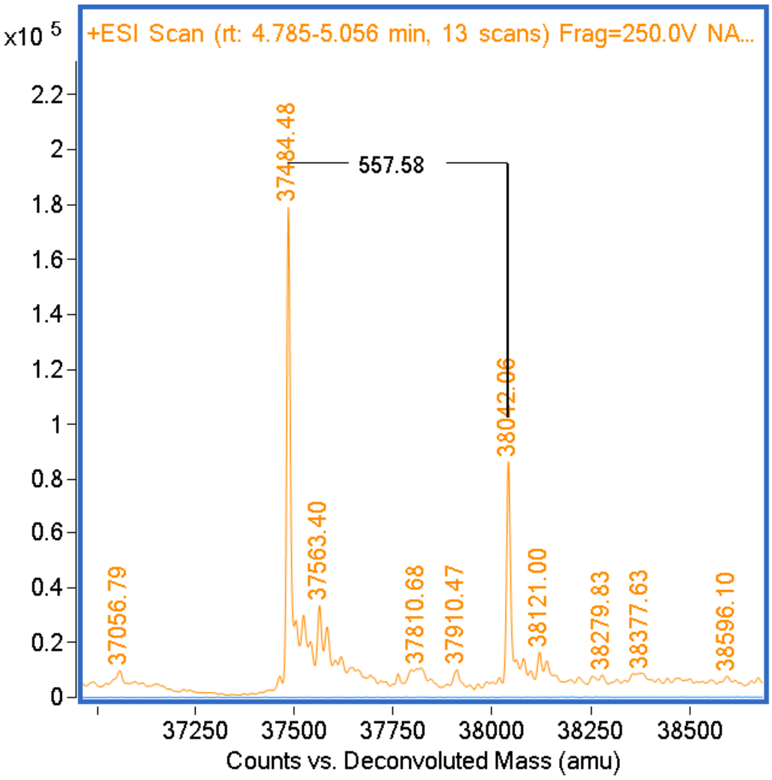

Figure S4. Protein mass spectrometry spectrum showing the addition of 557 Daltons to the protein, corresponding to the addition of the conjugate **48** without release of 5FU.

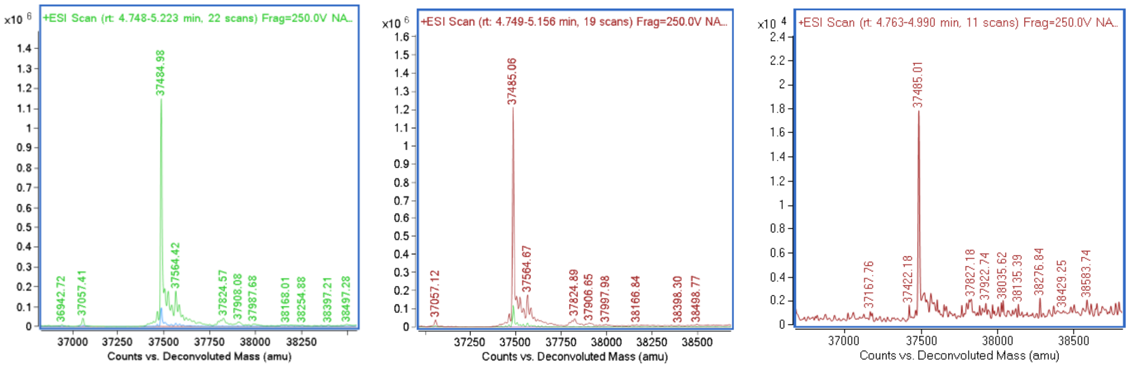

Figure S5. Protein mass spectrometry spectra of incubation of **49-51** with EGFR protein without release of 5FU.

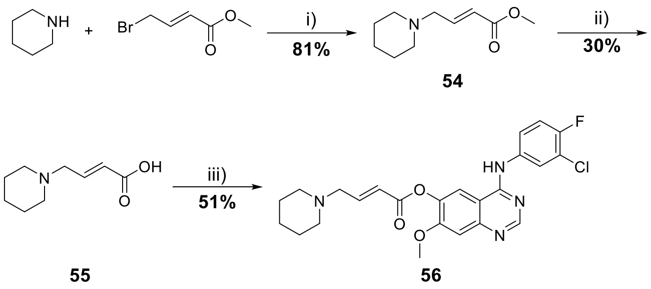

Scheme S1. Synthesis of the acrylate analogue of dacomitinib, **56**. Reagents and conditions: i) K_2CO_3, DCM, rt, 4.5 h; ii) 2 M aq. NaOH, THF, rt, 18 h; iii) %FU, DCC, DMAP; DMF, 0 °C-rt, 18 h.

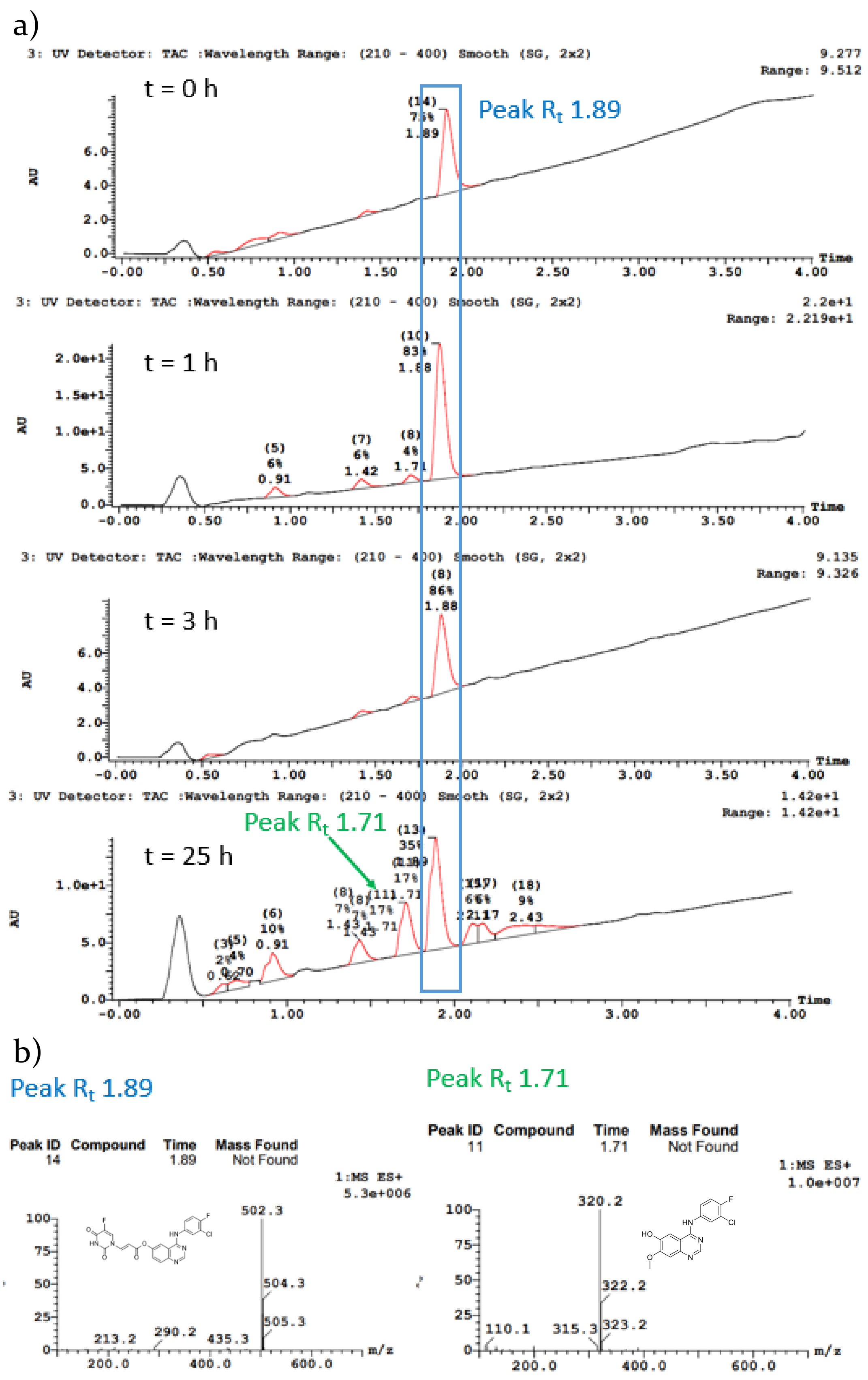

Figure S6. a) LCMS traces of compound **53** incubated in media and analyzed at t = 0, 1, 3 & 25 hours by LCMS analysis; b) mass spectra of main peaks and corresponding structures.

<u>NMR Spectra</u>

NMR data for compound 53

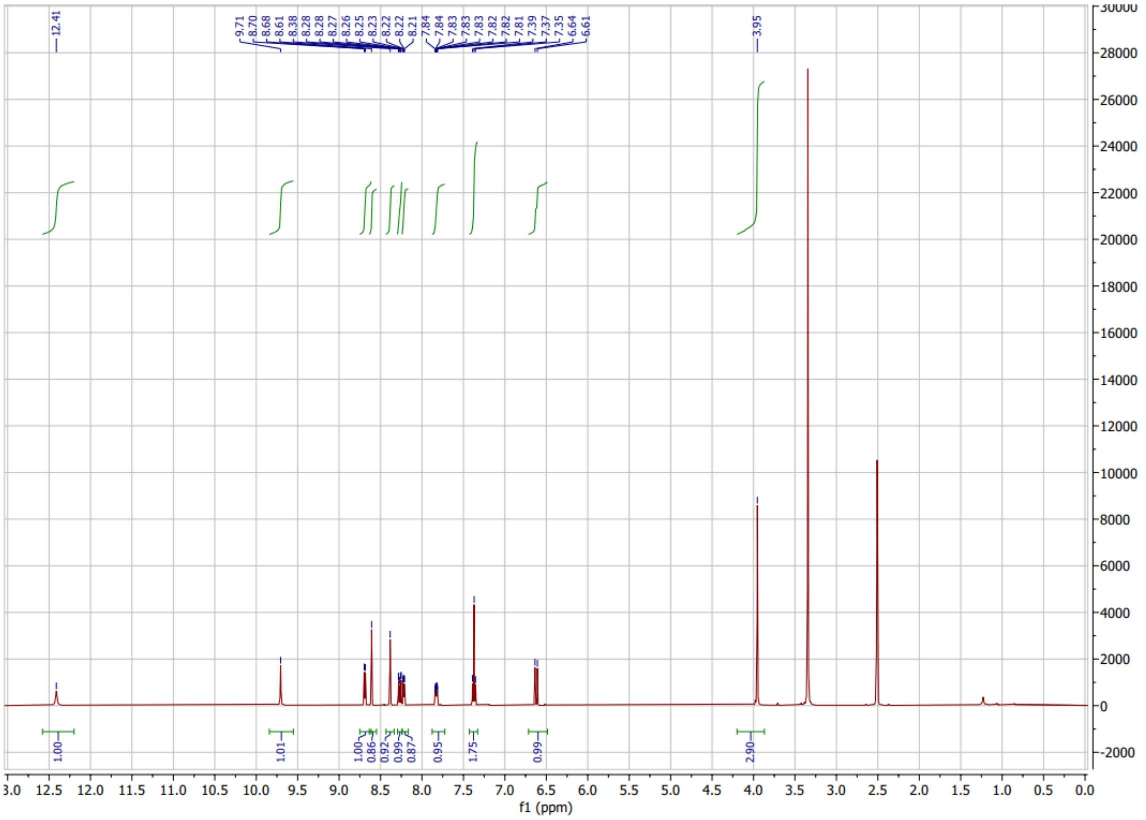

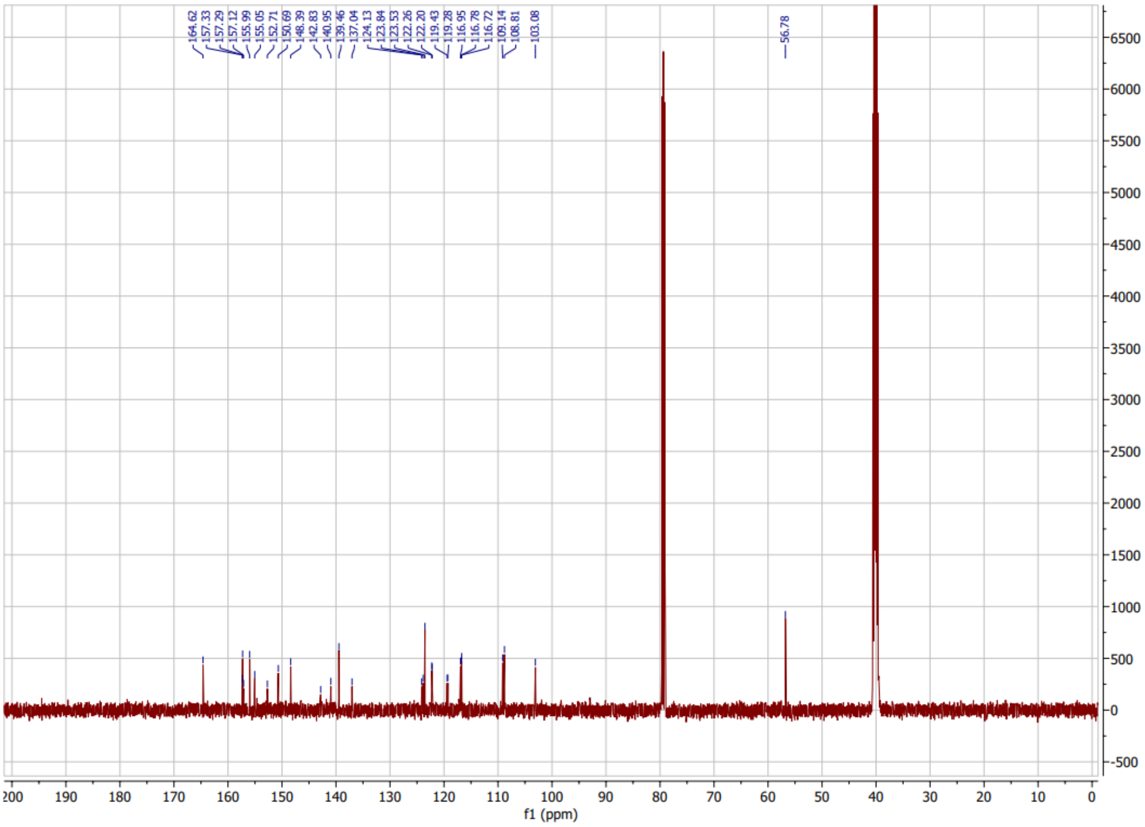

NMR data for compound 56

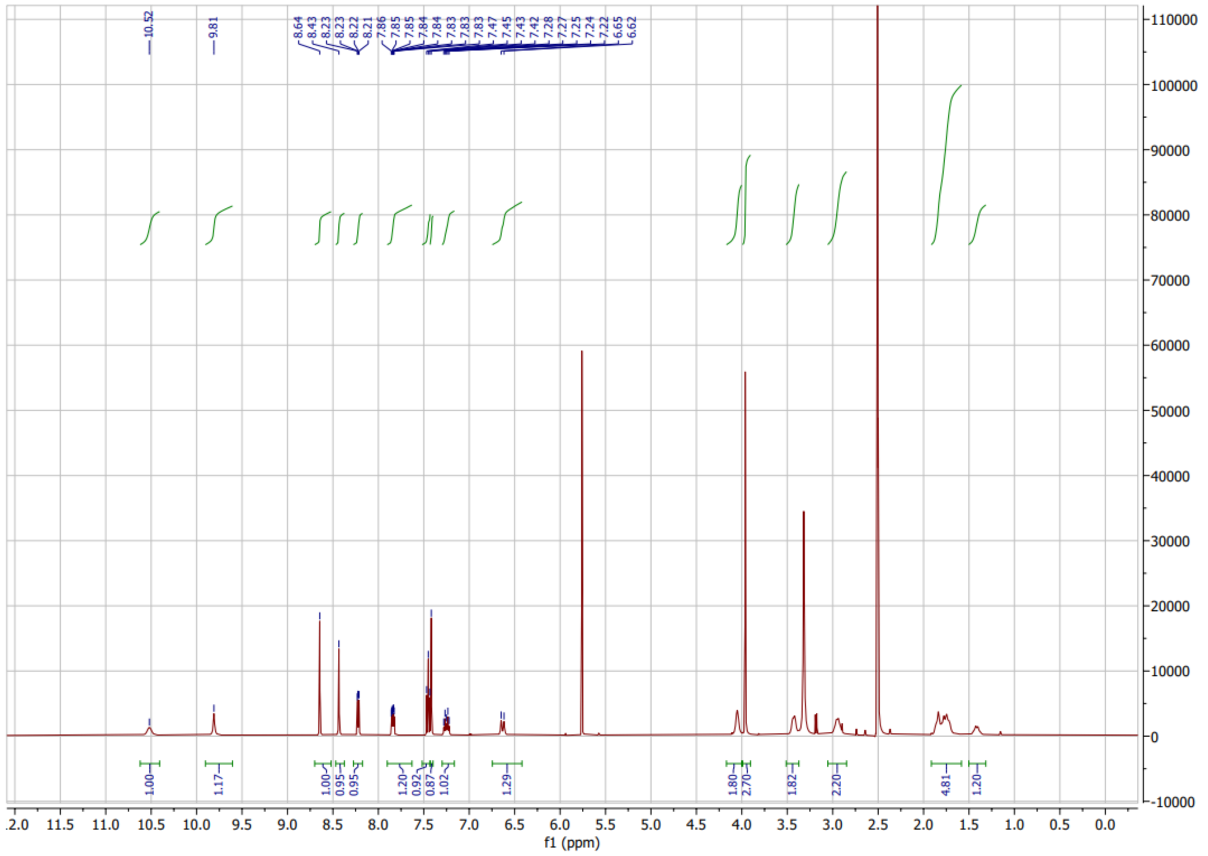

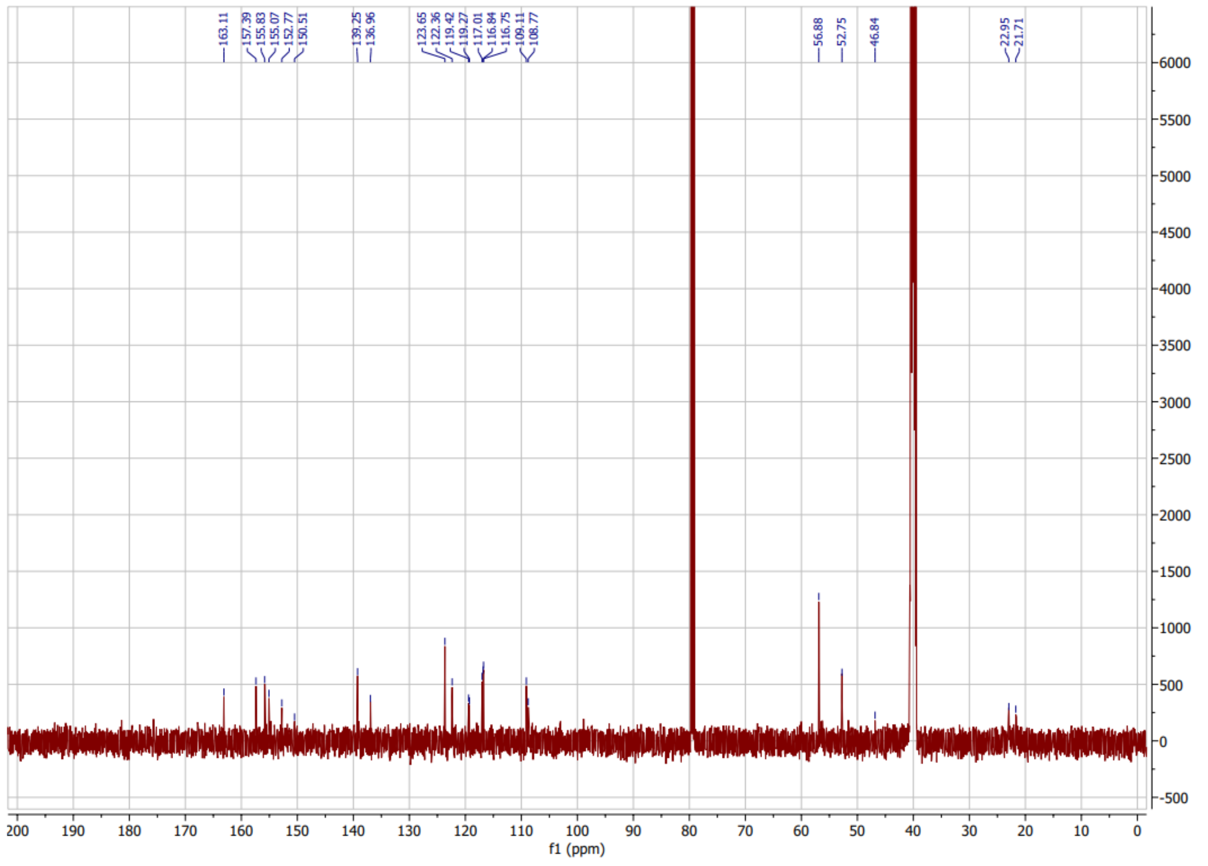

NMR data for compound 57

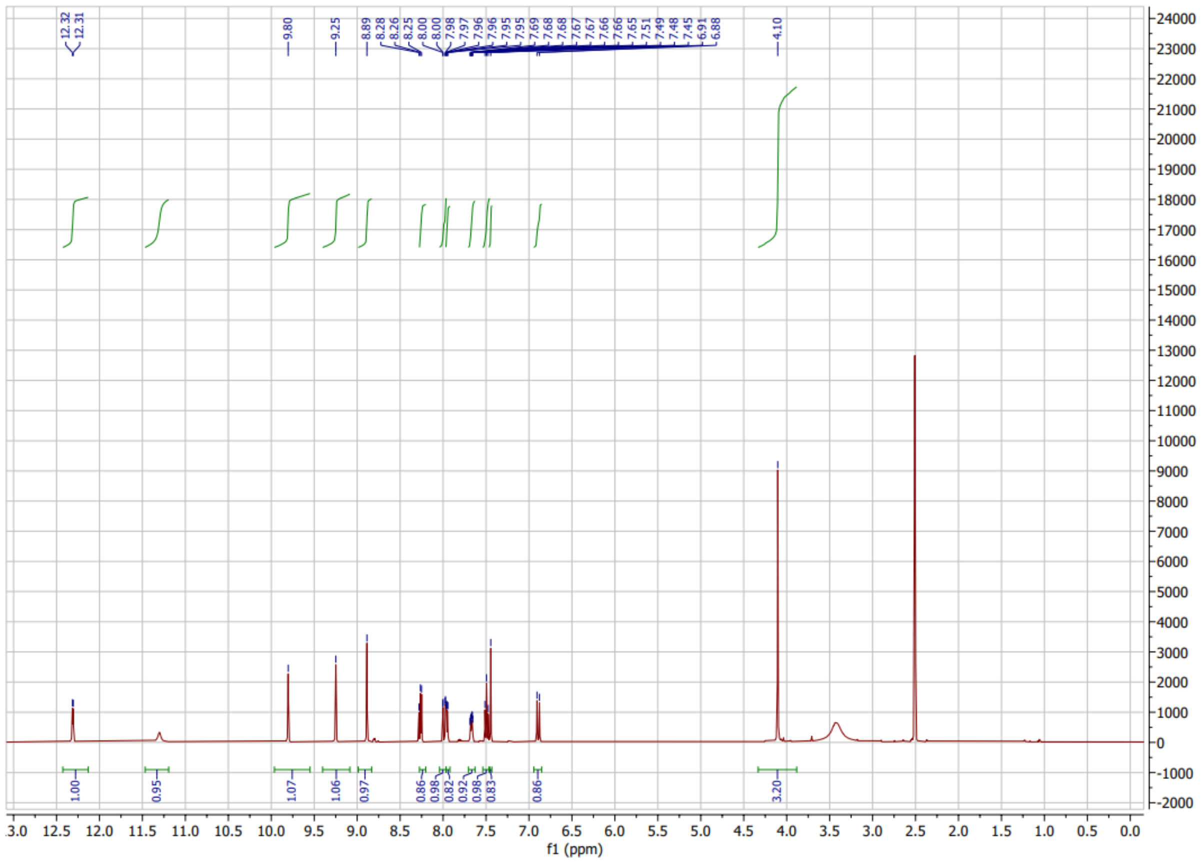

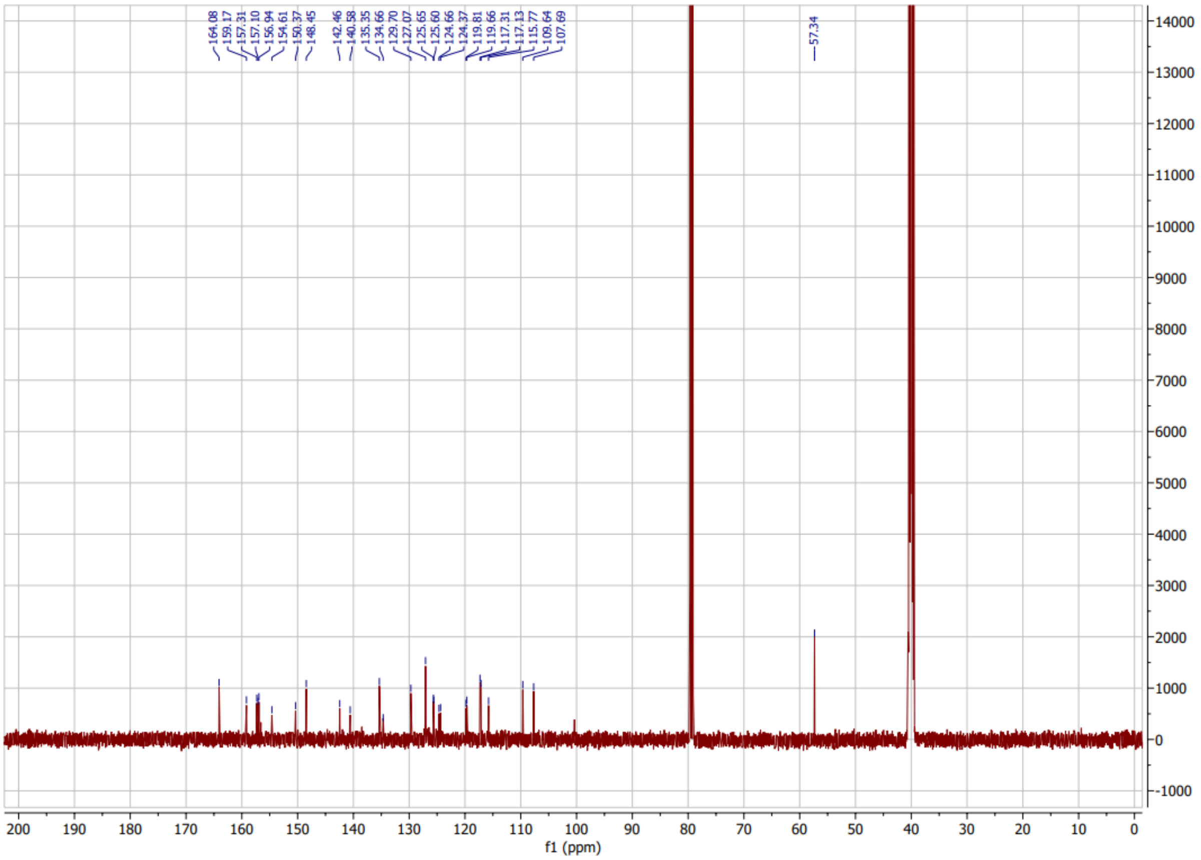

Assignment of 5FU addition – Compound **16**

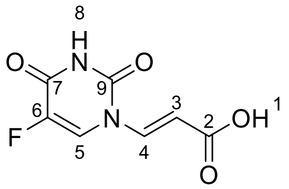

^{1}H NMR (500 MHz, DMSO) δ 12.55 (s, 1H, H1/2), 12.29 (s, 1H, H1/2), 8.56 (d, *J* = 7.0 Hz, 1H, H5), 7.96 (dd, *J* = 14.6, 1.8 Hz, 1H, H4), 6.19 (d, *J* = 14.5 Hz, 1H, H3).

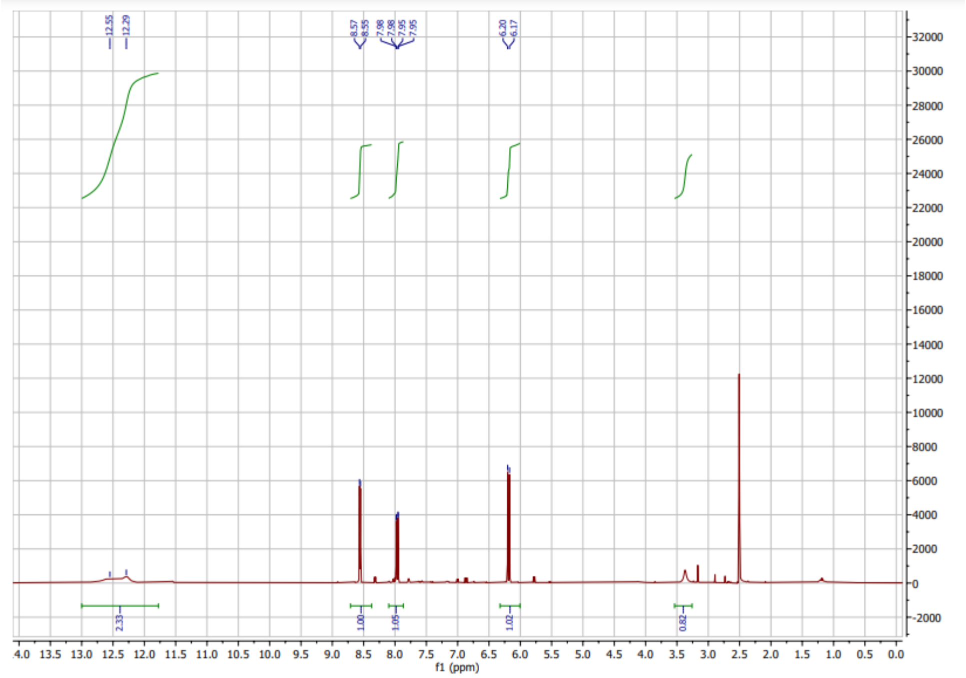

^{13}C NMR (126 MHz, DMSO) δ 167.7 (C2), 157.4 (d, J = 26.5 Hz, C7), 148.5 (C9), 142.6 (d, *J* = 235.7 Hz, C6), 137.0 (C4), 124.3 (d, *J* = 36.8 Hz, C5), 106.03 (C3).

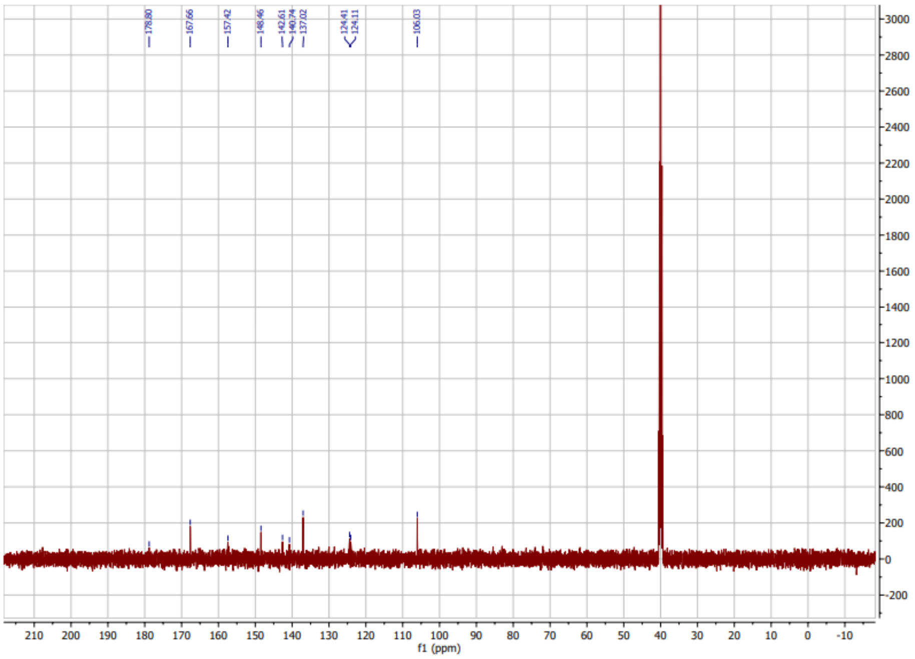

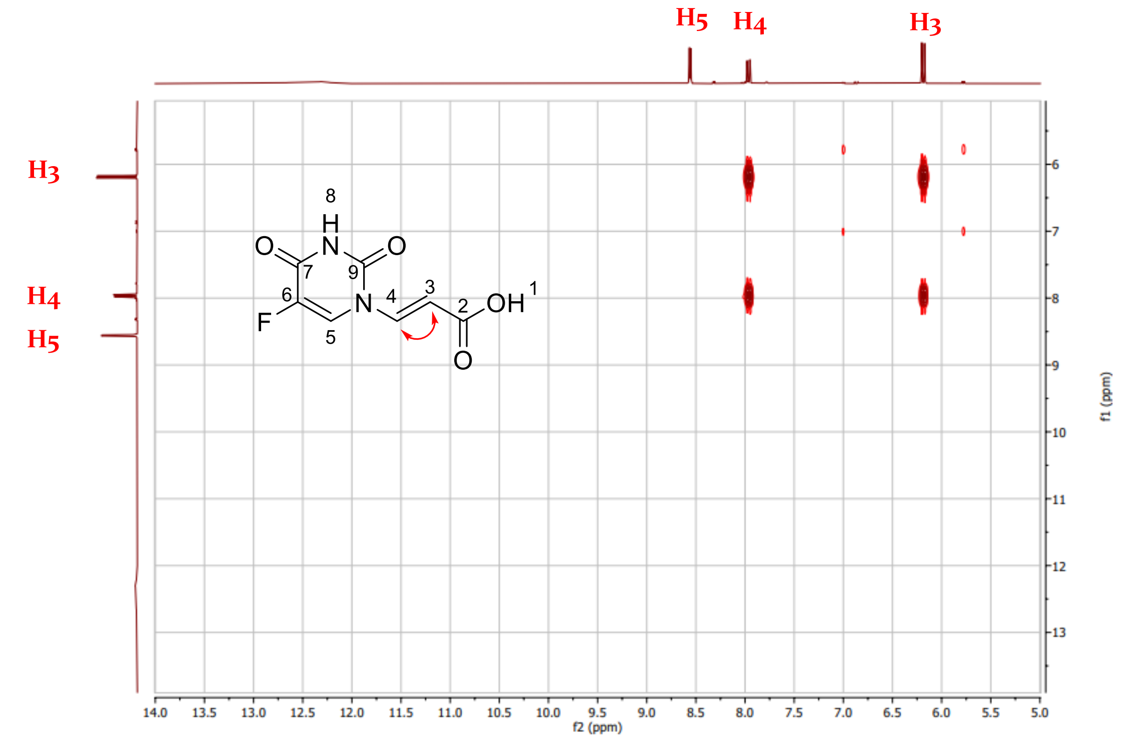
H5
H4
H3
f2 (ppm)
f1 (ppm)
OH
F
O
N

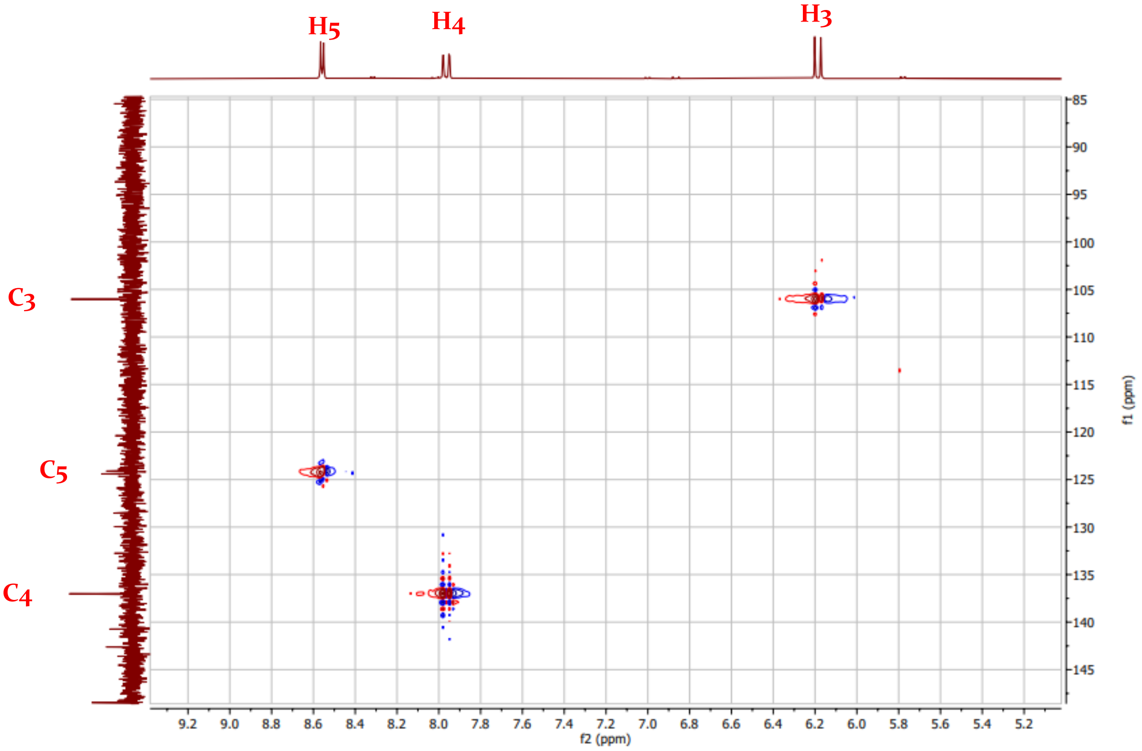
H5
H4
H3
C3
C5
C4
f2 (ppm)
f1 (ppm)

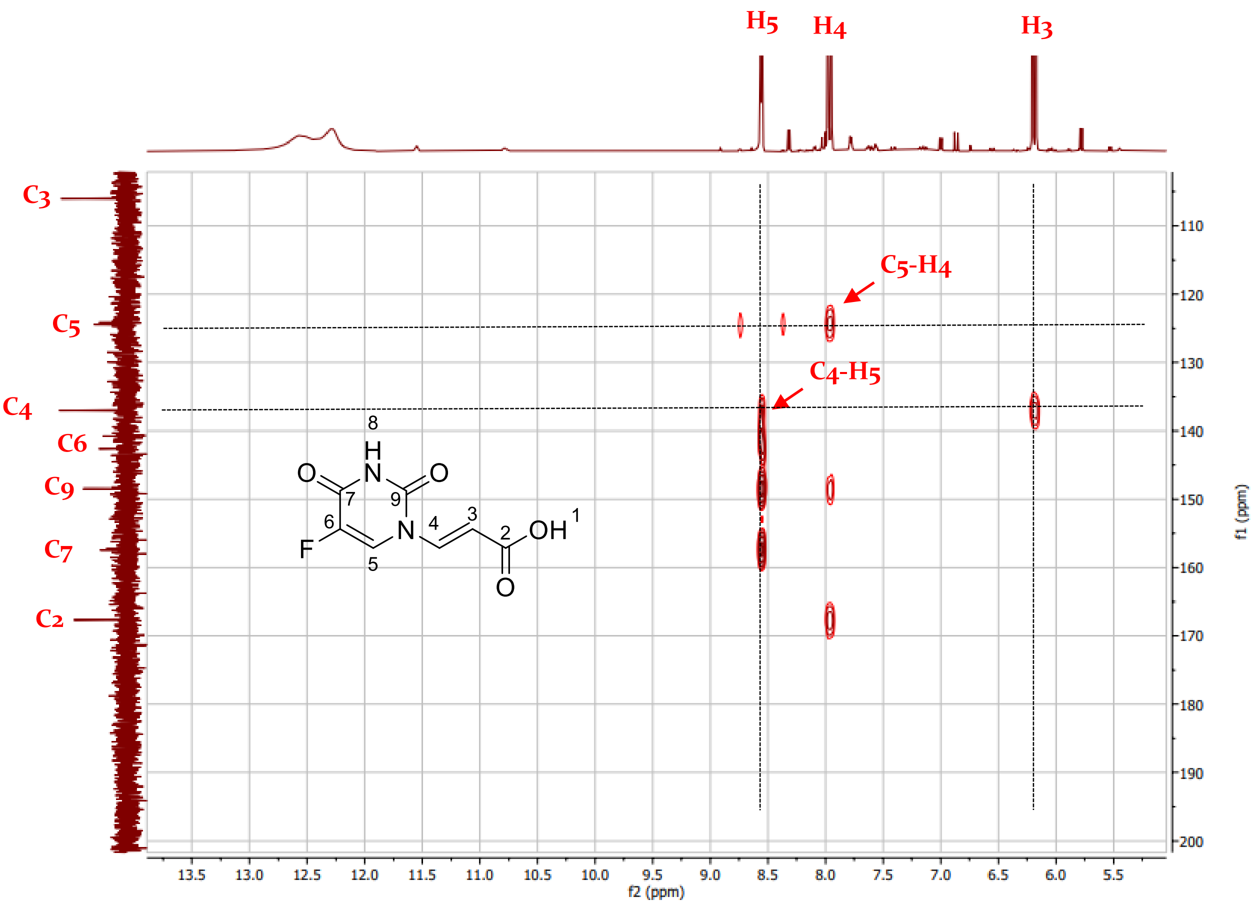
H5
H4
H3
C3
C5
C4
C6
C9
C7
C2
C5-H4
C4-H5
f2 (ppm)
f1 (ppm)

Analytical HPLC Traces

HPLC trace for compound 2

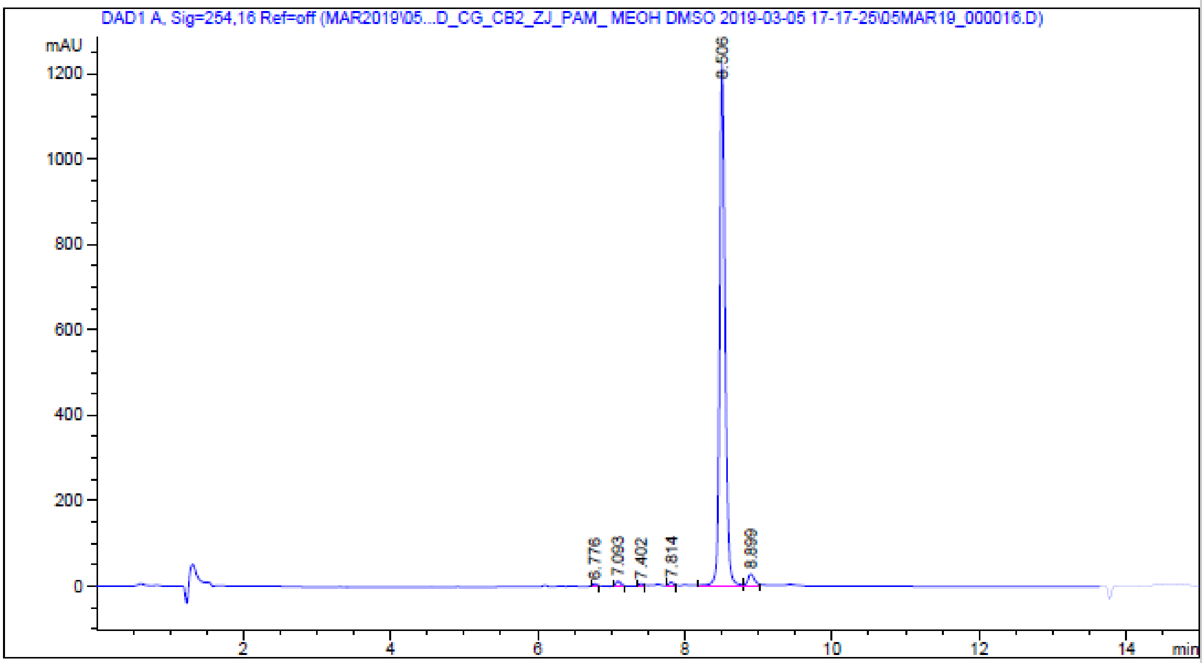

Peak area 95.0%

HPLC trace for compound 53

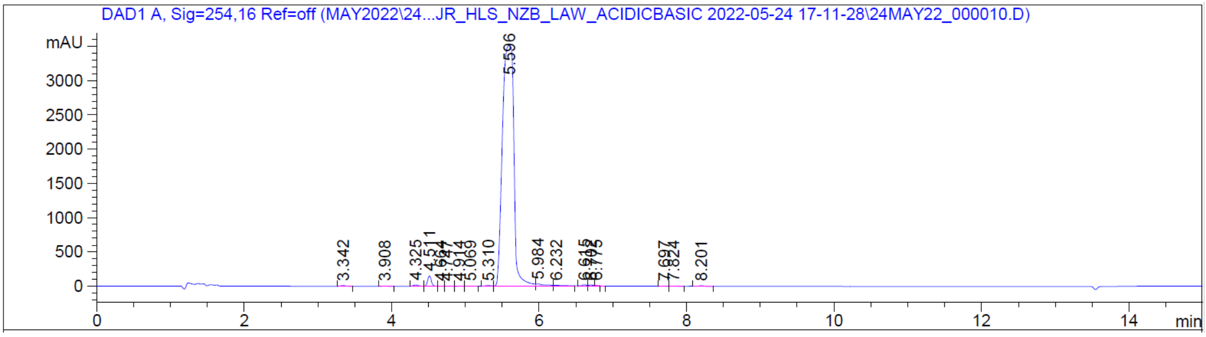

Peak area 96.2%

HPLC trace for compound 56

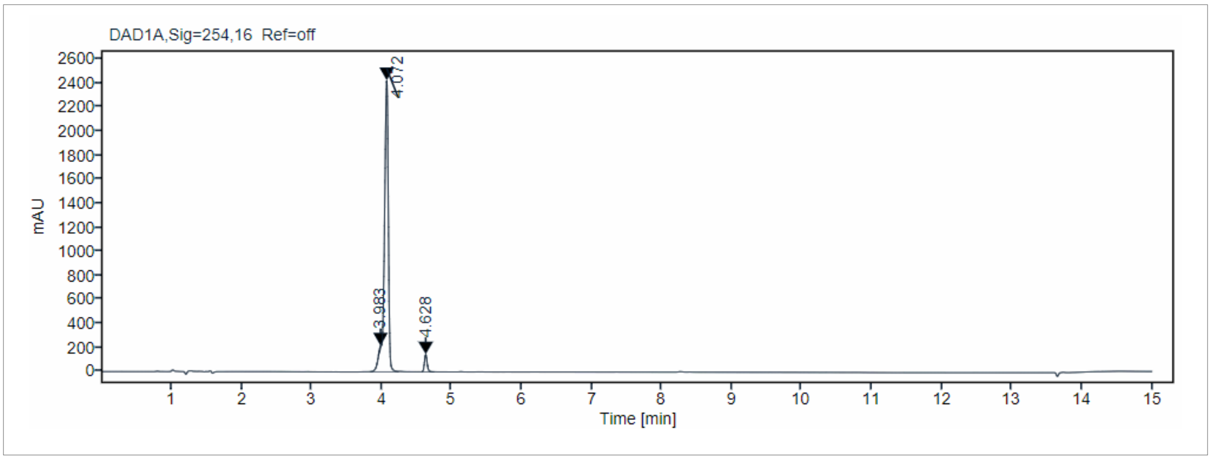

Peak area 95.6%

HPLC trace for compound 57

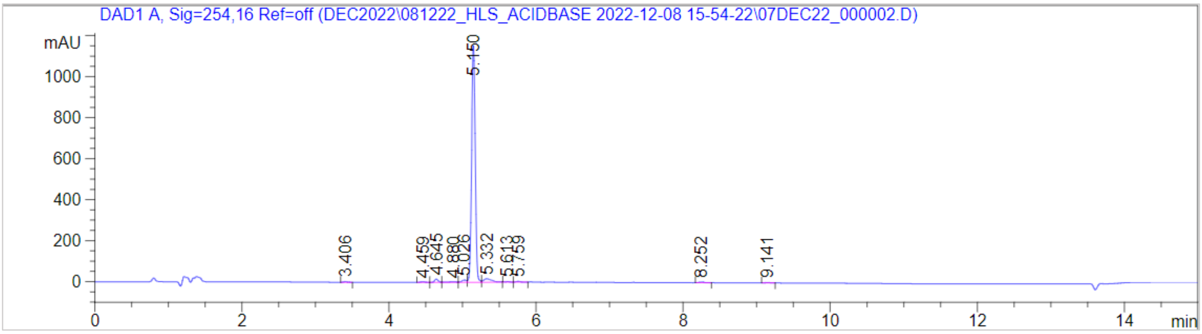

Peak area 94.2%
